# Aryl substitution of pentacenes

**DOI:** 10.3762/bjoc.10.178

**Published:** 2014-07-28

**Authors:** Andreas R Waterloo, Anna-Chiara Sale, Dan Lehnherr, Frank Hampel, Rik R Tykwinski

**Affiliations:** 1 Department of Chemistry and Pharmacy & Interdisciplinary Center for Molecular Materials (ICMM), University of Erlangen-Nürnberg (FAU), Henkestrasse 42, 91054 Erlangen, Germany; 2 Department of Chemistry, University of Alberta, Edmonton, AB T6G 2G2, Canada

**Keywords:** carbon-nanomaterials, organic semiconductor, pentacene, π-stacking, polycyclic aromatic hydrocarbon, solid-state structure

## Abstract

A series of 11 new pentacene derivatives has been synthesized, with unsymmetrical substitution based on a trialkylsilylethynyl group at the 6-position and various aryl groups appended to the 13-position. The electronic and physical properties of the new pentacene chromophores have been analyzed by UV–vis spectroscopy (solution and thin films), thermoanalytical methods (DSC and TGA), cyclic voltammetry, as well as X-ray crystallography (for 8 derivatives). X-ray crystallography has been specifically used to study the influence of unsymmetrical substitution on the solid-state packing of the pentacene derivatives. The obtained results add to our ability to better predict substitution patterns that might be helpful for designing new semiconductors for use in solid-state devices.

## Introduction

**Figure 1:**
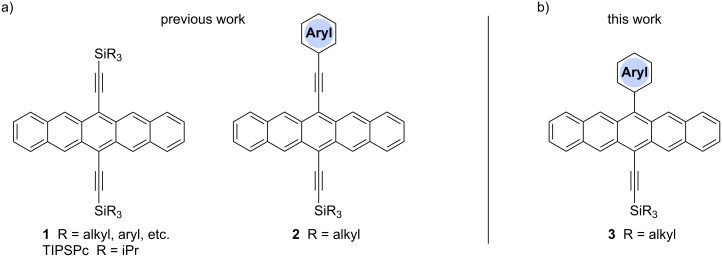
a) Examples of common pentacene functionalization patterns and b) unsymmetrically aryl-substituted pentacenes reported in this work.

Conjugated organic molecules are promising candidates for use in optoelectronic applications including OLEDs [1], photovoltaics [2], and OFETs [3]. Even though there is literally an infinite number of possibilities to chemically assemble small organic molecules for such applications, clever design and synthesis have rapidly offered new materials for the nascent era of molecular electronic [4–11]. Prominent p-type organic semiconductors include substituted acenes in general [12–15], and specifically 6,13-(triisopropylsilylethynyl)pentacene (TIPSPc) [13–14]. The latter was synthesized by Anthony over a decade ago [16], but it is still a leading focus of the scientific community. Substituted pentacenes can offer several advantages to other small molecule semiconductors, including stability, processability, and semiconductor device performance. Intermolecular π–π-interactions between chromophores can be dramatically improved upon functionalization at the 6- and 13-positions of pentacene, as demonstrated by the two-dimensional (2D) bricklayer-packing motif for TIPSPc [13–14]. A number of well-designed substitution patterns for the pentacene skeleton have been realized to date [17–25], and pentacene derivatives that provide good semiconductor device performance often show similar 6,13-substitution patterns (Figure 1a) [12,26]. The most reactive positions of the acene core are the 6- and 13-positions [27–29], and these positions can be effectively blocked by different trialkylsilylethynyl units [30]. Inspired by previous studies in which we [18,31], and others [32–34], observed promising solid-state arrangement and properties of aryl-substituted pentacenes, we were interested in the influence of different aryl moieties directly linked to the pentacene core. In this work we present a simple synthetic approach to unsymmetrically substituted pentacenes via stepwise functionalization of pentacenequinone, using a nucleophilic aryl group (Figure 1b). To determine the influence of aryl substitution, the obtained pentacenes have been studied by a variety of spectroscopic characterization methods as well as X-ray crystallography of eight derivatives. The studies reported herein offer an opportunity to interpret various properties of acenes based on their substitution and should contribute toward the use of such derivatives in materials science.

## Results and Discussion

### Synthesis and Characterization

#### Synthesis

**Scheme 1:**
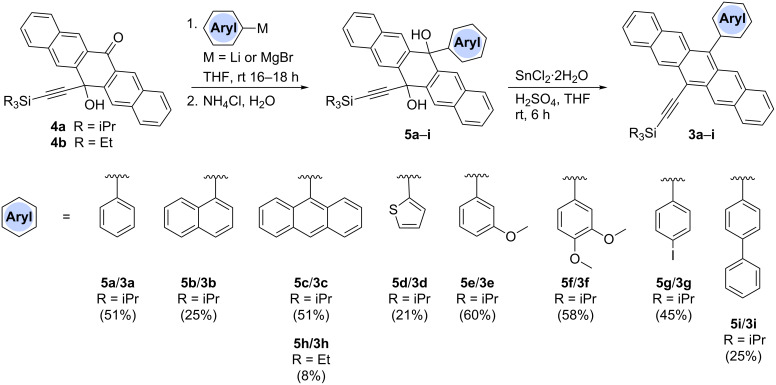
Synthesis of unsymmetrically substituted pentacenes by nucleophilic addition (yields given are for the pentacene product **3**, over the two steps from either **4a** or **4b**).

The synthesis of arylpentacenes was developed based on the known ketone derivatives **4a** and **4b**, formed through the addition of an acetylide to pentacenequinone (Scheme 1) [21,35–38]. With these two ketones in hand, a second nucleophilic addition was initiated. Thus, commercially available aryl halides dissolved in dry THF were subjected to lithium halogen exchange at −78 °C using *n*-butyllithium. A substoichiometric amount of *n*-BuLi was used in each case to ensure complete consumption of the *n-*BuLi through Li–halogen exchange and thus avoid the possibility of competitive addition of the nucleophilic *n-*Bu anion to the ketone group of either **4a** or **4b**. After reaction with the appropriate aryl lithium species, the reaction was quenched with a proton source, and the resulting diol intermediates **5a–h** were carried on directly to reductive aromatization with SnCl_2_/H_2_SO_4_ without further purification, ultimately yielding pentacene products **3a**–**h**. While the isolation and characterization of diol products resulting from nucleophilic additions to pentacenequinone has been possible [17–18,34], previous work has shown that aromatized products were more easily purified by column chromatography and recrystallization following the last step [30–31,36]. Thus, it was deemed procedurally more efficient to eliminate the purification step of the intermediate diols. Once formed, pentacenes **3a**–**h** were obtained in moderate yield over two steps, as deep-blue solids.

**Scheme 2:**
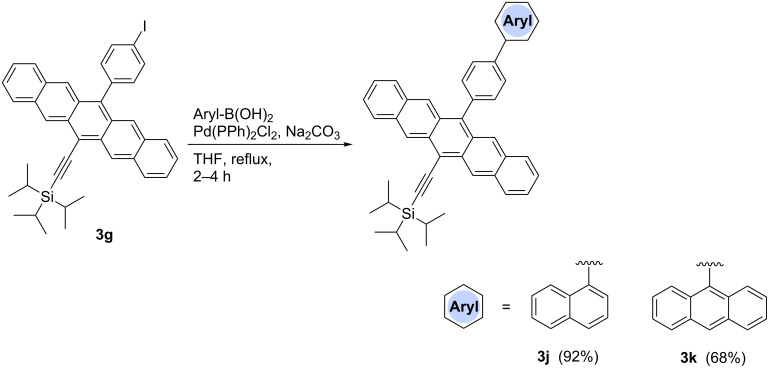
Functionalization of iodoaryl pentacene **3g** using the Suzuki–Miyaura cross-coupling reaction.

To expand the π-system in a linear fashion along the short molecular axis of the pentacene core, the general procedure described above was changed slightly, and ketone **4a** was treated with a solution of biphenylmagnesium bromide in THF. After work-up and isolation of the intermediate diol **5i**, reductive aromatization gave pentacene **3i** in moderate yield over the two steps. Elaborating on the general idea of lateral functionalization, iodoaryl pentacene **3g** offered an opportunity to vary the pendent substituent through a Pd-catalyzed cross-coupling protocol (Scheme 2). Thus, pentacene **3g** was treated under Suzuki–Miyaura coupling conditions with arylboronic acids, and the desired pentacenes **3j**,**k** were obtained in yields of 92 and 68%, respectively. Notably, anthracenyl-substituted pentacene **3k** was the least stable of all derivatives synthesized in this study. It slowly decomposed in solution when exposed to ambient laboratory conditions and was unstable toward silica gel chromatography. Compound **3k** could, however, be purified by recrystallization from a mixture of MeOH and acetone.

#### UV–vis spectroscopy

Aryl pentacenes **3a–k** have been studied by UV–vis absorption spectroscopy in CH_2_Cl_2_ solutions and as thin films cast from CH_2_Cl_2_ onto quartz. Solution-state UV–vis spectra show characteristic absorptions in the high-energy region with a maximum intensity absorption centered at ~310 nm, as well as low-energy absorptions at ~578 nm and ~622 nm. In comparison, unsubstituted pentacene (PEN) shows a λ_max_ = 576 nm (measured in benzene) [39], while the symmetrically substituted analogue TIPSPc shows a low-energy absorption at 643 nm (measured in CH_2_Cl_2_) due to extended conjugation through the two alkyne substituents [11].

**Figure 2:**
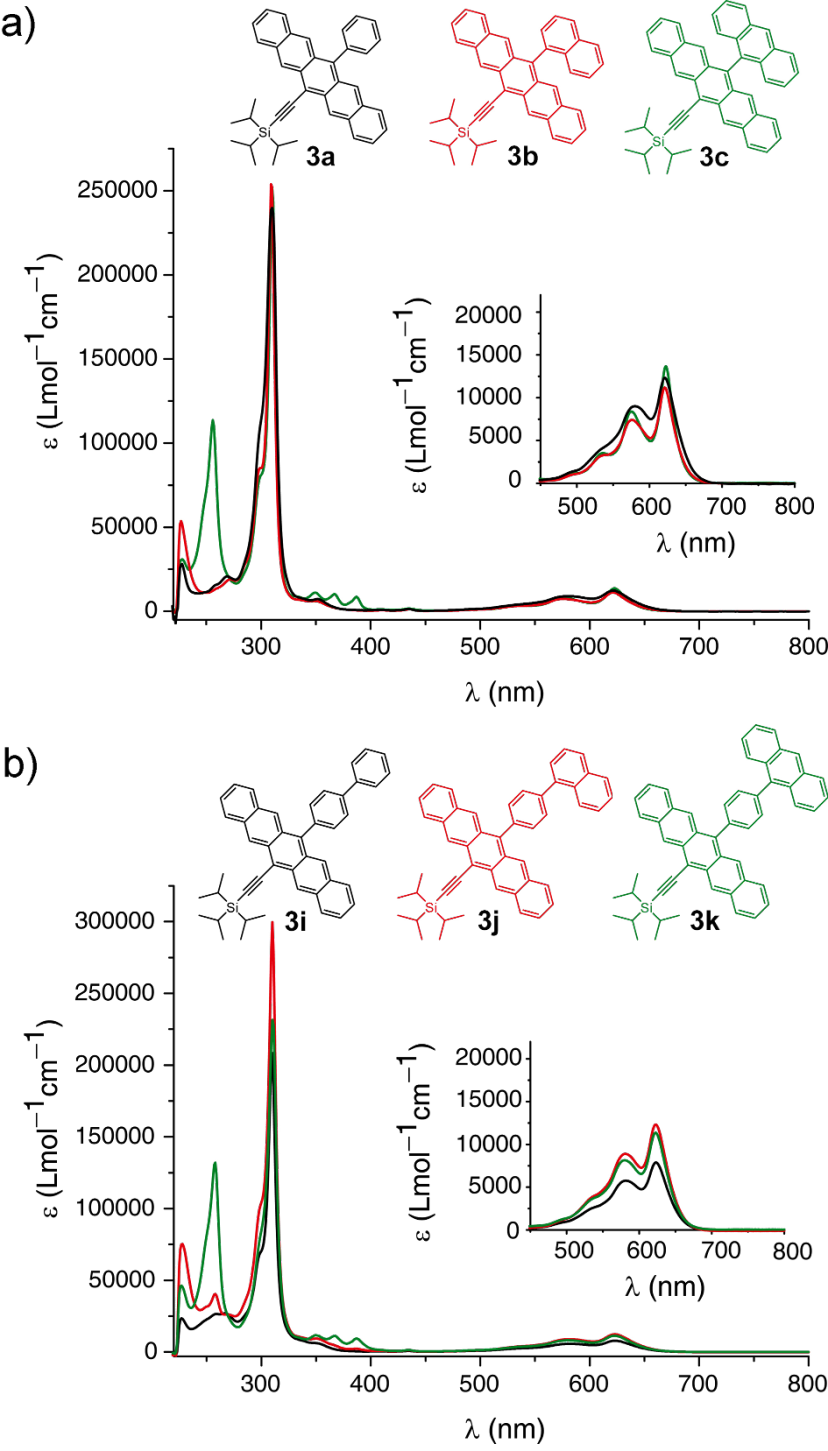
UV–vis spectra of pentacenes a) **3a**–**c** and b) **3i**–**k** (measured in CH_2_Cl_2_).

As can be seen in the representative spectra in Figure 2, the nature and size of aryl substituents at the 13-position does not alter the basic absorption wavelengths as one progresses, for example, through the series of phenyl (**3a**), naphthyl (**3b**) and anthracenyl (**3c**), although some differences in molar absorptivity are observed. Similar trends are observed within the series of pentacenes **3i**, **3j**, and **3k**. Thus, these UV–vis spectra clearly document the lack of communication between the aryl substituent and the pentacene unit, as a result of hindered rotation about the aryl–pentacene C–C bond and a preferred conformation in which the π-system of the aryl group is orthogonal to that of the pentacene. The orthogonal orientation of the aryl groups is also well-documented in the solid state by X-ray crystallographic analysis (vide infra). The fluorescence characteristics of **3a–k** are unremarkable, showing only minor variances in emission wavelength ranging from 652–671 nm, as measured in CH_2_Cl_2_ (see Supporting Information File 1 for details and spectra).

**Figure 3:**
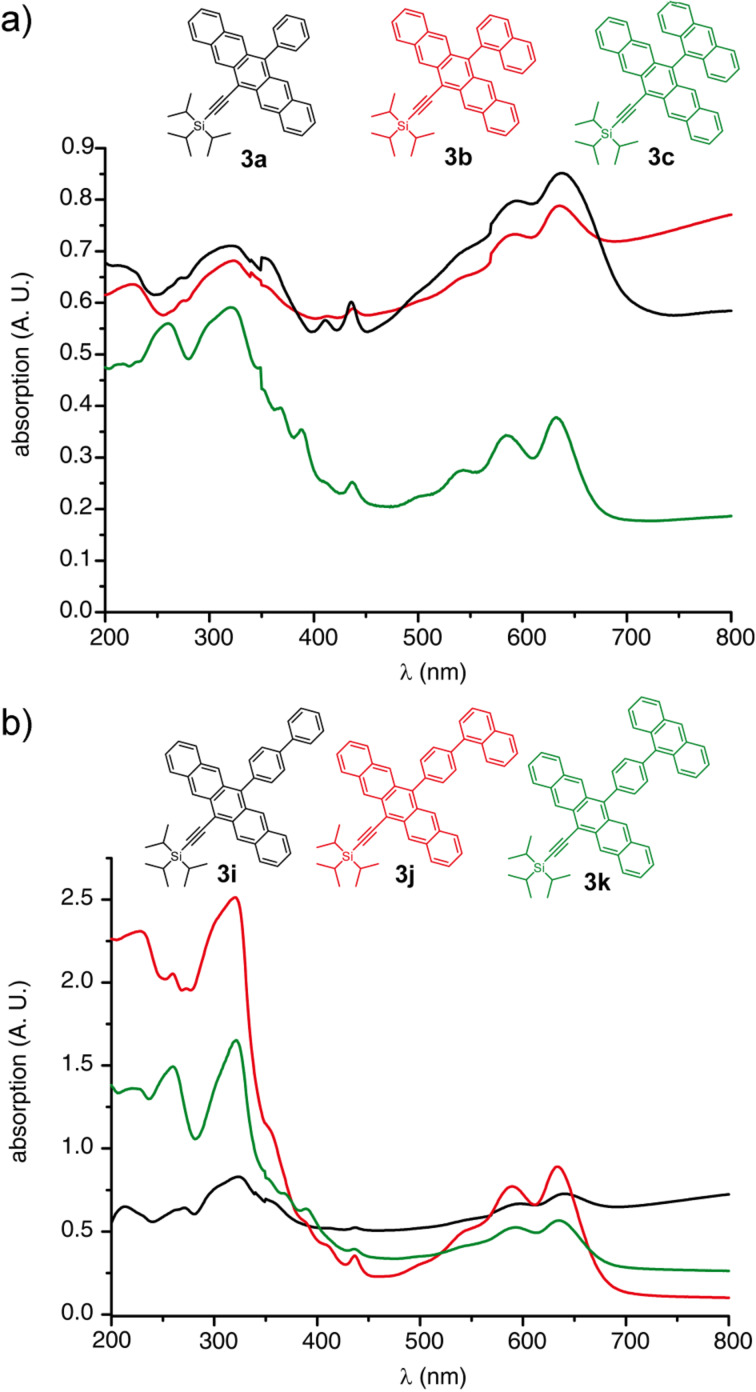
UV–vis spectra of thin films (drop cast on quartz from a CH_2_Cl_2_ solution) for pentacenes a) **3a**–**c** and b) **3i**–**k**.

The major electronic absorptions found in solution are also reproduced to a large extent in spectra obtained from solid-state films. Pentacene samples **3a**–**k** were drop-cast from a concentrated CH_2_Cl_2_ solution onto a quartz surface, and after air-drying, the absorption spectra were measured by UV–vis spectroscopy (Figure 3 and Table 1). While this method sometimes results in rather significant scattering versus the formation of films by spin-casting, only milligram quantities of material are required and the results are qualitatively informative (i.e., absorption wavelengths can be readily discerned, while determination of molar absorptivity is not possible). As can be seen in Figure 3, spectra from thin films show absorption profiles similar to those from solution-state measurements, although signals are broadened and absorptivities vary dramatically due to scattering. In the solid state, there are no observed absorptions at wavelengths beyond ca. 660 nm, and absorptions in the lower energy region show a red shift (7 to 33 nm) in comparison to analogous absorptions in solution. A red shift in the absorption features of samples in the solid state relative to those in solution is typically ascribed to a local electronic interaction between the respective pentacene molecules in the solid state. Of the aryl pentacenes studied here, veratrole derivative **3f** shows the largest red shift (33 nm) as a film compared to its solution-state UV–vis spectrum, although the origin of change in the solid state is not understood. It is worth noting, however, that more significant red-shifted λ_max_-values are often observed for samples which give solution cast films that result in significant π-stacking between molecules, such as TIPSPc, in which λ_max_ shifts from 643 nm in solution to ca. 705 nm in the solid film [11,35,40]. This same logic also suggests that the minimal difference between the absorption characteristics of **3c** and **3h** results from both a lack of influence from the different silyl groups, as well as the absence of strong π-stacking for both derivatives in the films, even though X-ray crystallographic analysis suggests that strong interactions might be possible for some derivatives in the solid state (vide infra).

**Table 1:** Optical properties of pentacenes **3a**–**k**, unsubstituted pentacene (PEN), and TIPSPc.

Compound	λ_max_ (in CH_2_Cl_2_)^a^ [nm]	λ_max_ (film)^b^ [nm]	red shift [nm] (meV)	*E*_gap,opt_ [eV]^c^

**3a**	621	637	16 (50)	1.89
**3b**	621	635	14 (44)	1.91
**3c**	623	637	14 (44)	1.92
**3d**	623	637	14 (44)	1.91
**3e**	621	634	13 (41)	1.89
**3f**	622	655	33 (100)	1.87
**3g**	621	628	7 (22)	1.90
**3h**	622	638	16 (50)	1.91
**3i**	623	641	18 (56)	1.88
**3j**	623	633	10 (31)	1.89
**3k**	623	635	12 (38)	1.89
PEN	576^d^	673^e^	97 (310)	2.15
TIPSPc	643^f^	705^g^	62 (170)	1.84^f^

^a^Lowest energy absorption maxima. ^b^Cast from CH_2_Cl_2_ onto quartz. ^c^Determined from solution-state spectra, based on a tangent line applied to the lower edge of the longest wavelength absorption peak and the intercept with the x-axis. ^d^Measured in benzene and data taken from [39]. ^e^Data taken from [41–42]. ^f^Data taken from [11]. ^g^Data taken from [35].

#### Thermal analysis

The thermal stability of selected aryl pentacenes has been explored by traditional melting point analysis (MPA) in open capillary tubes, thermal gravimetric analysis (TGA), and differential scanning calorimetry (DSC) measurements; the results are summarized in Table 2. TGA shows clearly that significant mass loss occurs in the range of 400 °C, which is also common for ethynylated pentacenes such as **1** and **2** [17–18]. There appears to be little evidence of a trend based on the size of the aryl group versus the temperature of observed mass loss in the TGA. By comparing the TGA data with that of MPA made in open capillary tubes, however, it is clear that all pentacene derivatives undergo either a phase change or decomposition prior to the mass loss observed in the TGA. This premise is also confirmed by DSC analyses, which show a melting point in all cases except for thienyl derivative **3d** (which decomposed directly in the solid state). In the case of **3a** and **3h**, melting is followed immediately by decomposition (DSC and TGA scans are provided in Supporting Information File 1).

**Table 2:** Thermal properties of a representative selection of pentacenes.

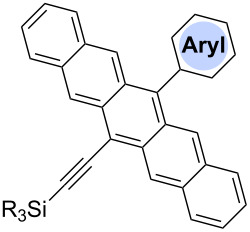					

Compound	R	Aryl	TGA *T*_d_ /°C^a^	MPA mp /°C^b^	DSC mp (DSC dp) /°C^c^

**3a**	iPr	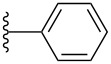	370	162–165	177 (178/179)
**3b**	iPr	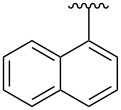	370	244–246	248
**3c**	iPr	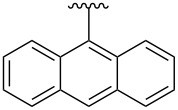	375	306–308^d^	197^e^
**3d**	iPr	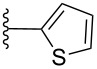	372	316–318^d^	– (206/247)
**3h**	Et	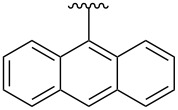	410	291–293	287 (288/290)
**3i**	iPr	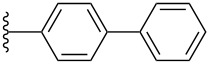	380	211–214	225
**3j**	iPr	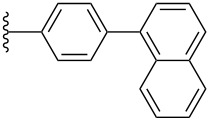	380	233–235	220^f^

^a^Measured under a nitrogen atmosphere. *T*_d_ = decomposition temperature, see Supporting Information File 1 for details. ^b^Traditional open capillary melting point analysis (MPA), measured under ambient conditions; uncorrected. ^c^Measured under a nitrogen atmosphere; dp = decomposition point, shown as onset/peak temperatures. ^d^Decomposition observed in that temperature range, with no indication of melting or decomposition at lower temperature. ^e^Endotherm, although apparently not a true mp based on traditional mp analysis. Exotherm at 286 °C (peak) likely corresponds to dp in DSC. ^f^The strongest of several endotherms.

While no correlation between pendent substituent and stability emerges from this analysis, an important point is nevertheless noted, as exemplified by the examination of **3c** and **3d**. Traditional MPA is often insufficient for characterization of pentacene derivatives, in which subtle changes in the samples can be difficult to discern because of the deep, dark color of the sample, and conflicting results are often observed between MPA and DSC.

#### Cyclic voltammetry

Electrochemical analysis by cyclic voltammetry (CV) was used to investigate the electronic properties of pentacene derivatives **3a**–**k** in CH_2_Cl_2_ (ca. 1.5 mM) using tetrabutylammonium hexafluorophosphate as supporting electrolyte and ferrocene as internal standard (all potentials reported are thus given versus Fc/Fc^+^). Aryl-substituted pentacenes **3a**–**k** each show a one-electron reversible oxidation event in the range between 0.30–0.37 V (Table 3), and a second oxidation process (quasi-reversible) in the range of 0.80–0.99 V. There is, unfortunately, no clear trend observed for the oxidation potentials based on the substitution pattern of the aryl moieties, although both oxidations appear somewhat easier for pentacene **3f** as a result of the two electron-donating methoxy groups attached to the pendent phenyl ring.

**Table 3:** Electrochemical properties of **3a**–**k** compared to TIPSPc and **2a**–**c**.^a^

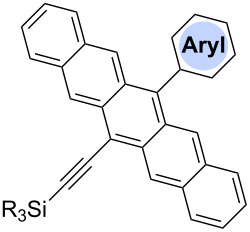						

Compound	R	Aryl	*E*_ox1_ [V]	*E*_ox2_ [V]	*E*_red1_ [V]	*E*_gap,el_ [eV]^b^

**3a**	iPr	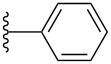	0.34	0.87	−1.63	1.97
**3b**	iPr	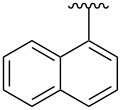	0.37	0.99	−1.61	1.98
**3c**	iPr	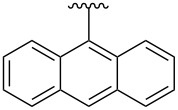	0.36	0.93	−1.65	2.01
**3h**	Et	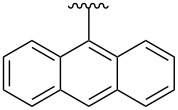	0.32	0.91	−1.68	2.00
**3d**	iPr	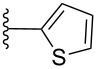	0.35	0.87	−1.59	1.94
**3e**	iPr	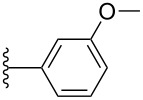	0.32	0.87	−1.68	2.00
**3f**	iPr	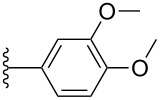	0.30	0.80	−1.67	1.97
**3g**	iPr	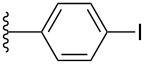	0.34	0.87	−1.65	1.99
**3i**	iPr	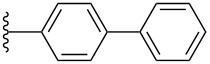	0.32	0.87	−1.66	1.98
**3j**	iPr	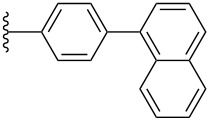	0.32	0.93	−1.67	1.99
**3k**	iPr	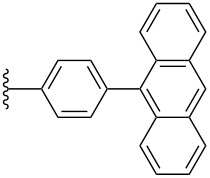	0.35	0.88	−1.67	2.02
**2a**^c^	iPr	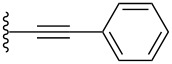	0.39	–	−1.44	1.83
**2b**^c^	iPr	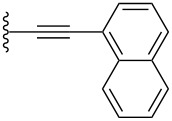	0.39	–	−1.42	1.81
**2c**^c^	iPr	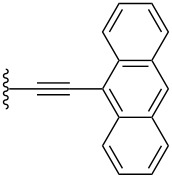	0.33	–	−1.38	1.71
TIPSPc	iPr	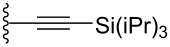	0.39	0.99	−1.52	1.91

^a^Cyclic voltammetry was performed in CH_2_Cl_2_ solutions (1.5 mM) containing 0.1 M *n*-Bu_4_NPF_6_ as supporting electrolyte at a scan rate of 150 mV/s. Platinum wire was used as counter electrode, Ag/AgNO_3_ as reference electrode, and Pt working electrode. The potential values (*E*) were calculated using the following equation *E* = (*E*_pc_ + E_pa_)/2, where *E*_pc_ and *E*_pa_ correspond to the cathodic and anodic peak potentials, respectively. Potentials are referenced to the ferrocene/ferrocenium (Fc/Fc^+^) couple used as an internal standard. All potentials represent a reversible one-electron reduction or oxidation event. ^b^Electrochemical HOMO–LUMO gaps determined by *E*_gap,el_ = *E*_ox1_ − *E*_red1_. ^c^Data taken from [18].

Aryl-substituted pentacenes **3a**–**k** each show one reversible reduction event in a rather narrow range of −1.59 to −1.68 V. Similar to that observed for the oxidation potentials, there is no obvious trend that can be identified in the reduction potentials based on substitution pattern, aside from the observation that the silyl substituent might have a slight impact on reduction (**3h** is slightly harder to reduce than **3c**), and the reduction of thienyl derivative **3d** (−1.59 V) stands out as lower than the others.

Substituted pentacenes **3a–k** are slightly easier to oxidize than TIPSPc (*E*_ox1_ = 0.39 V), and the *E*_ox1_ values of **3a**–**k** fall into a similar range as found for pentacene-based PAH dyads **2a**–**c** in which the pendant aryl groups are linked by an ethynyl spacer that allows electronic communication between the two arenes [18]. The range of oxidation potentials between TIPSPc, **2a**–**c**, and **3a**–**k** is, however, quite narrow, suggesting that the pendent substituent offers little influence on the HOMO level. On the other hand, there is a marked difference in the observed reduction potentials. Compounds **2a**–**c** are most easily reduced while **3a–k** are the most difficult, and the reduction of TIPSPc falls at approximately a midpoint between the two other classes. Thus, the biggest influence of the pendent substituent appears to be related to the energy of the LUMO.

As suggested by the UV–vis analyses (vide infra), the HOMO–LUMO gap estimated for pentacenes **3a**–**k** by CV (1.94–2.02 eV) is larger than the HOMO–LUMO gap of TIPSPc (1.91 eV), while incorporation of the ethynyl spacer in **2a**–**c** provides for the lowest HOMO–LUMO gap of the molecules discussed here.

#### X-ray crystallographic analysis

**Figure 4:**
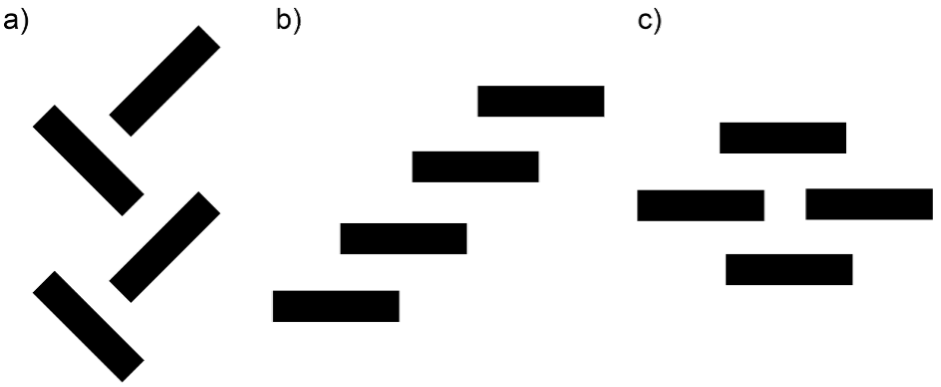
Schematic classification of three common solid-state arrangements of pentacene derivatives a) herringbone packing, b) 1D slipped-stack packing, and c) 2D “bricklayer” packing (as viewed side-on, approximately down the short-molecular axis).

Typically, three predominant solid-state packing patterns are found by X-ray crystallographic analysis of pentacene and its derivatives [13]: a) a herringbone packing, b) a one-dimensional (1D) slipped-stack, and c) a 2D “bricklayer” packing, as schematically summarized in Figure 4. While several polymorphs have been reported for unsubstituted pentacene [43–46], the arrangement in the solid state is commonly the edge-to-face, herringbone motif [47]. This packing arrangement provides strong electronic coupling in the solid state, and therefore makes this material interesting as the active component for semiconducting devices [48]. It has been shown that functionalization of the pentacene framework, particular by trialkylsilylethynyl groups, drastically alters the solid-state arrangements of the acenes [30]. In certain cases, this leads to a 2D face-to-face “bricklayer” arrangement, which can potentially facilitate charge transport in an electric device by several orders of magnitude [49].

**Figure 5:**
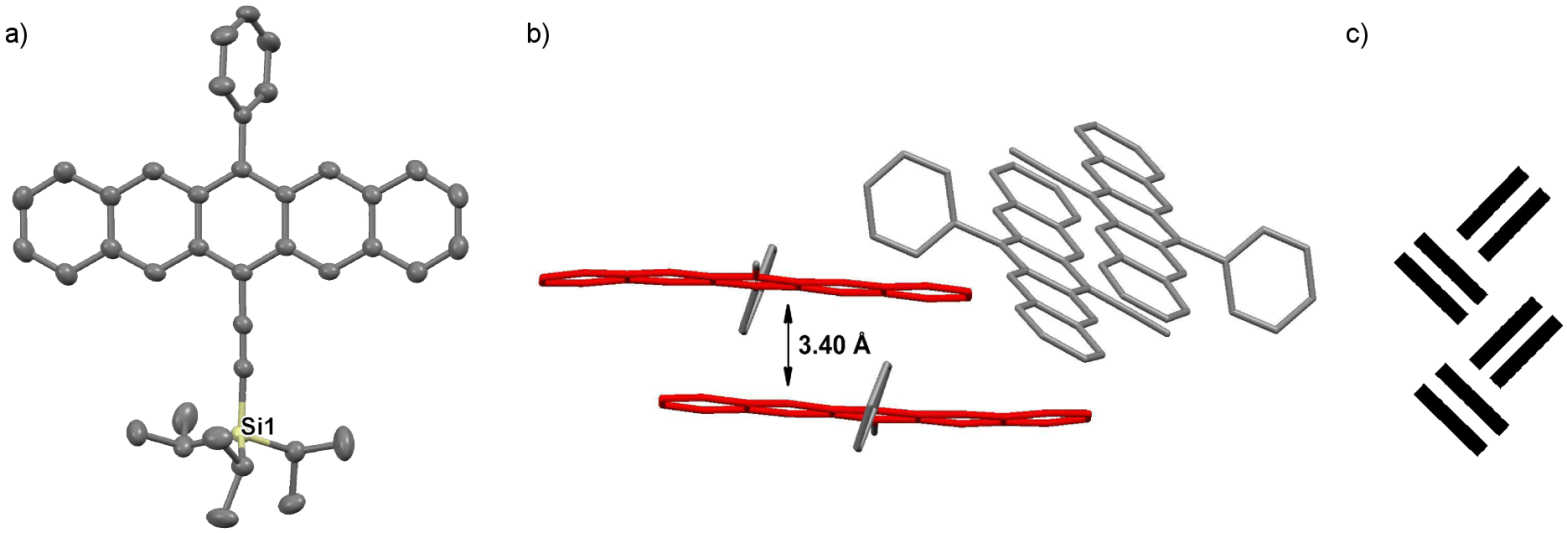
X-ray crystallographic analysis of **3a** showing a) molecular structure and b) packing motif (triisopropylsilyl groups omitted for clarity); ORTEP drawn at 50% probability level. c) Schematic representation of “sandwich herringbone” packing arrangement.

With the understanding that the solid-state packing of acenes can provide vital information about intermolecular interactions, single crystals of pentacenes **3a**–**d** and **3g**–**j** were grown and their solid-state arrangements determined by single crystal X-ray diffraction analysis (crystallographic details are provided in Supporting Information File 1). Pentacene **3a** crystallizes in the space group *C*2/*c* with eight molecules in the unit cell (Figure 5). Within the solid-state structure, the pendant phenyl ring and the pentacene core are slightly twisted, with a torsion angle of ~71°. Molecules of pentacene **3a** arrange as dimeric pairs, which then pack into a so-called sandwich herringbone motif (Figure 5c) [50]. Each dimeric pair of pentacenes shows face-to-face π-stacking interactions with an interplanar distance of ~3.40 Å and a total overlap of nearly four of the aromatic pentacene rings.

**Figure 6:**
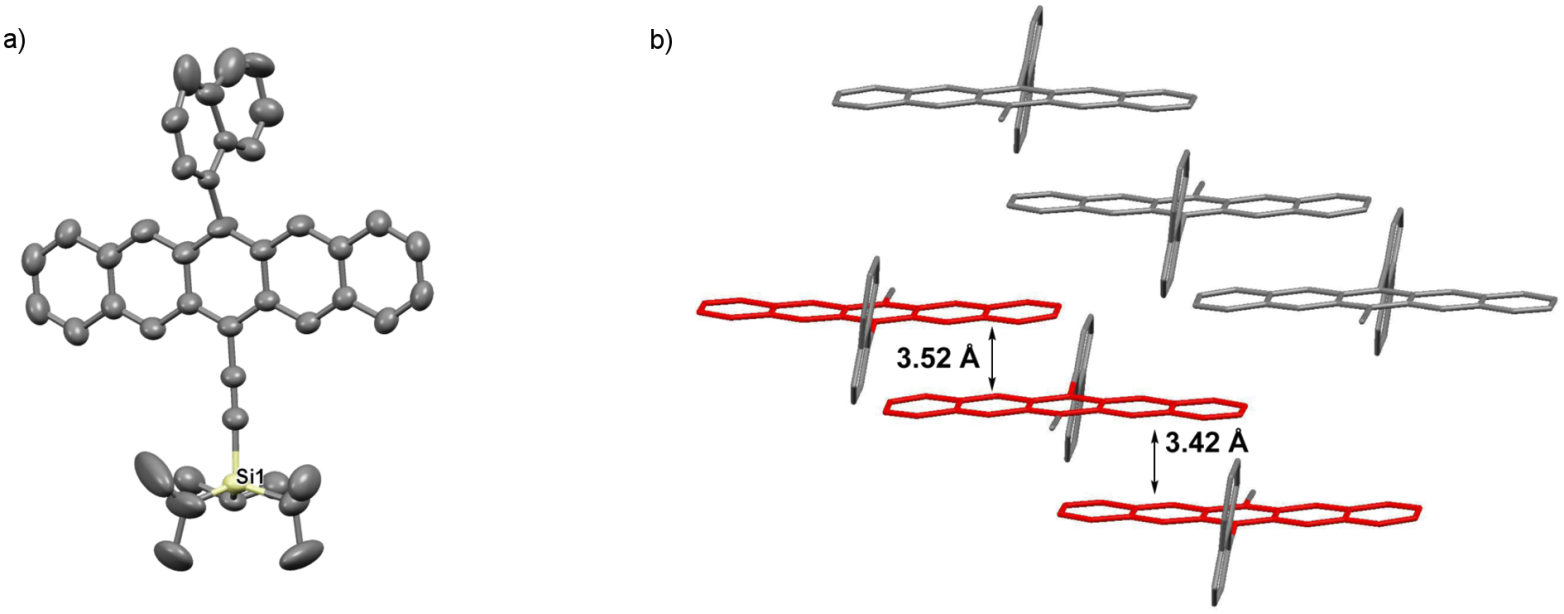
X-ray crystallographic analysis of **3b** showing a) molecular structure and b) packing motif (triisopropylsilyl groups omitted for clarity); ORTEP drawn at 50% probability level.

Pentacene **3b** crystallizes in the space group *P*-1 with two molecules in the unit cell (Figure 6). Notably, the naphthyl unit is significantly disordered over two unique positions in the solid state but is nearly perpendicular to the pentacene core with a twist angle of ~81°. Pentacene **3b** assembles in dimeric pairs, which then arrange in a 1D π-slipped stack motif, and the pentacene molecules are separated by interplanar distances of ~3.42 Å and ~3.52 Å.

**Figure 7:**
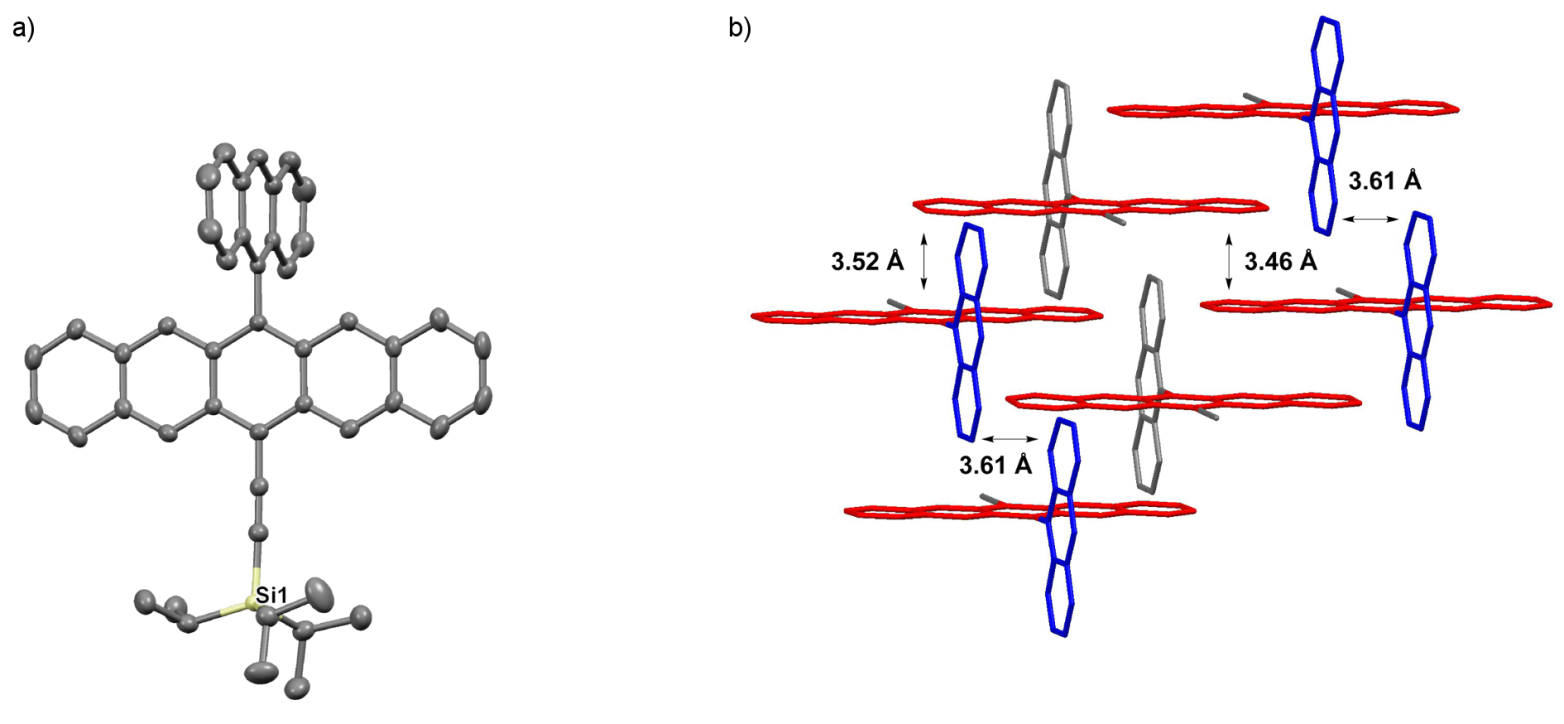
X-ray crystallographic analysis of **3c** showing a) molecular structure and b) packing motif (triisopropylsilyl groups omitted for clarity); ORTEP drawn at 50% probability level.

Pentacene **3c** crystallizes in the space group *P*2_1_/*c* with four molecules in the unit cell (Figure 7) [31]. The pentacene skeleton and the anthracenyl substituent are nearly perpendicular to each other with a twist angle of ~90°. This motif also places the anthracene moieties in a face-to-face packing 1D slipped stack arrangement, although the interplanar distance of ~3.61 Å is sizable. The aromatic pentacene cores pack in a face-to-face 2D bricklayer arrangement, with approximately two pentacene rings overlapping and interplanar distances of ~3.52 Å and 3.46 Å.

**Figure 8:**
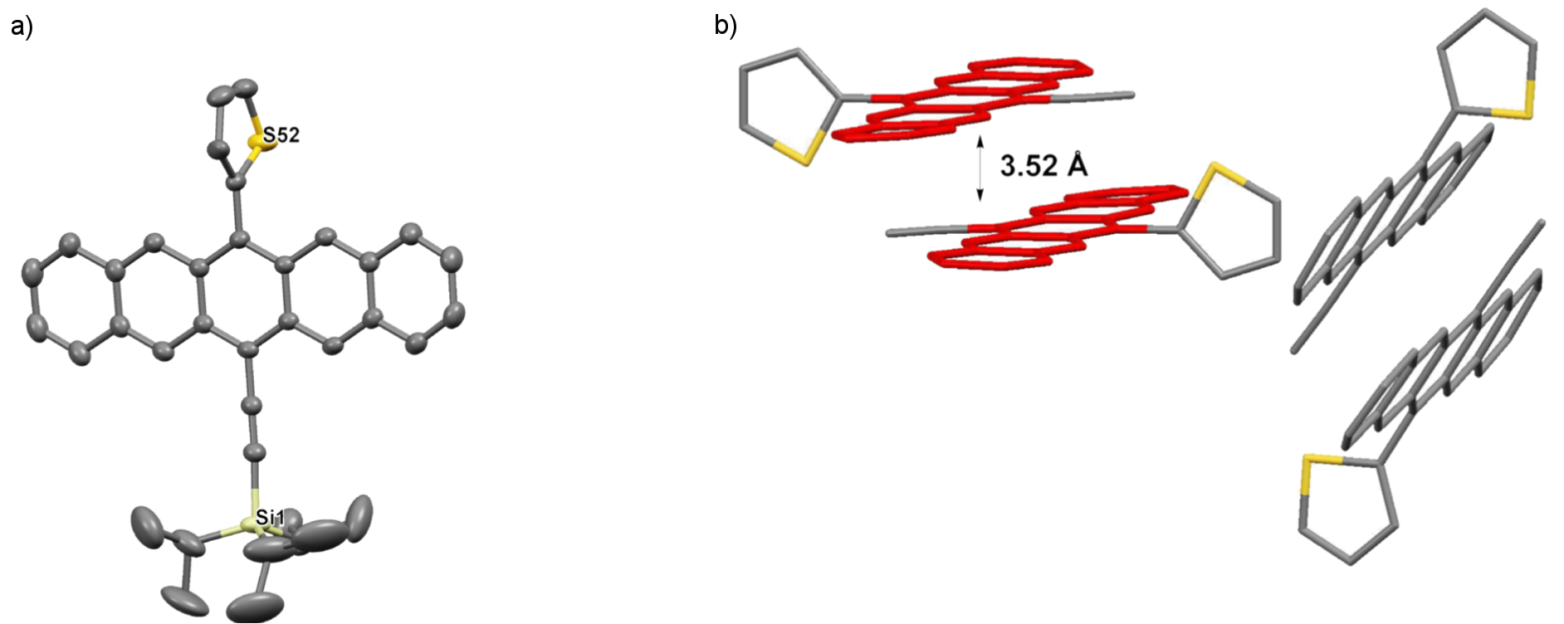
X-ray crystallographic analysis of **3d** showing a) molecular structure, and b) packing motif (triisopropylsilyl groups omitted for clarity); ORTEP drawn at 50% probability level.

Pentacene **3d** crystallizes in the space group *P*2_1_/*n* with four molecules in each unit cell (Figure 8), and the thienyl and trialkylsilyl groups show disorder in the structure. With a twist angle of ~90°, the thienyl unit is essentially perpendicular to the pentacene skeleton. Centrosymmetric dimeric pairs of pentacene **3d** pack with an interplanar distance of 3.52 Å and these pairs then arrange into a sandwich herringbone stacking pattern, similar to phenyl derivative **3a**.

**Figure 9:**
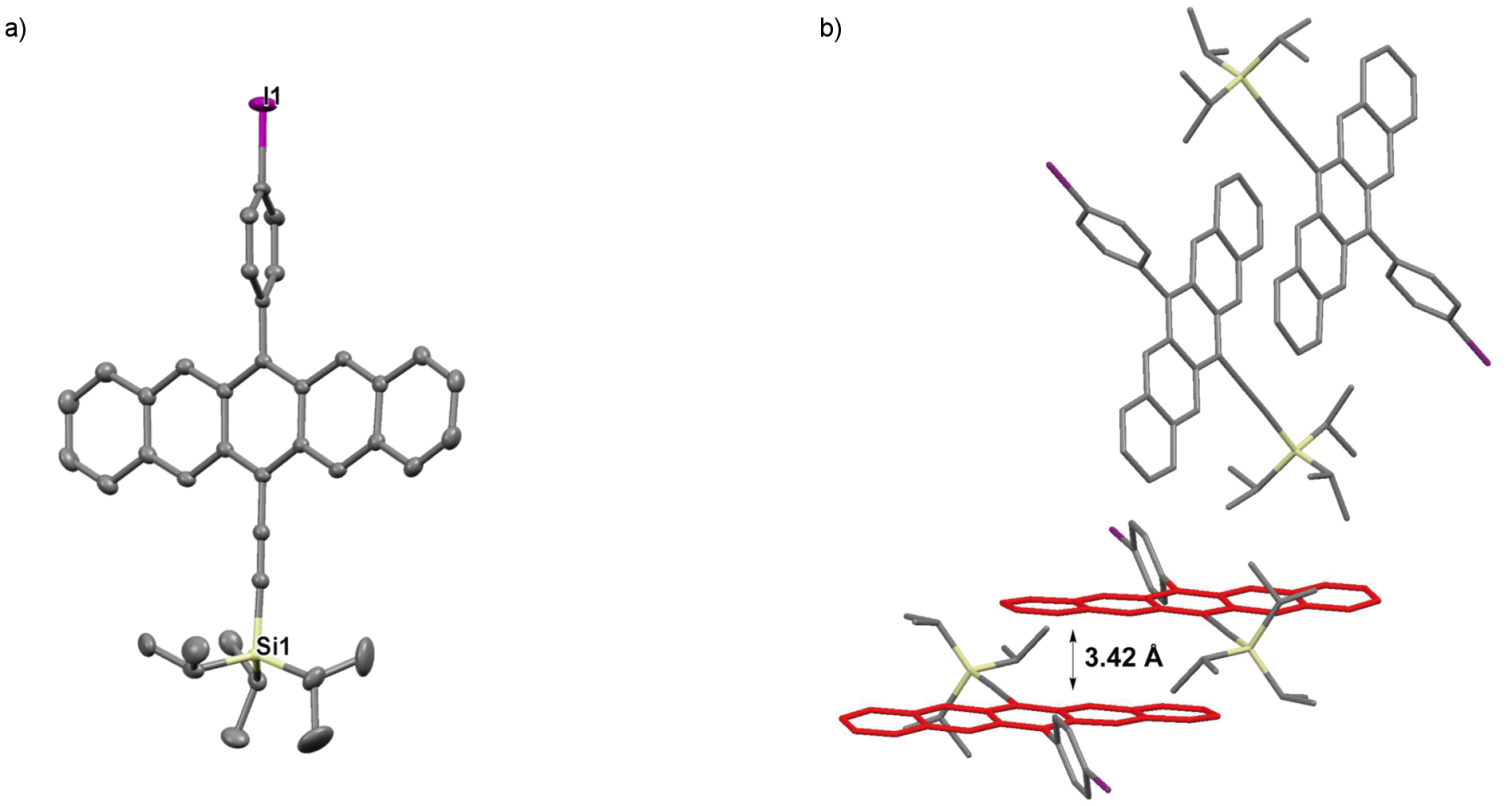
X-ray crystallographic analysis of **3g** showing a) molecular structure and b) packing motif; ORTEP drawn at 50% probability level.

Pentacene **3g** crystallizes in the space group *P*2_1_/*n* with four molecules in the unit cell (Figure 9). The phenyl substituent is twisted with an angle of ~70° relative to the pentacene skeleton. Two neighboring molecules of **3g** arrange into a dimeric pair with an interplanar distance of 3.42 Å, and these pairs then pack in a sandwich herringbone arrangement. The overall solid-state arrangement is similar to that observed for **3a** and **3d**.

**Figure 10:**
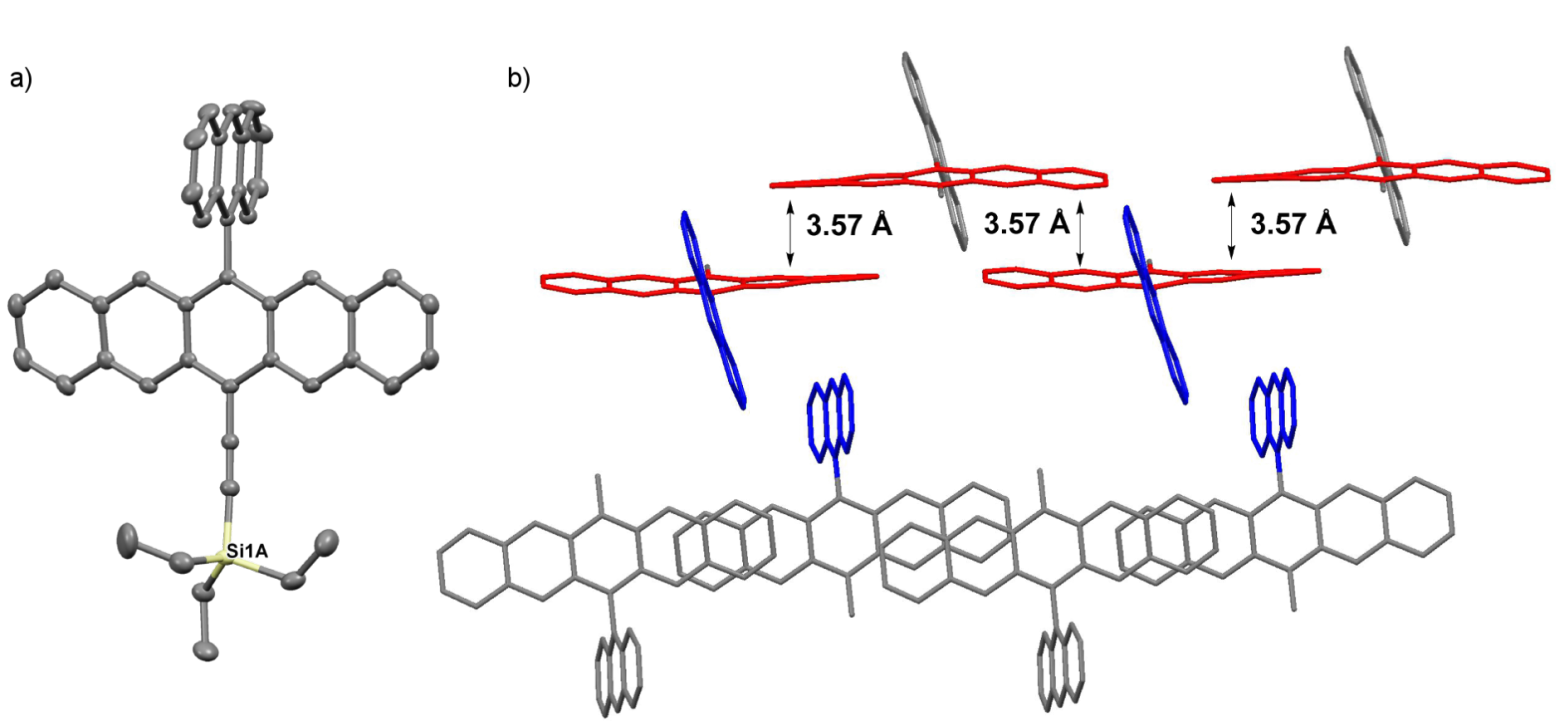
X-ray crystallographic analysis of **3h** showing a) molecular structure and b) packing motif (triethylsilyl groups omitted for clarity); ORTEP drawn at 50% probability level.

Pentacene **3h** crystallizes in the space group *P*2_1_/*c* with four molecules in each unit cell (Figure 10). The anthracenyl substituent is twisted relative to the pentacene skeleton with an angle of ~74°, less than that found for **3c** (90°). Interestingly, pentacene **3h** shows an unusual solid-state arrangement not typically observed for pentacene derivatives. Namely, the pentacene molecules form channels along the crystallographic *a*-axis, which are composed of only two tiers of a brick wall structure. The pentacene molecules within these channels are stacked with an interplanar distance of 3.57 Å. These channels are macroscopically arranged as staircases, dictated by the anthracenyl moieties that are oriented such that CH–π interactions of ~2.90 Å likely play a role in directing the packing (see Supporting Information File 1, Figure S12).

**Figure 11:**
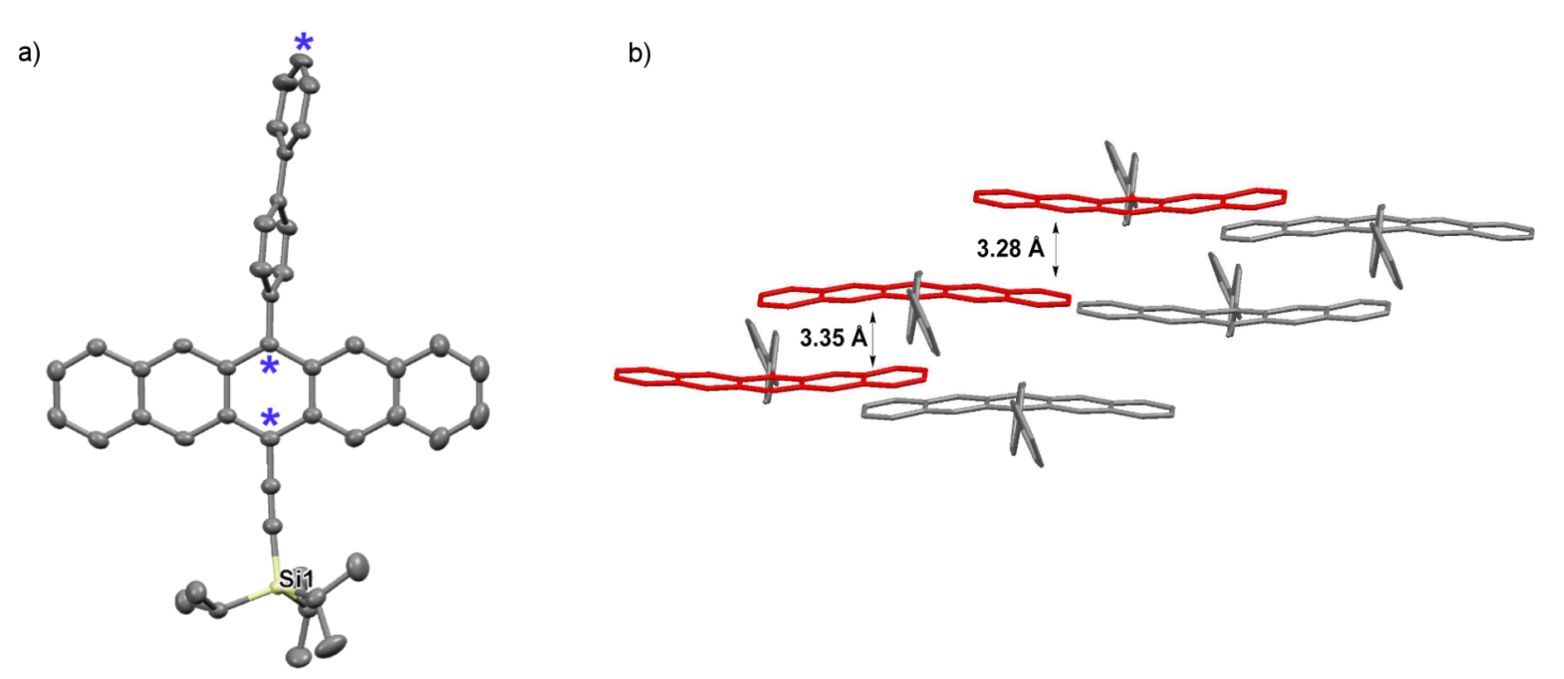
X-ray crystallographic analysis of **3i** showing a) molecular structure and b) packing motif (triisopropylsilyl groups omitted for clarity); ORTEP drawn at 50% probability level.

Pentacene **3i** crystallizes in the space group *P*-1 with two molecules in each unit cell (Figure 11). The benzene ring directly attached to the pentacene framework is nearly perpendicular to the pentacene core with an angle of ~81°, while the torsion angle between the two rings of the biphenyl unit is 32°. The biphenyl substituent is slightly bent from linearity with an angle of ~6° (as estimated from the three atoms designated with an asterisk *). Biphenyl-substituted pentacene **3i** arranges in a 1D slipped stack motif along the crystallographic *c-*axis, with π*-*stacking distances of 3.28 Å and 3.35 Å.

**Figure 12:**
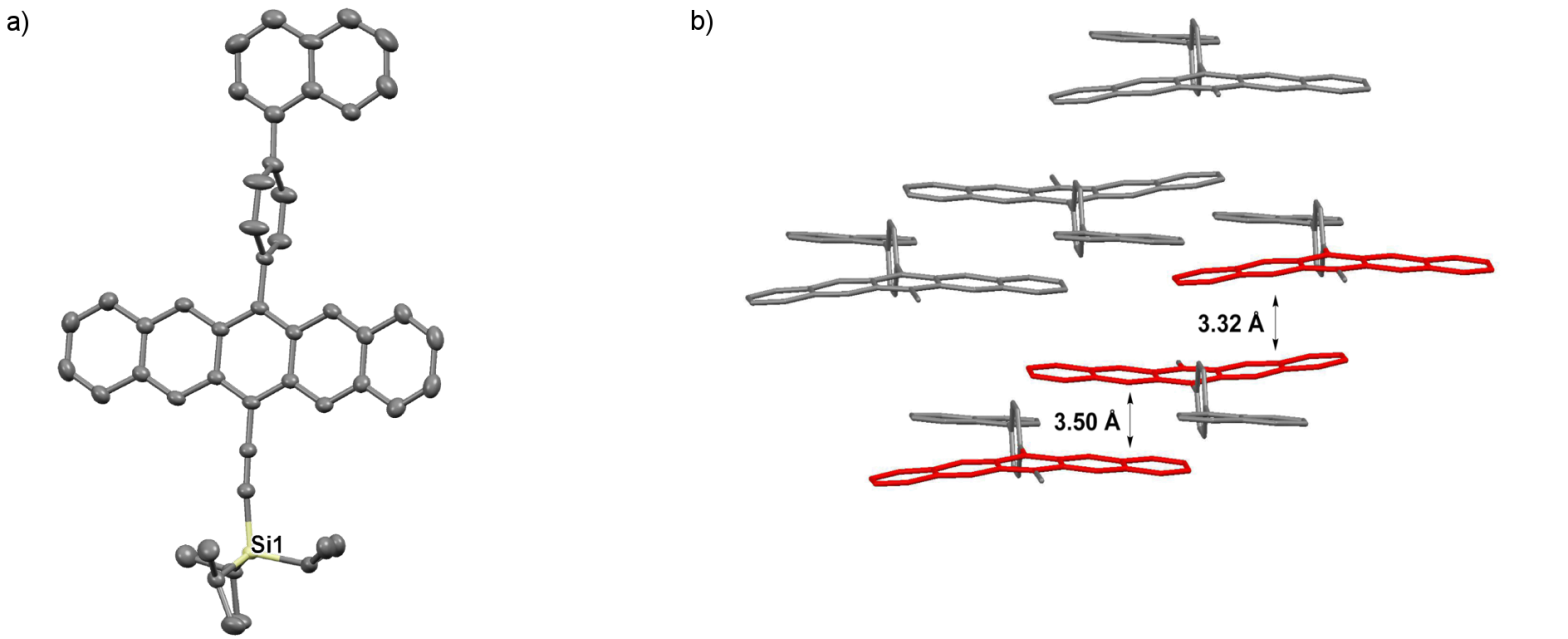
X-ray crystallographic analysis of **3j** showing a) molecular structure and b) packing motif (triisopropylsilyl groups omitted for clarity); ORTEP drawn at 50% probability level.

Pentacene derivative **3j** crystallizes in the space group *P*-1 with two molecules in the unit cell (Figure 12). Interestingly, the pentacene core and the naphthyl group are nearly coplanar (4°), while the intervening benzene ring is nearly perpendicular to both the pentacene skeleton (~90°) and the naphthyl group (~86°). Compound **3j** arranges in a 1D slipped stack arrangement along the crystallographic *b*-axis with two different interplanar distances of ~3.50 Å and 3.32 Å.

## Conclusion

In summary, a library of unsymmetrically substituted pentacenes has been synthesized by a straightforward procedure that requires only one purification step. Optical spectroscopy and cyclic voltammetry reveal that electronic communication between the pentacene core and the different substituents is limited, as a result of the orthogonal orientation of the pentacene backbone and the pendent aryl moieties. Thus, these results show that the nature of the substituent does not change the electronic properties of the pentacene skeleton itself. Aryl-substitution pattern does however, have a considerable effect on solid-state arrangement of the molecules, and X-ray crystallographic analysis afforded insight on the packing arrangements of the synthesized pentacenes. In spite of the large number of crystallographic analyses that have been examined here, general trends are difficult to establish based on, for example, either the number of π–π and CH–π interactions or the size of the aromatic group appended to the pentacene core. It does seem, however, that anthracenyl substitution (in **3c** and **3h**) affords the highest degree of π-stacking amongst the derivatives examined.

## Supporting Information

File 1 Experimental procedures and characterization data for all new compounds. Copies of ^1^H and ^13^C NMR, UV–vis, and emission spectra; CV, DSC, and TGA scans.
