# Supplementary material for: Aryl substitution of pentacenes
Source: Beilstein J Org Chem. 2014 Jul 28;10:1692–705. doi: 10.3762/bjoc.10.178 (PMC4142869; doi:10.3762/bjoc.10.178)
Supplement: File 1 — Experimental procedures and characterization data for all new compounds. Copies of 1H and 13C NMR, UV–vis, and emission spectra; CV, DSC, and TGA scans. [file Beilstein_J_Org_Chem-10-1692-s001.pdf]

Supporting Information

for

Aryl substitution of pentacenes

Andreas R. Waterloo[1], Anna-Chiara Sale[1], Dan Lehnherr[2], Frank Hampel[1], and Rik R. Tykwinski[1]*

[1]Department of Chemistry and Pharmacy & Interdisciplinary Center for Molecular Materials (ICMM), University of Erlangen-Nürnberg (FAU), Henkestrasse 42, 91054 Erlangen, Germany and [2]Department of Chemistry, University of Alberta, Edmonton, AB T6G 2G2, Canada

Email: Rik R. Tykwinski - rik.tykwinski@fau.de

*Corresponding author

Experimental procedures and characterization data for all new compounds.

Copies of ^{1}H and ^{13}C NMR, UV–vis, and emission spectra;

CV, DSC, and TGA scans

Table of Contents for Supporting Information

General experimental details	S3
Synthetic procedures and spectral characterization of new compounds	S4
UV-Vis and emission spectra of pentacenes **3a**–**k**	S17
Packing diagram of pentacene **3h**	S24
Cyclic voltammograms of pentacenes **3a**–**k**	S25
DSC and TGA scans of pentacenes **3a**–**d** and **3i**–**j**	S27
References	S34
^{1}H and ^{13}C NMR Spectra	S35

General Experimental

Reagents were purchased in reagent grade from commercial suppliers and used without further purification. THF was distilled from sodium/benzophenone. All reactions were performed in standard, dry glassware. Column chromatography: silica gel-60 (230–400 mesh). Thin Layer Chromatography (TLC): pre-coated plastic sheets covered with 0.20 mm silica gel with fluorescent indicator UV 254 nm; visualization by UV light. Melting points are uncorrected. ^{1}H- and ^{13}C-NMR spectra were collected at rt in $CDCl_3$; solvent peaks (7.24 for ^{1}H and 77.0 for ^{13}C) as reference. Coupling constants are reported as observed (±0.5 Hz).

UV–vis absorption spectra were acquired at rt using a Varian Cary 5000 spectrophotometer (for all the solution-state and solid-state data); λ_{max} in nm (ε in L•mol^{-1}•cm^{-1}). Emission spectra were recorded using a Horiba Jobin Yvon fluoromax-4 spectrofluorometer; solutions used for emission measurements were deoxygenated by bubbling N_2 through the solvent. For mass spectral analyses, low-resolution data are provided in cases when M^+ is not the base peak; otherwise, only high-resolution data are provided.

Differential scanning calorimetry (DSC) measurements were made on a Mettler Toledo TGA/ STDA 851e/1100/SF. Thermogravimetric analyses (TGA) were achieved on a Mettler Toledo DSC 821e/ Sensor FRS5-Ceramic. All thermal analyses were carried out under a flow of nitrogen with a heating rate of 10 °C/min. Thermal decomposition temperature as measured by TGA (as sample weight loss) is reported as T_d in which the temperature listed corresponds to the intersection of the tangent lines of the baseline and the edge of the peak corresponding to the first significant weight loss, typically >5%. Melting points from DSC analysis are reported as the peak maxima, except in cases when the sample decomposed, in which case the onset temperature of the decomposition exothermic peak is reported, as well as the exothermic maxima corresponding to the decomposition.

X-ray data for **3a** (CCDC 985357), **3b** (CCDC 985358), **3d** (CCDC 985362), **3g** (CCDC 985359), **3h** (CCDC 985363), **3i** (CCDC 985361), **3j** (CCDC 985360), and **4b** (CCDC 985834) have been deposited at the Cambridge Crystallographic Data Centre (CCDC), 12 Union Road, Cambridge CB21EZ, UK; fax: (+44)122-333-6033. These data can be obtained free of charge from The Cambridge Crystallographic Data Centre via the Internet at www.ccdc.cam.ac.uk/data_request/cif using the CCDC numbers given above.

General Procedure A. Formation of aryl pentacenes 3a–i.

Unless otherwise noted, to a solution of the corresponding aryl halide (3 equiv, ca. 3 mmol scale) in dry, deoxygenated THF (20 mL) at –78 °C was added *n*BuLi (2.5 M in hexanes, 2.9 equiv).† After stirring for 30 min at –78 °C, ketone **4** (1 equiv) was added as a solid. The resulting mixture was stirred for 12–14 h at rt. The reaction was quenched by the addition of saturated aq NH_4Cl (100 mL) and extracted with CH_2Cl_2 (3 x 50 mL). The combined organic phases were washed with brine (100 mL), dried over Na_2SO_4, filtered, and the solvent removed *in vacuo*. This residue containing the diol **5** was dissolved in THF (20 mL) and subjected to reductive aromatization by the addition of $SnCl_2$•$2H_2O$ (3 equiv) and 10% aqueous H_2SO_4 (1 mL). The mixture was stirred for 4–6 h at rt and saturated aq NH_4Cl (100 mL) was then added. The mixture was extracted with CH_2Cl_2 (3 x 50 mL; if the phases do not separate and/or the mixture forms an emulsion, then ~10 mL of conc HCl were carefully added to the mixture in the separation funnel). The combined organic phases were washed with brine (100 mL), dried over Na_2SO_4, filtered, and the solvent removed *in vacuo*. The crude product was separated by column chromatography, and resulting blue solid was further purified by recrystallization as noted in the individual procedures, providing pentacenes **3a–i** as deep blue solids.

General Procedure B. Suzuki coupling to form pentacenes 3j and 3k.

To a solution of pentacene **3g** (1 equiv, ca. 0.1 mmol scale), the corresponding boronic acid (1.5 equiv) and $Pd(PPh_3)_2Cl_2$ (5 mol%) in dry, deoxygenated THF (20 mL) was added aqueous Na_2CO_3 (2 equiv dissolved in 5 mL H_2O). The resulting dark solution was heated at 80 °C for 2–4 h. After cooling the mixture to rt, the solvent was removed and the resulting blue product purified column chromatography and then recrystallization as noted in the individual procedures, providing pentacenes **3j** and **3k** as deep blue solids.

†The Grignard reagent used for the formation of **3i** is available commercially.

Synthetic Procedures and Spectral Characterization of New Compounds

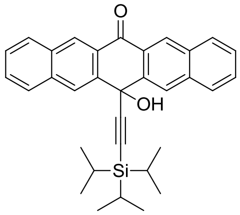

Ketone 4a. Compound was synthesized as previously reported.[1]

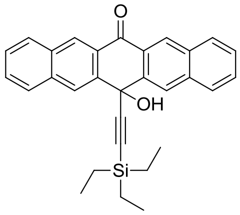

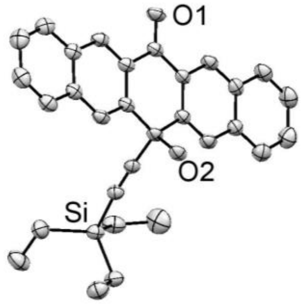

Ketone 4b. To a solution of triethylsilylacetylene (0.60 mL, 0.47 g, 3.3 mmol) in THF (7 mL) cooled to –78 °C was added dropwise HexLi (2.3 M in hexanes, 1.1 mL, 2.5 mmol). The solution was stirred for 10 min before being transferred slowly via cannula into a suspension of 6,13-pentacenequinone (0.997 g, 3.23 mmol) in THF (15 mL) at rt. The reaction mixture was stirred for 18 h at rt. The reaction mixture was cooled to –15 °C and quenched via the addition of satd. aq. NH_4Cl (1 mL). The suspension was filtered and the solid was washed with THF/H_2O (1:1, 3 x 4 mL), and then THF (3 x 4 mL). This allowed for the recovery of the excess 6,13-pentacenequinone, which could be used in subsequent reactions after drying under high vacuum (ca. 1 mmHg). The filtrate was collected into a filter flask which already contained satd. aq. NH_4Cl (50 mL), and after filtration and mixing, H_2O (50 mL) was added and the mixture was extracted with CH_2Cl_2 (60 mL, 30 mL). The combined organic phase was washed with satd. aq. NaCl (250 mL), dried ($MgSO_4$), filtered, and the solvent removed *in vacuo* to provide a solid orange residue. This solid was redissolved in minimal hot CH_2Cl_2 (12 mL) and precipitated by adding hexanes (50 mL) and cooling to –78 °C. The yellow colored solid was collected by vacuum filtration and washed with cold hexanes (4 x 6 mL). The solid was redissolved in acetone and filtered to remove insoluble impurities. The solvent of the filtrate was removed *in vacuo* to afford **4b** (1.02 g, 90%) as a yellow solid.

Mp = 205–208 °C (change in crystallinity observed visibly at 199 °C); R_f = 0.23 (CH_2Cl_2); UV–vis (CH_2Cl_2) λ_{max} (ε): 268 (40 900), 279 (43 800), 323 (28 600), 362 (6 010) nm; IR ($CHCl_3$ cast film):

3365 (s, br), 3053 (w), 2953 (m), 2910 (w), 2874 (m), 2167 (w), 1653 (s), 1281 (s) cm^{-1}; ^{1}H NMR (500 MHz, $CDCl_3$): δ 8.66 (s, 2H), 8.64 (s, 2H), 7.89 (app t, *J* = 8.9 Hz, 4H), 7.58 (app t, *J* = 7.5 Hz, 2H), 7.49 (app t, *J* = 7.4 Hz, 2H), 3.54 (s, 1H), 1.04 (t, *J* = 7.9 Hz, 9H), 0.68 (q, *J* = 7.9 Hz, 6H). ^{13}C NMR (100 MHz, $CDCl_3$): δ 184.3, 138.8, 135.7, 132.7, 129.8, 129.7, 128.9, 128.2, 128.2, 127.3, 127.2, 107.8, 90.7, 68.3, 7.5, 4.3. ^{13}C NMR (APT, 100 MHz, $CDCl_3$): δ 184.3 (C), 138.8 (C), 135.7 (C), 132.7 (C), 129.8 (CH), 129.6 (CH), 128.9 (CH), 128.3 (C), 128.2 (CH), 127.3 (CH), 127.3 (CH), 107.7 (C), 90.8 (C), 68.4 (C), 7.5 (CH_3), 4.3 (CH_2); ESI MS *m/z* 471.2 ($[M + Na]^+$, 100), 431.2 ($[M – OH]^+$, 56). ESI HRMS *m/z* calcd. for $C_{30}H_{28}O_2SiNa$ ($[M + Na]^+$) 471.1751, found 471.1749; TGA: *T*d ≈ 210 °C. DSC: *T*g = 200 °C, mp = 206 °C; decomposition, 260 °C (onset) and 285 °C (peak). Anal. calcd. for $C_{30}H_{28}O_2Si$: C, 80.32; H, 6.29. Found: C, 80.51; H, 6.27.

Crystals suitable for X-ray crystallography were obtain by slow evaporation of solutions of **4b** in CH_2Cl_2 left standing in the refrigerator at 4 °C for several days. X-ray crystallographic data for **4b**: $C_{30}H_{28}O_2Si$, *Fw* = 448.61; crystal dimensions 0.54 × 0.18 × 0.12 mm^{-3}; triclinic space group *P*-1; a = 8.2573(14) Å, b = 9.4816(15) Å, c = 16.088(3) Å; α = 94.039(2)°, β = 98.657(2)°, γ = 104.169(2)°; V = 1199.8(3) $Å^3$; Z = 2; $\rho_{(calcd.)}$ = 1.242 g cm^{-3}; μ = 0.123 mm^{-1}; λ = 0.71073 Å; T = –80 °C; $2\theta_{max}$ = 52.80°; total data collected = 9259; R_1 = 0.0553 [3510 observed reflections with $Fo^2 \geq 2\sigma(Fo^2)$]; wR_2 = 0.1463 for 299 variables and 4871 unique reflections; residual electron density = 0.448 and –0.176 e $Å^{-3}$. CCDC 985834.

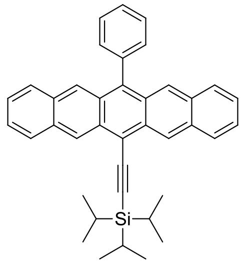

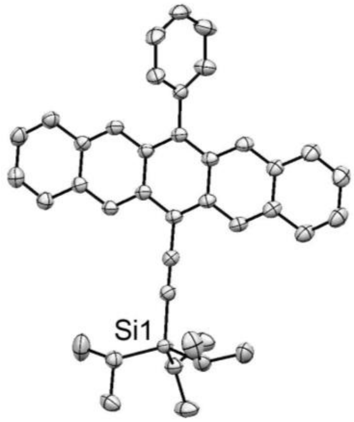

Pentacene 3a. According to General Procedure A, phenyllithium (1.8 M in dibutylether, 1.67 mL, 3.00 mmol) and ketone **4a** (491 mg, 1.00 mmol) in dry, deoxygenated THF (20 mL) were used, followed by reductive aromatization using $SnCl_2 \cdot 2H_2O$ (677 mg, 3.00 mmol) and 10% aqueous H_2SO_4 (1 mL) in THF (20 mL). Purification was achieved by column chromatography (silica gel, hexanes/CH_2Cl_2, 3:1) and final recrystallization from CH_2Cl_2 layered with MeOH. Pentacene **3a** was obtained as a dark blue solid (274 mg, 51%).

Mp = 162–165 °C; R_f = 0.30 (hexanes); UV-vis (CH_2Cl_2) λ_{max} (ε): 253 (18 000), 270 (21 000), 298 (105 000), 310 (240 000), 351 (7 300), 406 (1 300), 435 (1 420), 580 (9 000), 621 (12 300) nm. UV-vis (CH_2Cl_2 cast film) λ_{max}: 323, 350, 411, 436, 594, 637 nm; Fluorescence (CH_2Cl_2, λ_{exc} = 615 nm): $\lambda_{max,\ em}$ = 665 nm; IR (ATR): 3046 (w), 2937 (s), 2860 (s), 2130 (m), 1374 (s), 874 (s) cm^{-1}; ^1H NMR (300 MHz, $CDCl_3$): δ 9.36 (s, 2H), 8.26 (s, 2H), 7.95 (d, J = 8.5 Hz, 2H), 7.75–7.67 (m, 5H), 7.56–7.54 (m, 2H), 7.37–7.27 (m, 4H), 1.38–1.37 (m, 21H). ^{13}C NMR (100 MHz, $CDCl_3$): δ 139.0, 138.4, 131.9, 131.6, 131.4, 131.0, 128.7, 128.6, 128.5, 127.9, 126.3, 125.8, 125.7, 125.3, 117.3, 105.3, 104.9, 19.0, 11.7 (one signal coincident or not observed); ESI HRMS calcd. for $C_{39}H_{38}Si$ (M^+) m/z 534.27373, found 534.27406; TGA: T_d ~370 °C; DSC: Mp = 177 °C, decomposition: 178 °C (onset), 179 °C (peak).

A crystal suitable for X-ray crystallographic analysis has been grown by slowly evaporation of a CH_2Cl_2 solution at rt. X-ray data for **3a** ($C_{39}H_{38}Si$), F_w = 534.78; monoclinic crystal system; space group C2/c; a = 22.6564(6) Å, b = 23.1461(9) Å, c = 11.8317(3) Å; β = 104.470(2)°; V = 6007.8(3) $Å^3$; Z = 8; $\rho_{(calcd.)}$ = 1.183 g/cm^3; $2\theta_{max}$ = 54.96°; μ = 0.104 mm^{-1}; T = 173(2) K; total data collected = 13174; R_1 = 0.0488 [6875 observed reflections with $F_0^2 \geq 2\sigma(F_0^2)$]; ωR_2 = 0.1345 for 361 variables with $F_0^2 \geq -3\sigma(F_0^2)$; residual electron density = 0.208 and –0.247 e $Å^{-3}$. CCDC 985357.

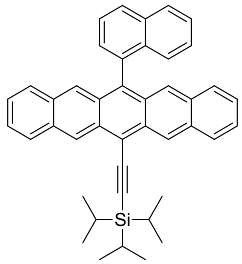

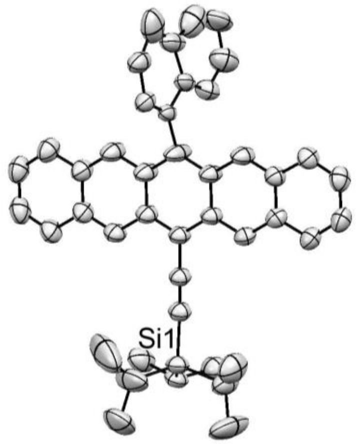

Pentacene 3b. According to General Procedure A, 1-bromonaphthalene (627 mg, 3.00 mmol), *n*BuLi (2.5 M in hexanes, 1.16 mL, 2.90 mmol), and ketone **4a** (491 mg, 1.00 mmol) in dry, deoxygenated THF (20 mL) were used, followed by reductive aromatization using $SnCl_2$•$2H_2O$ (677 mg, 3.00 mmol) and 10% aqueous H_2SO_4 (1 mL) in THF (20 mL). Purification was achieved by column chromatography (silica gel, hexanes/CH_2Cl_2, 5:1) and final recrystallization from CH_2Cl_2 layered with MeOH. Pentacene **3b** was obtained as a dark blue-purple solid (142 mg, 25%).

Mp = 244–246 °C; R_f = 0.55 (hexanes/CH_2Cl_2 5:1); UV-vis (CH_2Cl_2) λ_{max} (ε): 270 (19 500), 298 (sh, 85 800), 309 (250 000), 348 (6 000), 435 (1 350), 576 (7 700), 621 (11 500) nm. UV-vis (CH_2Cl_2 cast film) λ_{max}: 323, 340, 412, 438, 544, 593, 635 nm; Fluorescence (CH_2Cl_2, λ_{exc} = 615 nm): $\lambda_{max, em}$ = 656 nm; IR (ATR): 3044 (w), 2938 (s), 2859 (s), 2133 (s), 1459 (s), 1357 (s), 727 (s) cm^{-1}; ^{1}H NMR (300 MHz, $CDCl_3$): δ 9.39 (s, 2H), 8.16 (d, *J* = 4.2 Hz, 1H), 8.06 (d, *J* = 4.1 Hz, 1H), 8.00 (s, 2H), 7.94 (d, *J* = 8.2 Hz, 2H), 7.78 (t, *J* = 7.1 Hz, 1H), 7.59 (d, *J* = 8.6 Hz, 1H), 7.55 (d, *J* = 8.6 Hz, 2H), 7.46 (t, *J* = 7.6 Hz, 1H), 7.31 (t, *J* = 8.3 Hz, 2H), 7.21–7.16 (m, 2H), 7.10 (t, *J* = 8.3 Hz, 1H), 7.00 (d, J = 8.6 Hz, 1H), 1.47–1.38 (m, 21H). ^{13}C NMR (100 MHz, $CDCl_3$): δ 136.7, 136.5, 133.8, 133.7, 132.0, 131.5, 131.0, 129.5, 129.1, 128.6, 128.5, 128.3, 126.7, 126.4, 126.3, 126.1, 125.90, 125.85, 125.80, 125.7, 125.3, 117.6, 105.5, 104.8, 19.0, 11.7; ESI HRMS calcd. $C_{43}H_{40}Si$ (M^+) *m/z* 584.28938, found 584.28893; Element. Anal. calcd. for $C_{43}H_{40}Si$: C, 88.30; H, 6.89. Found: C, 87.94; H, 6.91. TGA: T_d ~ 370 °C; DSC: Mp = 248 °C.

A crystal suitable for X-ray crystallographic analysis has been grown by slowly evaporation of a CH_2Cl_2 solution layered with acetone at 4 °C. X-ray data for **3b** ($C_{43}H_{40}Si$), F_w = 584.84; triclinic crystal system; space group *P*–1; *a* = 11.1091(5) Å, *b* = 11.6246(6) Å, *c* = 13.6377(6) Å; α = 93.926(4)°, β = 100.072(4)°, γ = 103.336(4)°; *V* = 1676.34(14) Å^3; *Z* = 2; $\rho_{(calcd.)}$ = 1.159 g/cm^3; $2\theta_{max}$ = 146.56°; μ = 0.819 mm^{-1}; *T* = 172.9 K; total data collected = 25726; R_1 = 0.0719 [6655

observed reflections with $F_0^2 \geq 2\sigma(F_0^2)$]; $\omega R_2 = 0.2454$ for 449 variables with $F_0^2 \geq -3\sigma(F_0^2)$ and 0 restraints; residual electron density = 0.622 and –0.496 e Å^{-3}. The naphthyl group showed disorder, which has been resolved and refined to the following occupation factors: 55:45%. CCDC 985358.

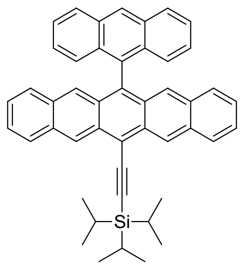

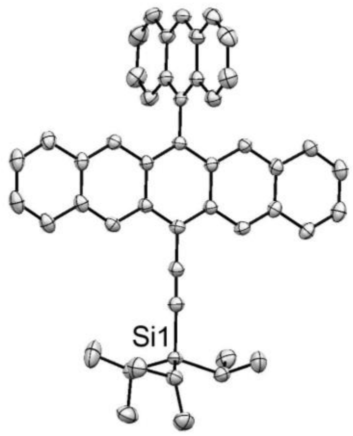

Pentacene 3c. This compound was synthesized as previously reported.[2]

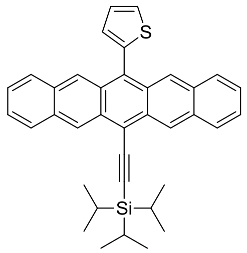

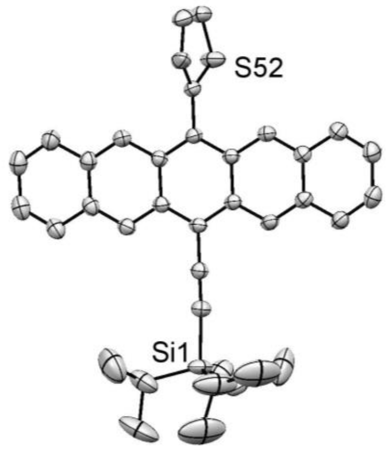

Pentacene 3d. According to General Procedure A, 2-bromothiophene (489 mg, 3.00 mmol), *n*BuLi (2.5 M in hexanes, 1.16 mL, 2.90 mmol), and ketone **4a** (491 mg, 1.00 mmol) in dry, deoxygenated THF (20 mL) were used, followed by reductive aromatization using $SnCl_2$•$2H_2O$ (677 mg, 3.00 mmol) and 10% aqueous H_2SO_4 (1 mL) in THF (20 mL). Purification was achieved by column chromatography (silica gel, hexanes/CH_2Cl_2, 3:1) and final recrystallization from CH_2Cl_2 layered with MeOH. Pentacene **3d** was obtained as a dark blue solid (116 mg, 21%).

Mp = 316–318 °C (color change); R_f = 0.70 (hexanes/CH_2Cl_2, 3:1); UV-vis (CH_2Cl_2) λ_{max} (ε): 269 (13 200), 297 (sh, 83 600), 309 (220 000), 351 (7 400), 436 (2 000), 535 (3 500), 576 (8 400), 623 (13 200) nm. UV-vis (CH_2Cl_2 cast film) λ_{max}: 327, 350, 413, 437, 545, 589, 637 nm; Fluorescence (CH_2Cl_2, λ_{exc} = 615 nm): $\lambda_{max, em}$ = 655 nm; IR (ATR): 3043 (w), 2936 (s), 2859 (s),

2125 (m), 1459 (m), 1363 (m), 870 (s), 727 (s) cm^{-1}; ^1H NMR (300 MHz, $CDCl_3$): δ 9.35 (s, 2H), 8.48 (s, 2H), 7.96 (d, *J* = 8.4 Hz, 2H), 7.82 (d, *J* = 8.4 Hz, 2H), 7.74 (dd, *J* = 4.0 Hz, 1.1 Hz, 1H), 7.44–7.42 (m, 1H), 7.38–7.27 (m, 5H), 1.41–1.38 (m, 21H). ^{13}C NMR (75 MHz, $CDCl_3$): δ 139.0, 131.8, 131.6, 130.6, 130.0, 129.78, 129.76, 128.6, 128.4, 127.3, 127.1, 125.9, 125.7, 125.5, 118.6, 106.0, 104.6, 18.9, 11.6 (one signal coincident or not observed); ESI HRMS calcd. $C_{37}H_{36}SSi$ (M^+) *m*/*z* 540.23015, found 540.23092; Element. Anal. calcd. for $C_{37}H_{36}SSi$: C, 82.17; H, 6.71; S, 5.93. Found: C, 81.65; H, 6.98; S, 5.79; TGA: T_d ~ 372 °C; DSC: decomposition, 206 °C (onset), 247 °C (peak).

A crystal suitable for X-ray crystallographic analysis has been grown by slowly evaporation of a CH_2Cl_2 solution layered with acetone at 4 °C. X-ray data for **3d** ($C_{37}H_{36}SiS$), F_w = 540.81; monoclinic crystal system; space group $P2_1/n$; a = 13.4483(3) Å, b = 13.6265(4) Å, c = 16.7260(4) Å; β = 91.359(2)°; V = 3064.22(13) $Å^3$; Z = 4; $\rho_{(calcd.)}$ = 1.172 g/cm^3; $2\theta_{max}$ = 146.8°; μ = 1.474 mm^{-1}; T = 173.00(10) K; total data collected = 10141; R_1 = 0.0641 [5909 observed reflections with $F_0^2 \geq 2\sigma(F_0^2)$]; ωR_2 = 0.1896 for 368 variables with $F_0^2 \geq -3\sigma(F_0^2)$ and 10 restraints; residual electron density = 0.54 and –0.48 e $Å^{-3}$. The disorder in the thiophene unit was refined using the following occupancies: S52/C55 : S52a/C55a = 66:34%, C4/C5 : C4a/C5a = 60:40%. CCDC 985362.

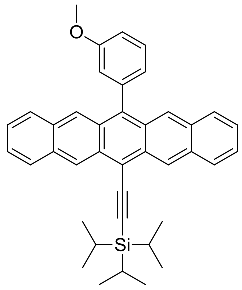

Pentacene 3e. According to General Procedure A, 3-bromoanisole (561 mg, 3.00 mmol), *n*BuLi (2.5 M in hexanes, 1.16 mL, 2.90 mmol), and ketone **4a** (491 mg, 1.00 mmol) in dry, deoxygenated THF (20 mL) were used, followed by reductive aromatization using $SnCl_2 \cdot 2H_2O$ (677 mg, 3.00 mmol) and 10% aqueous H_2SO_4 (1 mL) in THF (20 mL). Purification was achieved by column chromatography (silica gel, hexanes/CH_2Cl_2, 3:1) and final recrystallization from CH_2Cl_2 layered with MeOH. Pentacene **3e** was obtained as a dark blue solid (340 mg, 60%).

Mp = 210–213 °C; R_f = 0.55 (hexanes/CH_2Cl_2 3:1); UV-vis (CH_2Cl_2) λ_{max} (ε): 270 (19 700), 297 (92 300), 309 (281 000), 351 (6 550), 435 (1 270), 580 (8 260), 621 (11 370) nm. UV-vis (CH_2Cl_2 cast film) λ_{max}: 274, 321, 411, 436, 540, 588, 634 nm; Fluorescence (CH_2Cl_2, λ_{exc} = 615 nm): $\lambda_{max, em}$ = 663 nm; IR (ATR): 3043 (w), 2938 (s), 2861 (s), 2128 (m), 1588 (s), 1252 (s), 877 (s),

724 (s) cm^{-1}; ^{1}H NMR (400 MHz, $CDCl_3$): δ 9.37 (s, 2H), 8.32 (s, 2H), 7.97 (d, *J* = 8.8 Hz, 2H), 7.77 (d, *J* = 8.8 Hz, 2H), 7.61 (t, *J* = 7.6 Hz, 1H), 7.36 (t, *J* = 8.5 Hz, 1H), 7.30–7.27 (m, 1H), 7.30–7.27 (m, 2H), 7.21 (ddd, *J* = 8.4 Hz, 2.4 Hz, 0.8 Hz, 1H), 7.16 (dt, *J* = *J* = 7.6 Hz, 1.2 Hz, 1H), 7.12–7.11 (m, 1H), 3.90 (s, 3H), 1.42–1.37 (m, 21H). ^{13}C NMR (100 MHz, $CDCl_3$): δ 159.7, 140.4, 138.1, 131.9, 131.4, 130.9, 129.6, 128.7, 128.5, 128.3, 126.3, 125.9, 125.7, 125.4, 124.1, 117.3, 116.9, 113.6, 105.3, 104.9, 55.4, 19.0, 11.7; ESI HRMS calcd. $C_{40}H_{40}OSi$ (M^+) *m/z* 564.28429, found 564.28416.

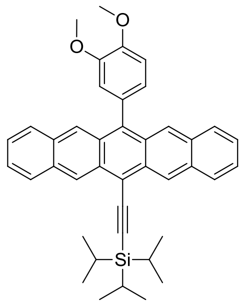

Pentacene 3f. According to General Procedure A, 4-bromoveratrole (651 mg, 3.00 mmol), *n*BuLi (2.5 M in hexanes, 1.16 mL, 2.90 mmol), and ketone **4a** (491 mg, 1.00 mmol) in dry, deoxygenated THF (20 mL) were used, followed by reductive aromatization using $SnCl_2$•$2H_2O$ (677 mg, 3.00 mmol) and 10% aqueous H_2SO_4 (1 mL) in THF (20 mL). Purification was achieved by column chromatography (silica gel, CH_2Cl_2/hexanes, 2:1) and final recrystallization from CH_2Cl_2 layered with MeOH. Pentacene **3f** was obtained as a dark blue solid (345 mg, 58%).

Mp = 255–257 °C; R_f = 0.64 (CH_2Cl_2/hexanes 1:1); UV-vis (CH_2Cl_2) λ_{max} (ε): 270 (20 000), 298 (86 300), 310 (233 000), 352 (6 050), 435 (1 260), 583 (7 280), 622 (9 370) nm. UV-vis (CH_2Cl_2 cast film) λ_{max}: 321, 412, 437, 525, 565, 618, 655 nm; Fluorescence (CH_2Cl_2, λ_{exc} = 615 nm): $\lambda_{max, em}$ = 671 nm; IR (ATR): 3040 (w), 2937 (s), 2861 (s), 2125 (s), 1511 (s), 1457 (s), 1237 (s), 876 (s), 737 (s) cm^{-1}; ^{1}H NMR (400 MHz, $CDCl_3$): δ 9.36 (s, 2H), 8.35 (s, 2H), 7.96 (d, *J* = 8.8 Hz, 2H), 7.78 (d, *J* = 8.4 Hz, 2H), 7.36 (t, *J* = 7.2 Hz, 2H), 7.31–7.27 (m, 2H), 7.18–7.16 (m, 1H), 7.12–7.09 (m, 1H), 7.07–7.06 (m, 1H), 4.10 (s, 3H), 3.88 (s, 3H), 1.41–1.36 (m, 21H). ^{13}C NMR (100 MHz, $CDCl_3$): δ 148.9, 148.7, 138.2, 131.9, 131.42, 131.37, 131.0, 128.71, 128.66, 128.5, 126.3, 125.9, 125.7, 125.3, 123.9, 117.1, 114.6, 111.3, 105.2, 104.9, 56.02, 55.99, 19.0, 11.7; ESI HRMS calcd. $C_{41}H_{42}O_2Si$ (M^+) *m/z* 594.29486, found 594.29304, $C_{41}H_{42}NaO_2Si$ ($[M + Na]^+$) *m/z* 617.28463, found 617.28264.

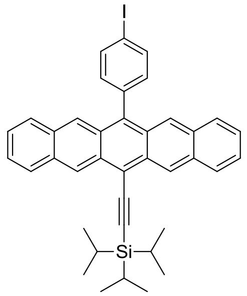

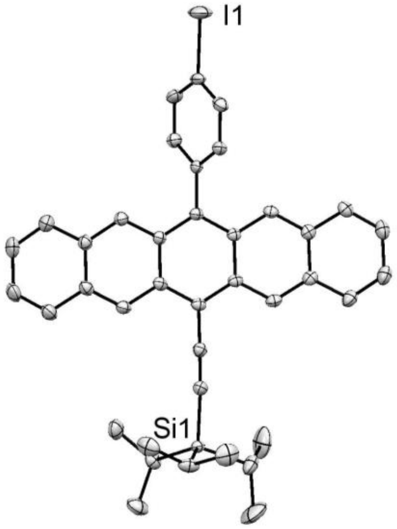

Pentacene 3g. According to General Procedure A, 1,4-diiodobenzene (990 mg, 3.00 mmol), *n*BuLi (2.5 M in hexanes, 1.16 mL, 2.90 mmol), and ketone **4a** (491 mg, 1.00 mmol) in dry, deoxygenated THF (20 mL) were used, followed by reductive aromatization using $SnCl_2$•$2H_2O$ (677 mg, 3.00 mmol) and 10% aqueous H_2SO_4 (1 mL) in THF (20 mL). Purification was achieved by column chromatography (silica gel, hexanes/CH_2Cl_2, 4:1) and final recrystallization from CH_2Cl_2 layered with MeOH. Pentacene **3g** was obtained as a dark blue solid (294 mg, 45%).

Mp = 266–268 °C; R_f = 0.76 (hexanes/CH_2Cl_2 3:1); UV-vis (CH_2Cl_2) λ_{max} (ε): 269 (20 000), 309 (270 000), 351 (7 500), 435 (1 600), 576 (9 600), 621 (14 000) nm. UV-vis (CH_2Cl_2 cast film) λ_{max}: 274, 318, 409, 437, 489, 534, 628 nm; Fluorescence (CH_2Cl_2, λ_{exc} = 615 nm): $\lambda_{max, em}$ = 658 nm; IR (ATR): 3041 (w), 2939 (s), 2858 (s), 2136 (m), 1460 (s), 1375 (s), 873 (s), 727 (s) cm^{-1}; ^1H NMR (300 MHz, $CDCl_3$): δ 9.35 (s, 2H), 8.22 (s, 2H), 8.02 (d, J = 8.1 Hz, 2H), 7.94 (d, J = 8.5 Hz, 2H), 7.75 (d, J = 8.5 Hz, 2H), 7.38–7.27 (m, 6H), 1.48–1.30 (m, 21H). ^{13}C NMR (75 MHz, $CDCl_3$): δ 138.4, 137.7, 133.4, 131.8, 131.4, 130.7, 128.43, 128.38, 128.1, 125.82, 125.75, 125.6, 125.4, 117.6, 105.5, 104.6, 93.7, 18.9, 11.5 (one signal coincident or not observed); ESI HRMS calcd. $C_{39}H_{37}ISi$ (M^+) *m*/*z* 660.17037, found 660.16903.

A crystal suitable for X-ray crystallographic analysis has been grown by slowly evaporation of a CH_2Cl_2 solution layered with acetone at 4 °C. X-ray data for **3g** ($C_{39}H_{37}SiI$), F_w = 660.68; monoclinic crystal system; space group $P2_1/n$; a = 17.0739(3) Å, b = 9.5155(2) Å, c = 19.8065(4) Å; β = 90.845(2)°; V = 3217.54(11) Å^3; Z = 4; $\rho_{(calcd.)}$ = 1.364 g/cm^3; $2\theta_{max}$ = 58.56°; μ = 1.058 mm^{-1}; T = 146.9 K; total data collected = 13795; R_1 = 0.0355 [7300 observed reflections with $F_0^2 \geq 2\sigma(F_0^2)$]; ωR_2 = 0.0834 for 376 variables with $F_0^2 \geq -3\sigma(F_0^2)$; residual electron density = 0.885 and –1.237 e Å^{-3}. CCDC 985359.

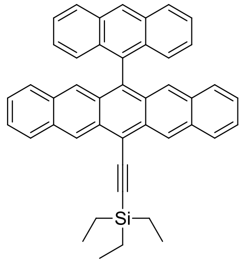

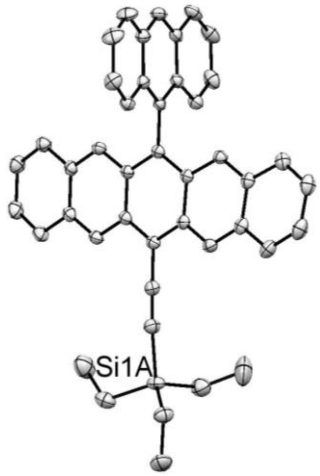

Pentacene 3h. According to General Procedure A, 9-bromoanthracene (517 mg, 2.01 mmol), *n*BuLi (2.5 M in hexanes, 0.78 mL, 1.94 mmol), and ketone **4b** (300 mg, 0.67 mmol) in dry, deoxygenated THF (20 mL) were used, followed by reductive aromatization using $SnCl_2{\bullet}2H_2O$ (454 mg, 2.01 mmol) and 10% aqueous H_2SO_4 (1 mL) in THF (20 mL). Purification was achieved by column chromatography (silica gel, hexanes/CH_2Cl_2, 4:1) and final recrystallization from CH_2Cl_2 layered with MeOH. Pentacene **3h** was obtained as a dark blue solid (30 mg, 8%).

Mp = 291–293 °C; R_f = 0.47 (hexanes/CH_2Cl_2 4:1); UV-vis (CH_2Cl_2) λ_{max} (ε): 256 (146 000), 298 (100 000), 310 (315 000), 349 (13 000), 366 (13 000), 387 (11 000), 436 (3 300), 536 (5 500), 576 (12 500), 622 (17 000) nm. UV-vis (CH_2Cl_2 cast film) λ_{max}: 260, 324, 349, 369, 390, 437, 503, 545, 592, 638 nm; Fluorescence (CH_2Cl_2, λ_{exc} = 615 nm): $\lambda_{max,\ em}$ = 652 nm; IR (ATR): 3045 (w), 2949 (m), 2878 (s), 2124 (m), 1449 (m), 1323 (m), 875 (s), 724 (s) cm^{-1}; ^1H NMR (300 MHz, $CDCl_3$): δ 9.43 (s, 2H), 8.79 (s, 1H), 8.21 (d, *J* = 8.6 Hz, 2H), 7.99 (d, *J* = 8.6 Hz, 2H), 7.72 (s, 2H), 7.47–7.42 (m, 2H), 7.38 (d, *J* = 8.7 Hz, 2H), 7.30 (t, *J* = 8.5 Hz, 2H), 7.15–7.02 (m, 6H), 1.36 (t, *J* = 7.9 Hz, 9H), 1.05–0.97 (q, *J* = 7.9 Hz, 6H). ^{13}C NMR (75 MHz, $CDCl_3$): δ 135.1, 133.2, 132.1, 131.8, 131.7, 131.6, 131.0, 129.6, 128.63, 128.56, 128.46, 127.6, 127.0, 126.2, 126.02, 125.95, 125.90, 125.4, 125.3, 117.7, 106.8, 104.2, 8.0, 4.9; ESI HRMS calcd. $C_{44}H_{36}Si$ (M^+) *m/z* 592.25808, found 592.25838; TGA: T_d ~410 °C; DSC: Mp = 287 °C, decomposition: 288 °C (onset), 290 °C (peak).

A crystal suitable for X-ray crystallographic analysis has been grown by slowly evaporation of a CH_2Cl_2 solution layered with acetone at 4 °C. X-ray data for **3h** ($C_{44}H_{36}Si$), F_w = 592.82; monoclinic crystal system; space group $P2_1/c$; a = 16.1928(4) Å, b = 8.8734(2) Å, c = 23.4480(8) Å; β = 107.485(3)°; V = 3213.45(16) Å^3; Z = 4; $\rho_{(calcd.)}$ = 1.225 g/cm^3; $2\theta_{max}$ = 121.08°; μ = 0.865 mm^{-1}; T = 153.00(10) K; total data collected = 7274; R_1 = 0.0407 [4679 observed reflections with

$F_0^2 \geq 2\sigma(F_0^2)$]; ωR_2 = 0.1133 for 409 variables with $F_0^2 \geq -3\sigma(F_0^2)$; residual electron density = 0.43 and –0.29 e Å^{-3}. CCDC 985363.

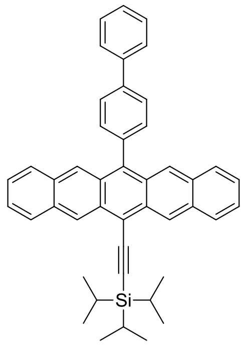

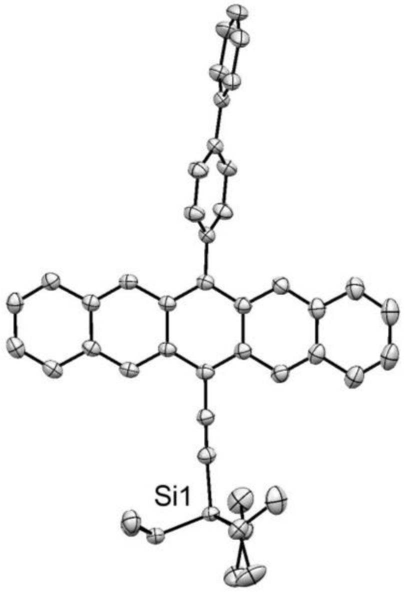

Pentacene 3i. According to General Procedure A, 4-biphenylmagnesiumbromide (0.5 M in THF, 12 mL, 6.0 mmol) and ketone **4a** (981 mg, 2.00 mmol) in dry, deoxygenated THF (20 mL) were used, followed by reductive aromatization using $SnCl_2$•$2H_2O$ (1.35 g, 6.00 mmol) and 10% aqueous H_2SO_4 (1 mL) in THF (20 mL). Purification was achieved by column chromatography (silica gel, hexanes/CH_2Cl_2, 9:1) and final recrystallization from CH_2Cl_2 layered with MeOH. Pentacene **3i** was obtained as a dark blue solid (300 mg, 25%).

Mp = 211–214 °C; R_f = 0.20 (hexanes); UV-vis (CH_2Cl_2) λ_{max} (ε): 267 (27 600), 299 (68 900), 310 (208 000), 351 (6 400), 435 (1 000), 580 (5 900), 623 (8 000) nm. UV-vis (CH_2Cl_2 cast film) λ_{max}: 271, 324, 410, 437, 598, 641 nm; Fluorescence (CH_2Cl_2, λ_{exc} = 615 nm): $\lambda_{max,\ em}$ = 665 nm; IR (ATR): 2935 (m), 2133 (w), 1593 (s), 1166 (s), 689 (s) cm^{-1}; ^{1}H NMR (300 MHz, $CDCl_3$): δ 9.38 (s, 2H), 8.36 (s, 2H), 7.98–7.90 (m, 4H), 7.84 (d, *J* = 7.5 Hz, 2H), 7.76 (d, *J* = 8.5 Hz, 2H), 7.63–7.25 (m, 9H), 1.40–1.39 (m, 21H). ^{13}C NMR (75 MHz, $CDCl_3$): δ 140.7, 140.5, 137.96, 137.91, 132.0, 131.9, 131.4, 130.9, 128.9, 128.6, 128.5, 128.4, 127.5, 127.2, 127.1, 126.2, 125.8, 125.7, 125.3, 117.3, 105.3, 104.8, 19.0, 11.6; MS LDI *m*/*z* 610 (M$^+$). APPI HRMS calcd. $C_{45}H_{43}Si$ ([M + H]$^+$) *m*/*z* 611.31285, found 611.31422; TGA: T_d ~ 380 °C; DSC: Mp = 225 °C.

A crystal suitable for X-ray crystallographic analysis has been grown by slowly evaporation of a THF solution layered with MeOH at 4 °C. X-ray data for **3i** ($C_{45}H_{42}Si$), F_w = 610.88; triclinic crystal system; space group *P*–1; *a* = 8.8430(7) Å, *b* = 13.1980(9) Å, *c* = 15.6961(10) Å; α = 75.899(6)°, β = 80.962(6)°, γ = 78.579(6)°; *V* = 1730.1(2) Å^3; *Z* = 2; $\rho_{(calcd.)}$ = 1.173 g/cm^3; $2\theta_{max}$ = 141.52°; μ

= 0.814 mm^{-1}; T = 173 K; total data collected = 10087; R_1 = 0.0440 [6284 observed reflections with $F_0^2 \geq 2\sigma(F_0^2)$]; ωR_2 = 0.1253 for 421 variables with $F_0^2 \geq -3\sigma(F_0^2)$; residual electron density = 0.342 and –0.285 e $Å^{-3}$. CCDC 985361.

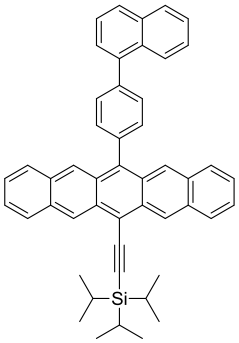

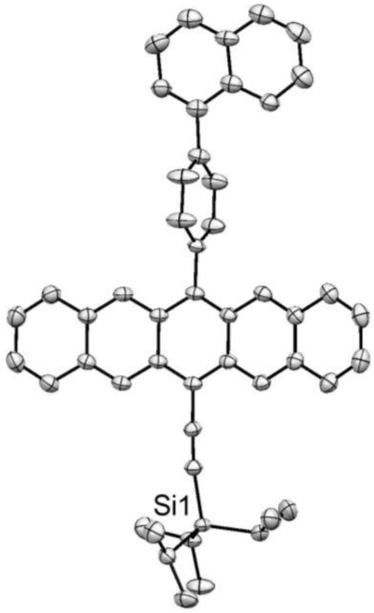

Pentacene 3j. Iodophenyl-pentacene **3g** (70 mg, 0.11 mmol), 1-naphthylboronic acid (29 mg, 0.16 mmol), $Pd(PPh_3)_2Cl_2$ (4 mg, 5 μmol), Na_2CO_3 (23 mg, 0.21 mmol) were used according to General Procedure B. The reaction mixture was plugged through a pad of silica gel, eluting with CH_2Cl_2/hexanes (1:1) and the blue band was collected. The solvent was removed and the mixture was further recrystallized from CH_2Cl_2 layered with MeOH at –15 °C to afford pentacene **3j** as a deep blue solid (67 mg, 92%).

Mp = 233–235 °C; R_f = 0.30 (hexanes/CH_2Cl_2 1:3); UV-vis (CH_2Cl_2) λ_{max} (ε): 258 (41 000), 300 (sh, 105 000), 309 (300 000), 352 (10 500), 386 (2 360), 435 (1 250), 580 (10 500), 623 (13 700) nm. UV-vis (CH_2Cl_2 cast film) λ_{max}: 259, 320, 355, 410, 437, 589, 633 nm; Fluorescence (CH_2Cl_2, λ_{exc} = 615 nm): $\lambda_{max, em}$ = 665 nm; IR (ATR): 3041 (w), 2938 (s), 2860 (s), 2130 (m), 1460 (s), 1374 (s), 874 (s), 732 (s) cm^{-1}; ^1H NMR (300 MHz, $CDCl_3$): δ 9.38 (s, 2H), 8.42 (s, 2H), 8.27–8.24 (m, 1H), 7.99–7.93 (m, 4H), 7.82 (d, J = 7.9 Hz, 4H), 7.72–7.63 (m, 4H), 7.61–7.57 (m, 2H), 7.38–7.27 (m, 4H), 1.40–137 (m, 21H). ^{13}C NMR (75 MHz, $CDCl_3$): δ 140.0, 139.7, 137.8, 137.6, 133.7, 131.7, 131.4, 131.3, 131.2, 130.7, 130.0, 128.5, 128.31, 128.25, 127.7, 127.1, 126.0, 125.8, 125.7, 125.6, 125.3, 125.2, 117.1, 105.1, 104.6, 18.8, 11.5 (three signals coincident or not observed); ESI HRMS calcd. for $C_{49}H_{44}Si$ (M^+) m/z 660.32123, found 660.32065; TGA: T_d ~ 380 °C; DSC: Mp = 220 °C.

A crystal suitable for X-ray crystallographic analysis has been grown by slowly evaporation of a CH_2Cl_2 solution layered with MeOH at 4 °C. X-ray data for **3j** ($C_{49}H_{44}Si$), F_w = 660.93; triclinic crystal system; space group P–1 (No. 2); a = 8.7000(6) Å, b = 13.7195(9) Å, c = 16.1219(11) Å; α = 102.755(6)°; β = 100.911(6)°; γ = 94.442(5)°; V = 1828.6(2) Å^3; Z = 2; $\rho_{(calcd.)}$ = 1.200 g/cm^3; $2\theta_{max}$ = 147.24°; μ = 0.810 mm^{-1}; T = 173 K; total data collected = 13530; R_1 = 0.0527 [7123 observed reflections with $F_0^2 \geq 2\sigma(F_0^2)$]; ωR_2 = 0.1556 for 457 variables with $F_0^2 \geq -3\sigma(F_0^2)$; residual electron density = 0.771 and –0.312 e Å^{-3}. CCDC 985360.

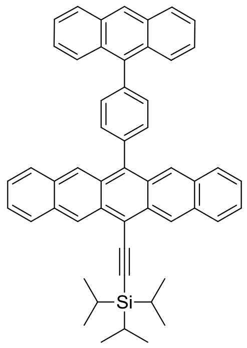

Pentacene 3k. Iodophenyl-pentacene **3g** (80 mg, 0.12 mmol), 9-anthracenylboronic acid (40 mg, 0.18 mmol), $Pd(PPh_3)_2Cl_2$ (4 mg, 6 μmmol), Na_2CO_3 (26 mg, 0.24 mmol) were used according to General Procedure B. The reaction mixture was plugged through a pad of neutral Al_2O_3, eluted with hexanes/CH_2Cl_2 (3:1), and the blue band was collected. The solvent was removed and the product was finally purified by recrystallization from MeOH/acetone (40 mL; 1:1) at –15 °C. Pentacene **3k** was obtained as a deep blue solid (58 mg, 68%).

Mp = 284–286 °C; R_f = 0.5 (Al_2O_3, hexanes/CH_2Cl_2 1:3); UV-vis (CH_2Cl_2) λ_{max} (ε): 258 (132 000), 310 (230 000), 350 (12 000), 367 (11 500), 387 (9 700), 435 (1 380), 580 (8 500), 623 (11 700) nm. UV-vis (CH_2Cl_2 cast film) λ_{max}: 260, 321, 370, 389, 436, 593, 635 nm; Fluorescence (CH_2Cl_2, λ_{exc} = 615 nm): $\lambda_{max, em}$ = 665 nm; IR (ATR): 3047 (w), 2937 (s), 2859 (s), 2129 (m),1457 (s), 1375 (s), 877 (s), 726 (s) cm^{-1}; ^{1}H NMR (300 MHz, $CDCl_3$): δ 9.41 (s, 2H), 8.59 (s, 1H), 8.52 (s, 2H), 8.15–8.11 (m, 2H), 8.04–7.98 (m, 4H), 7.88 (d, J = 8.4 Hz, 2H), 7.77 (s, 4H), 7.57–7.52 (m, 4H), 7.42–7.32 (m, 4H), 1.40–1.39 (m, 21H). ^{13}C NMR (100 MHz, $CDCl_3$): δ 138.4, 138.14, 138.08, 136.7, 132.0, 131.7, 131.6, 131.53, 131.52, 131.1, 130.3, 128.8, 128.62, 128.61, 128.56, 126.9, 126.8, 126.3, 126.0, 125.68, 125.67, 125.5, 125.2, 117.5, 105.5, 104.9, 19.0, 11.7; MS ESI (THF) m/z 710 (M^+). APPI HRMS calcd. for $C_{53}H_{47}Si$ ($[M + H]^+$) m/z 711.34415, found 711.34439.

Optical Properties of Pentacenes 3a–k

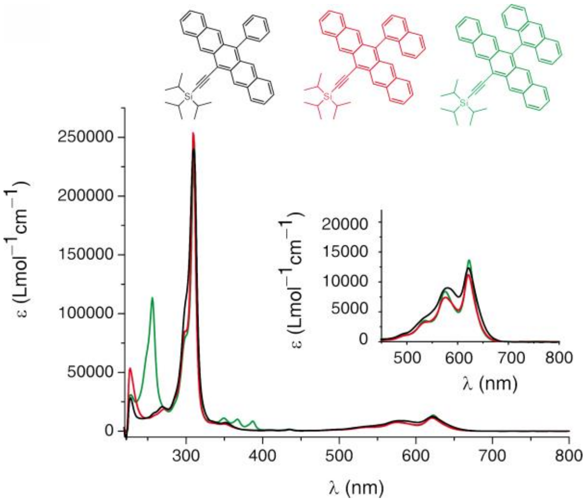

Figure S1: UV–vis spectra of pentacenes **3a**–**c** in CH_2Cl_2.

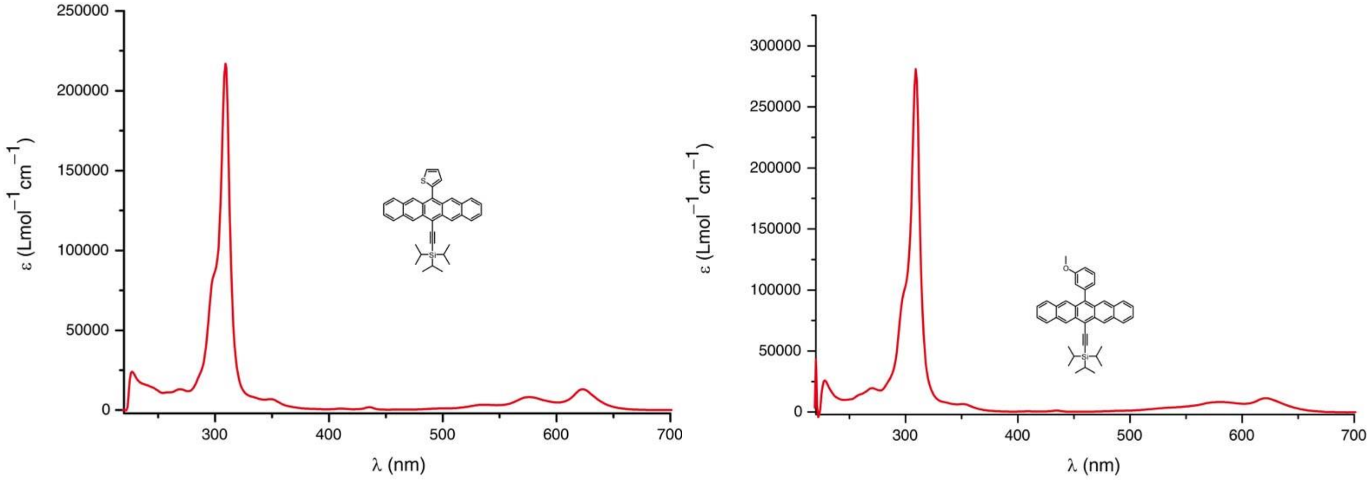

Figure S2: UV–vis spectra of pentacenes **3d** (left) and **3e** (right) in CH_2Cl_2.

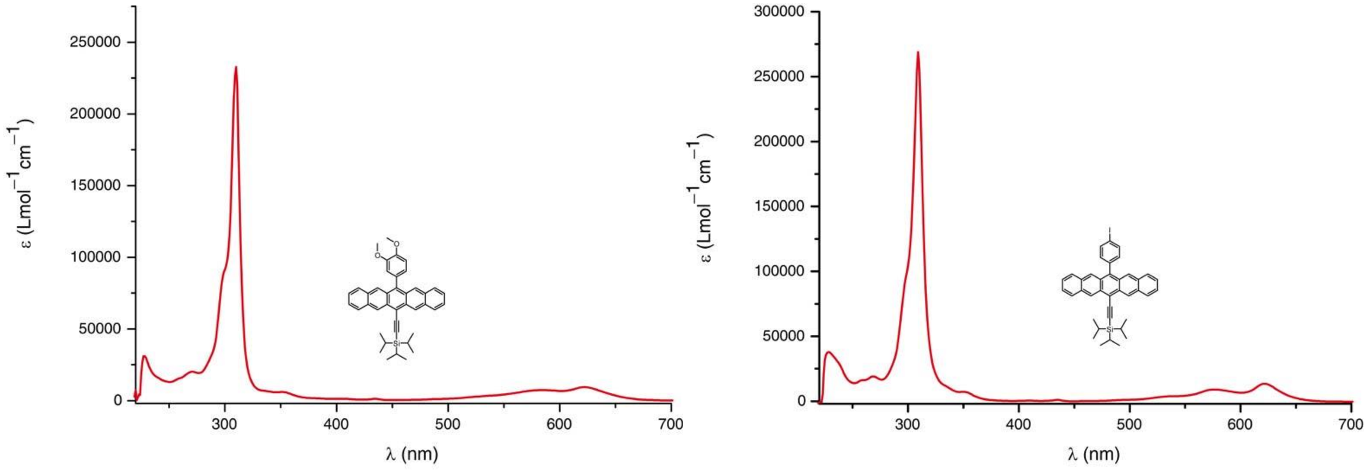

Figure S3: UV–vis spectra of pentacenes **3f** (left) and **3g** (right) in CH_2Cl_2.

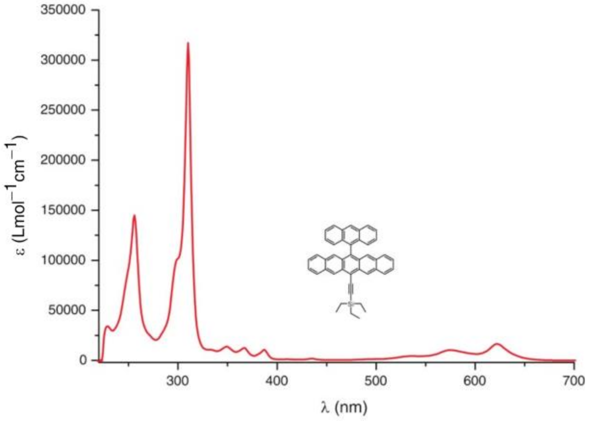

Figure S4: UV–vis spectra of pentacene **3h** in $CH_2Cl_{2.}$

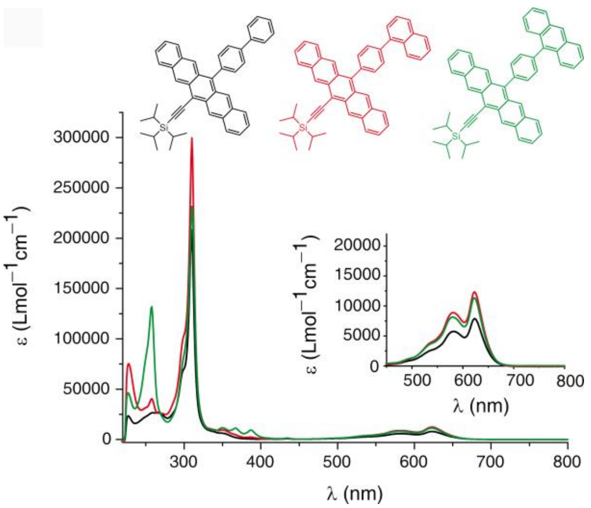

Figure S5: UV–vis spectra of pentacenes **3i–k** in $CH_2Cl_{2.}$

Emission Properties of Pentacenes 3a–k

3a

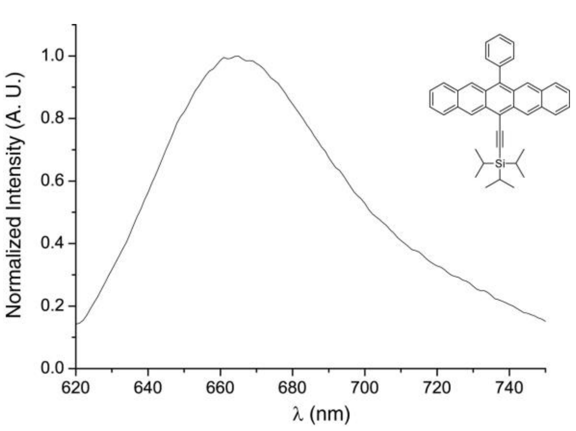

3b

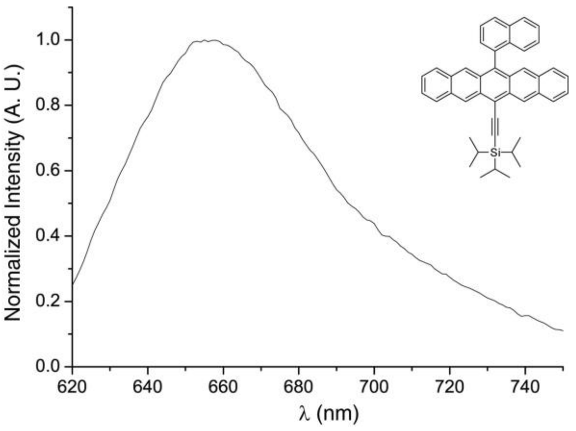

3C

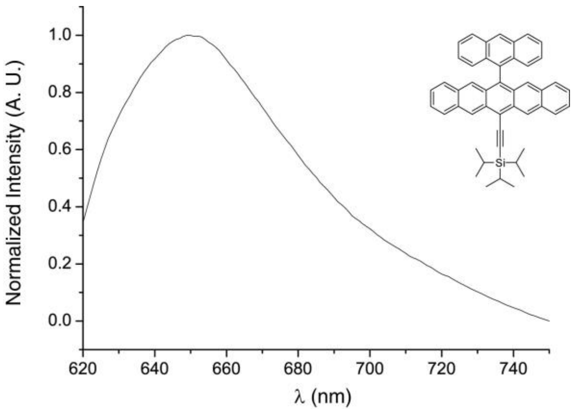

3d

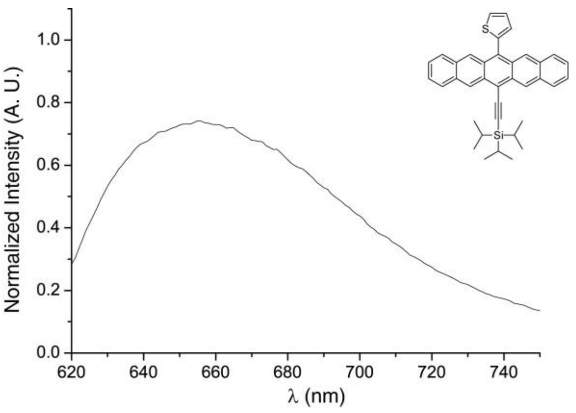

3e

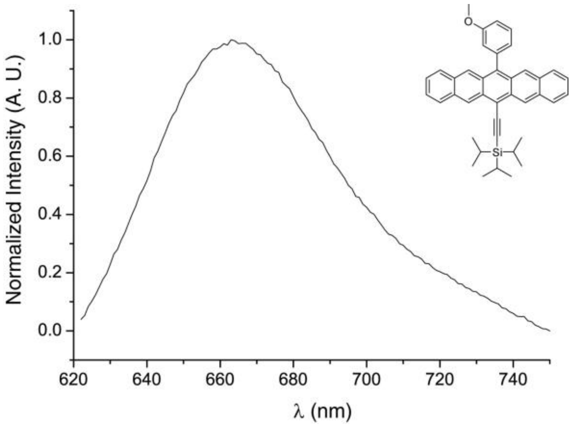

3f

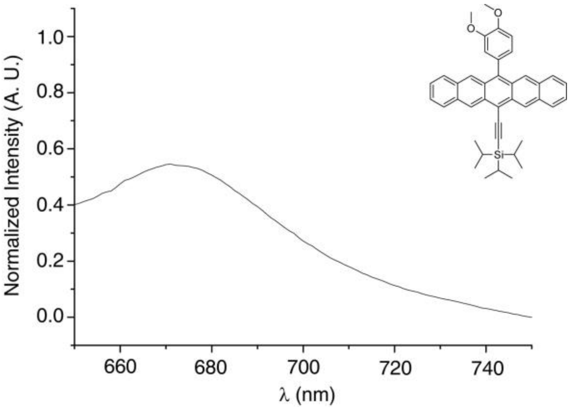

3g

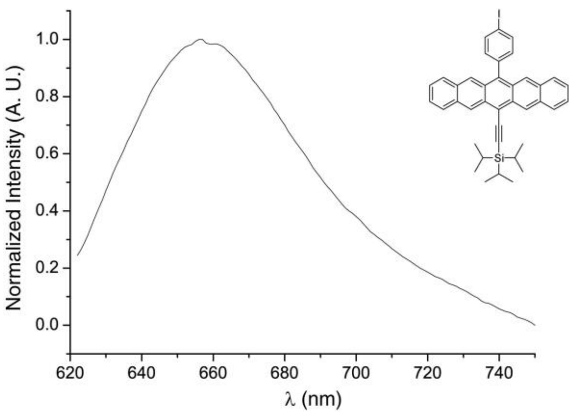

3h

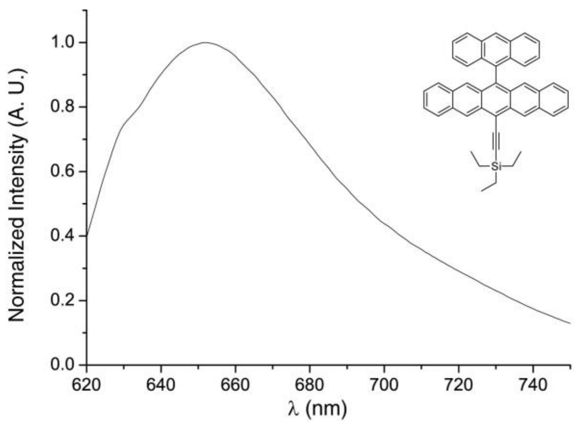

3i

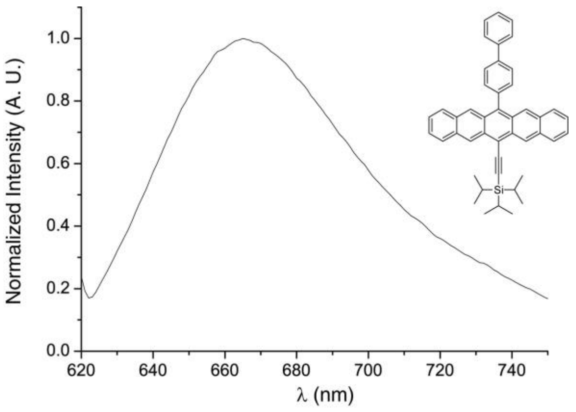

3j

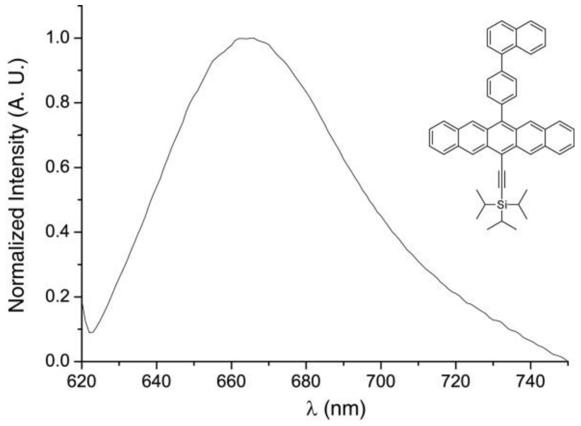

3k

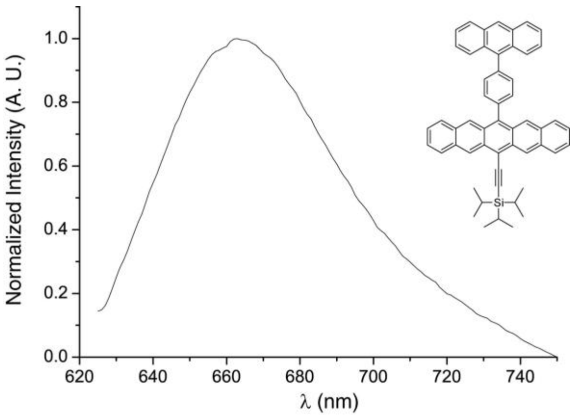

Figure S6: Normalized emission spectra of pentacenes **3a**–**k** (measured in CH_2Cl_2, ~10^{-8} M); excitation wavelength, see individual procedures.

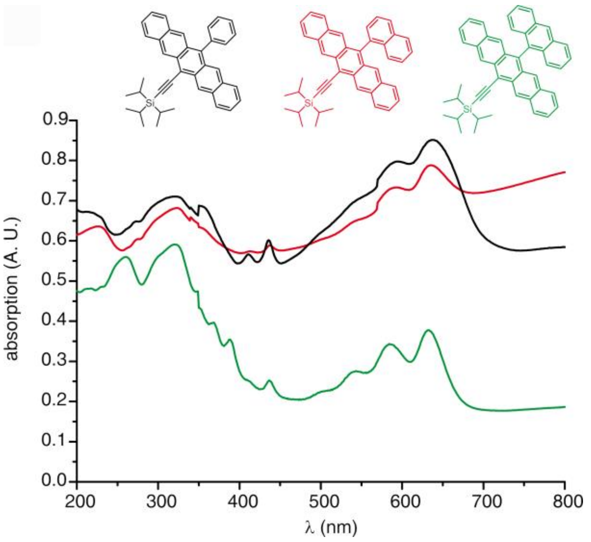

Figure S7: UV–vis spectra of thin films (drop cast on quartz from a CH_2Cl_2 solution) for pentacenes **3a**–**c**; absorption in arbitrary units (A.U.).

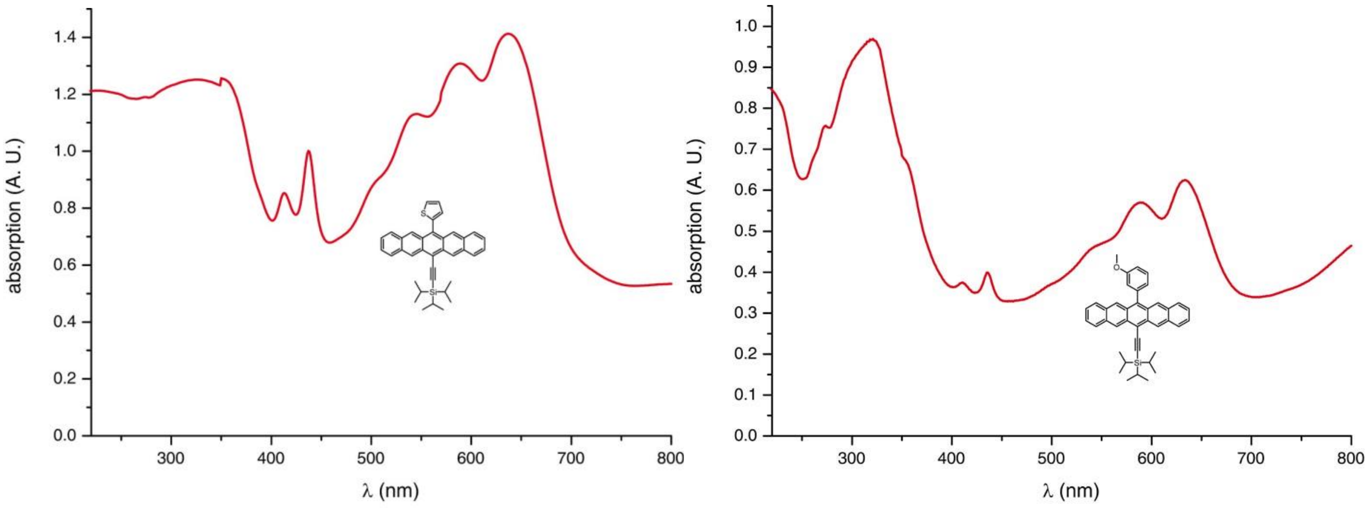

Figure S8: UV–vis spectra of thin films (drop cast on quartz from a CH_2Cl_2 solution) for pentacenes **3d** (left) and **3e** (right); absorption in arbitrary units (A.U.).

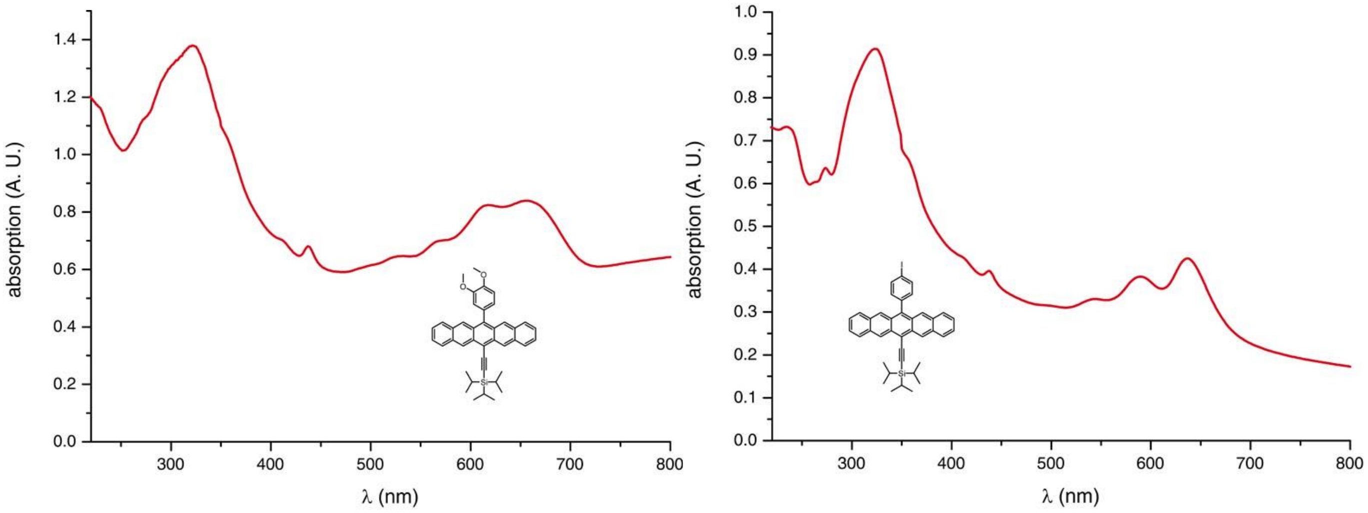

Figure S9: UV–vis spectra of thin films (drop cast on quartz from a CH_2Cl_2 solution) for pentacenes **3f** (left) and **3g** (right); absorption in arbitrary units (A.U.).

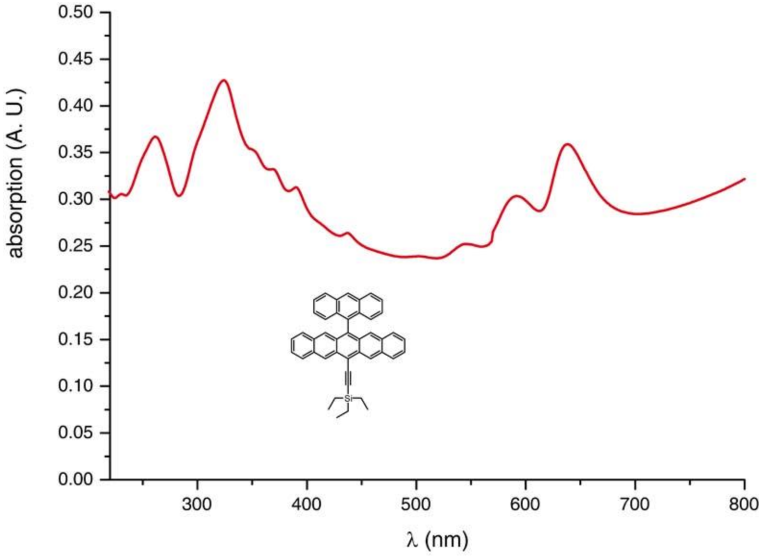

Figure S10: UV–vis spectra of thin film (drop cast on quartz from a CH_2Cl_2 solution) for pentacene **3h**; absorption in arbitrary units (A.U.).

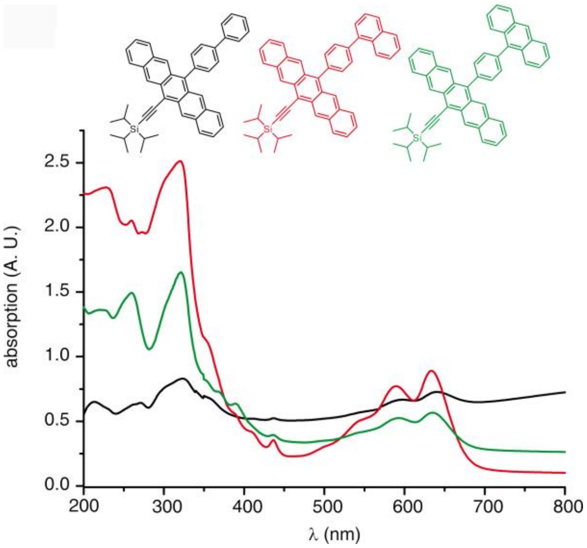

Figure S11: UV–vis spectra of thin films (drop cast on quartz from a CH_2Cl_2 solution) for pentacenes **3i**–**k**; absorption in arbitrary units (A.U.).

Packing of Pentacene 3h

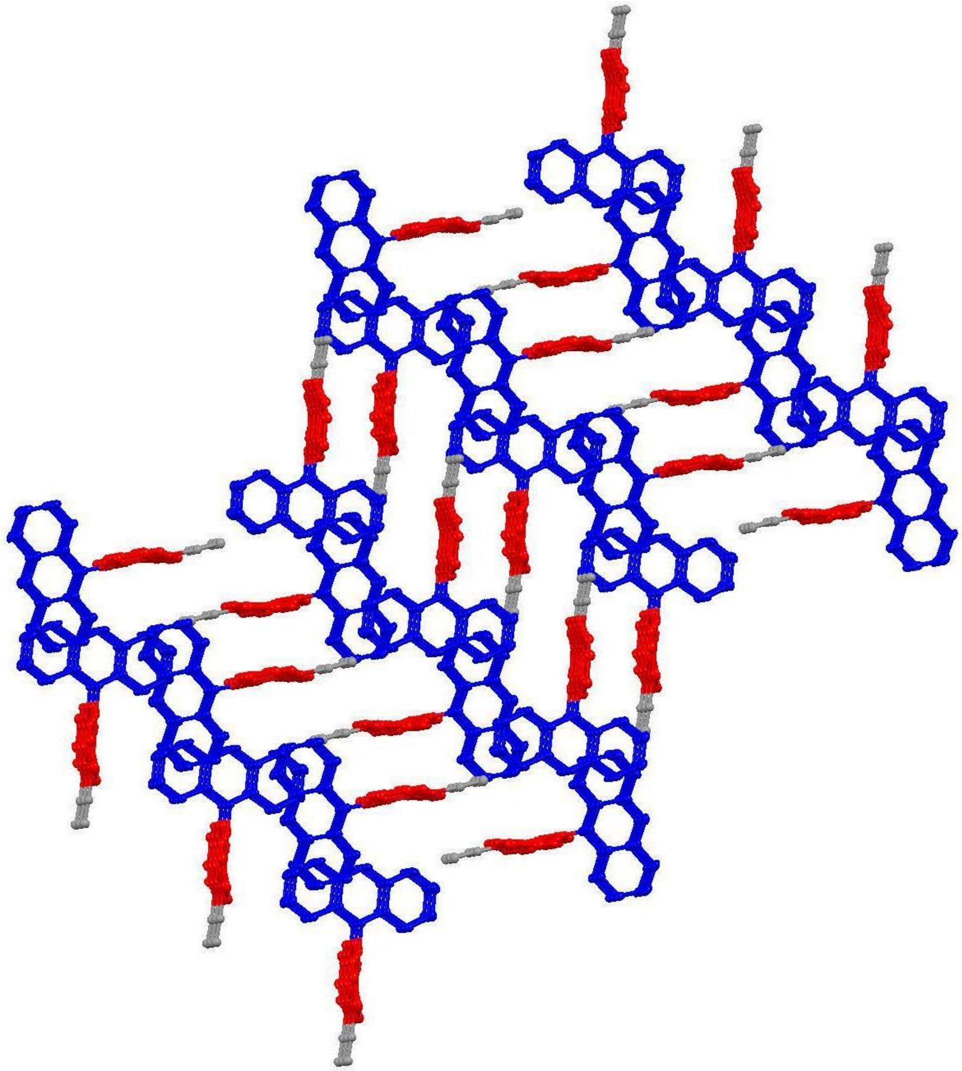

Figure S12: Packing of pentacene derivative **3h** (triethylsilyl groups omitted for clarity).

Electrochemical Properties of Pentacenes 3a–k

3a

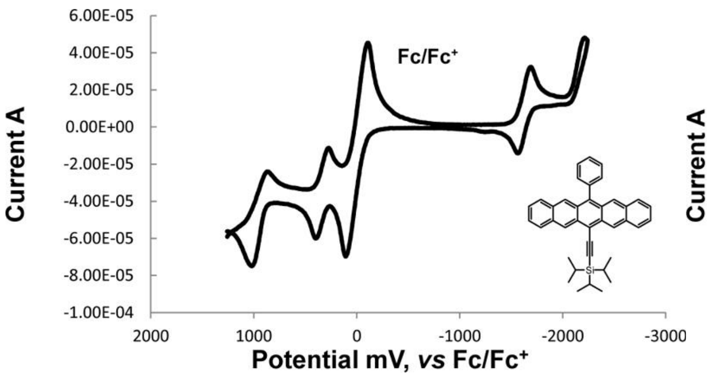

3b

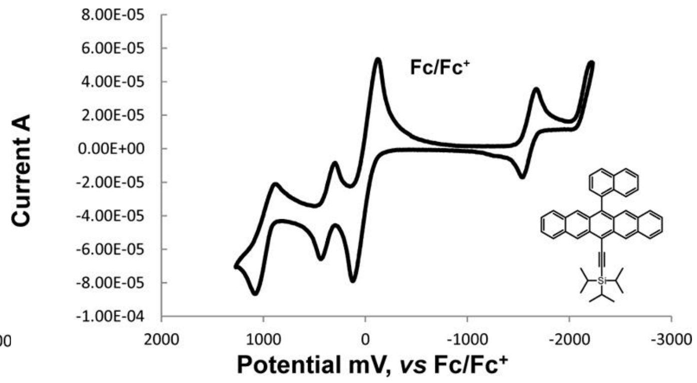

3c

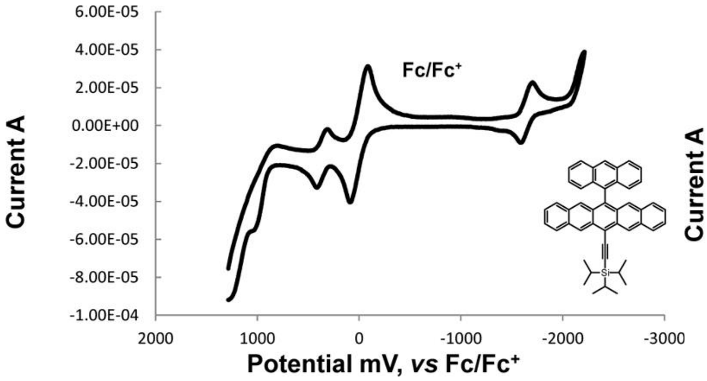

3d

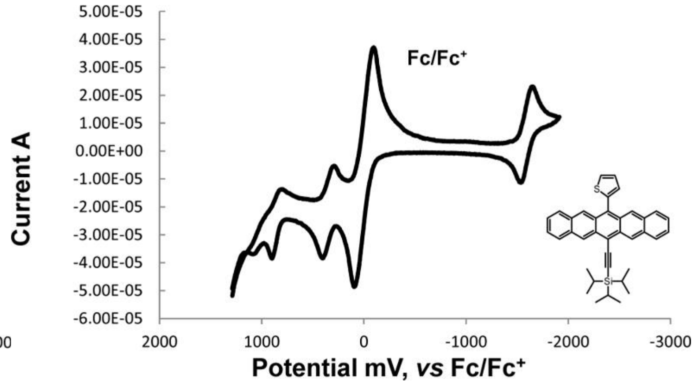

3e

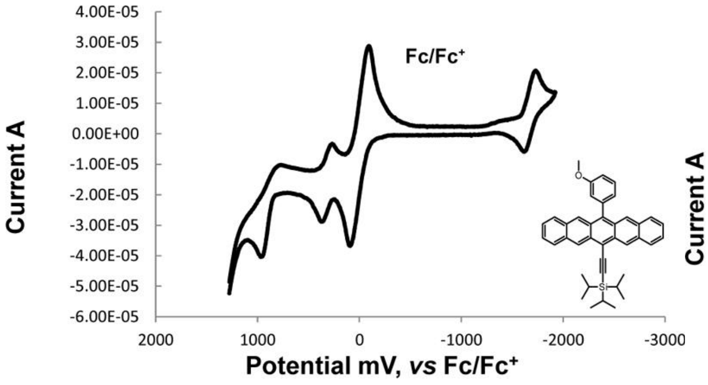

3f

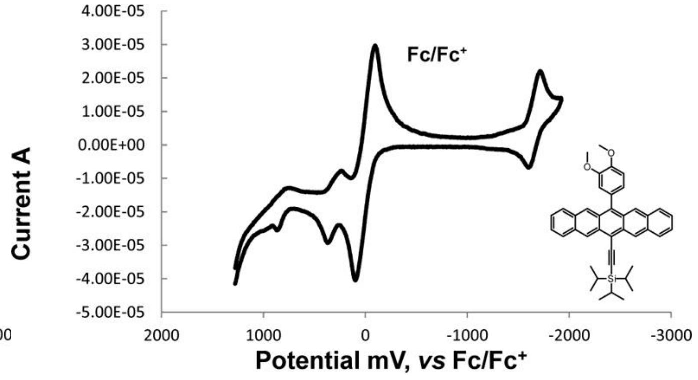

3g

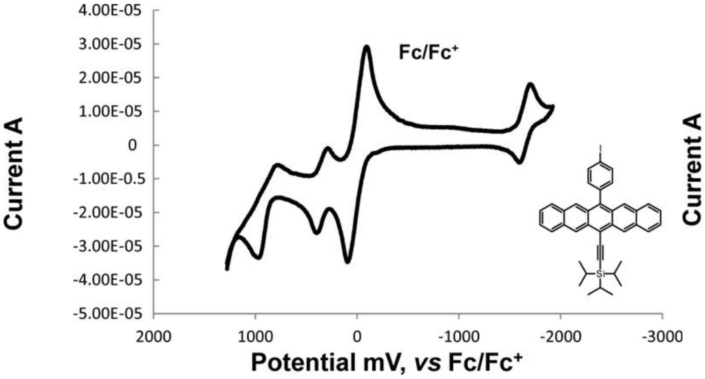

3h

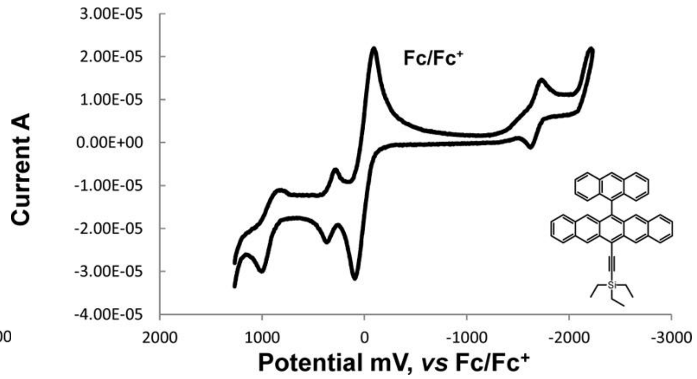

3i

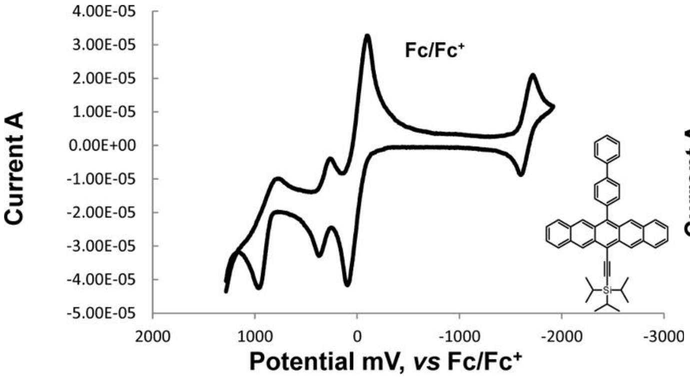

3j

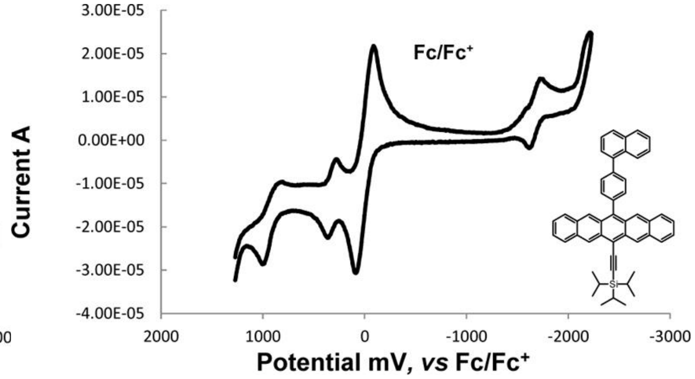

3k

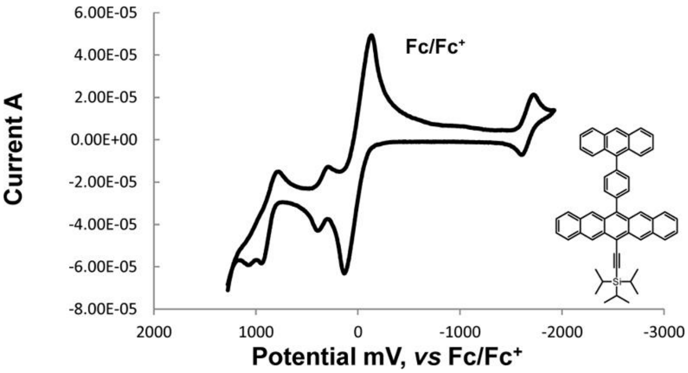

Figure S13: Cycic voltammograms for pentacenes **3a–k**.

Thermal Properties of Pentacenes 3a–k

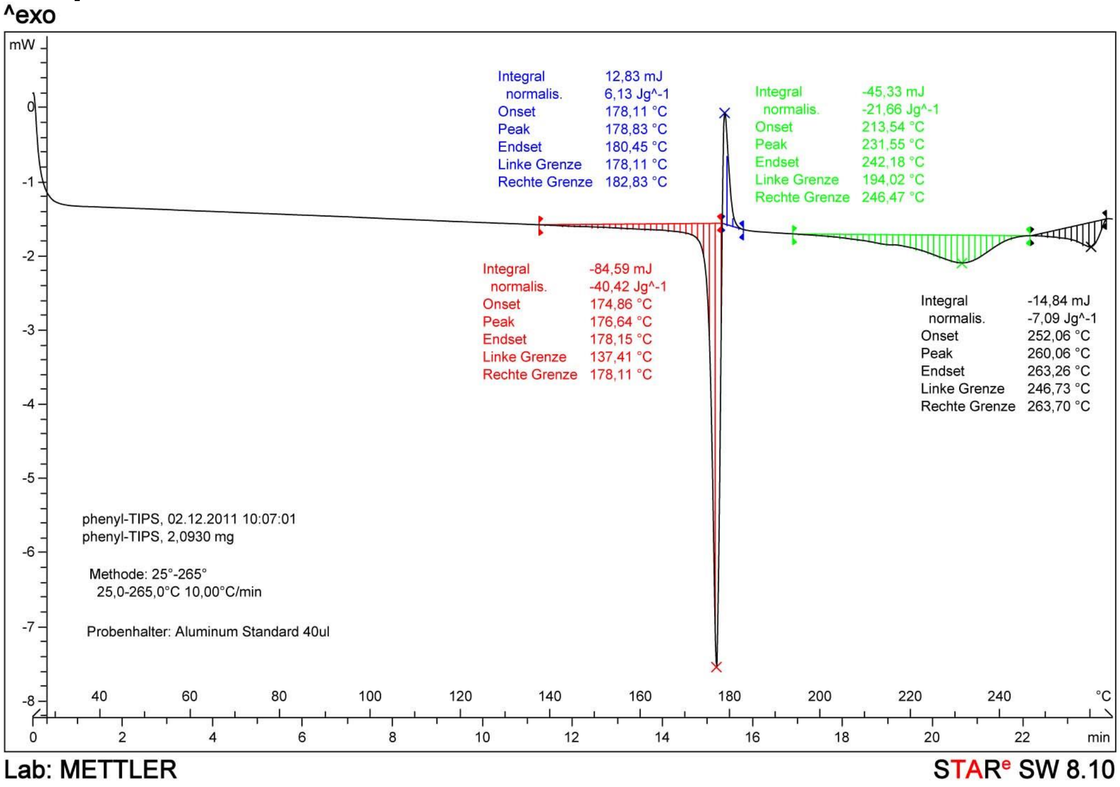

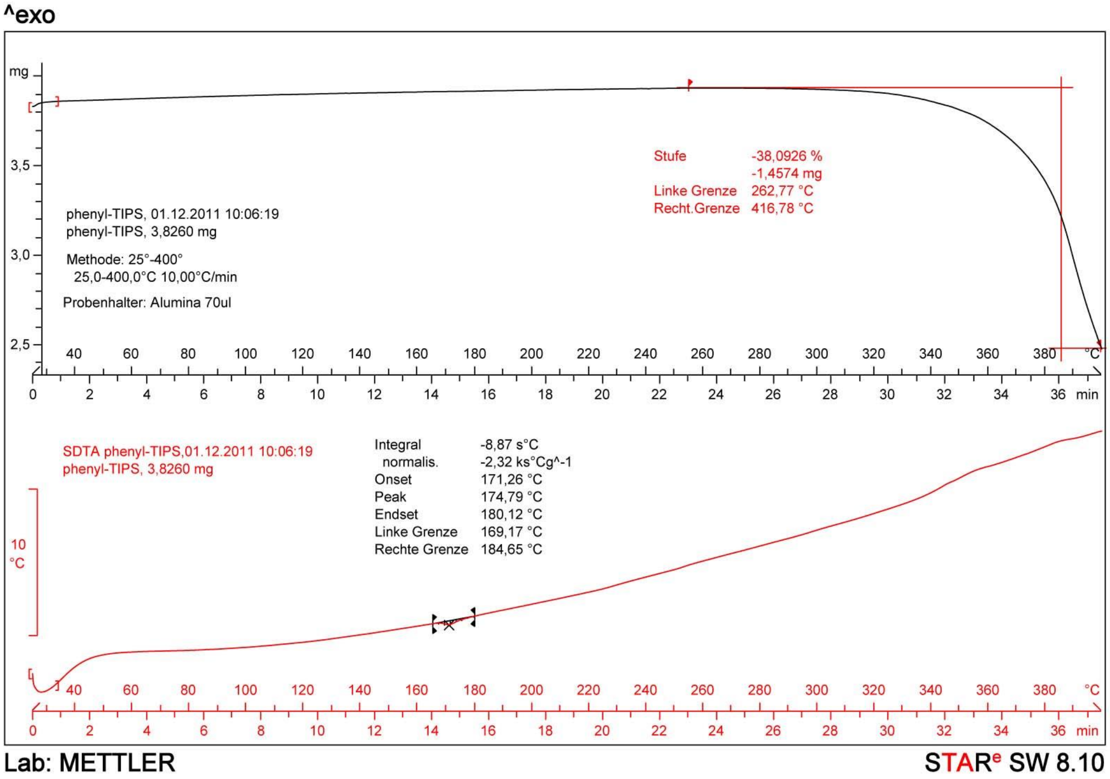

Figure S14: DSC (top) and TGA (bottom) analyses of pentacene **3a**.

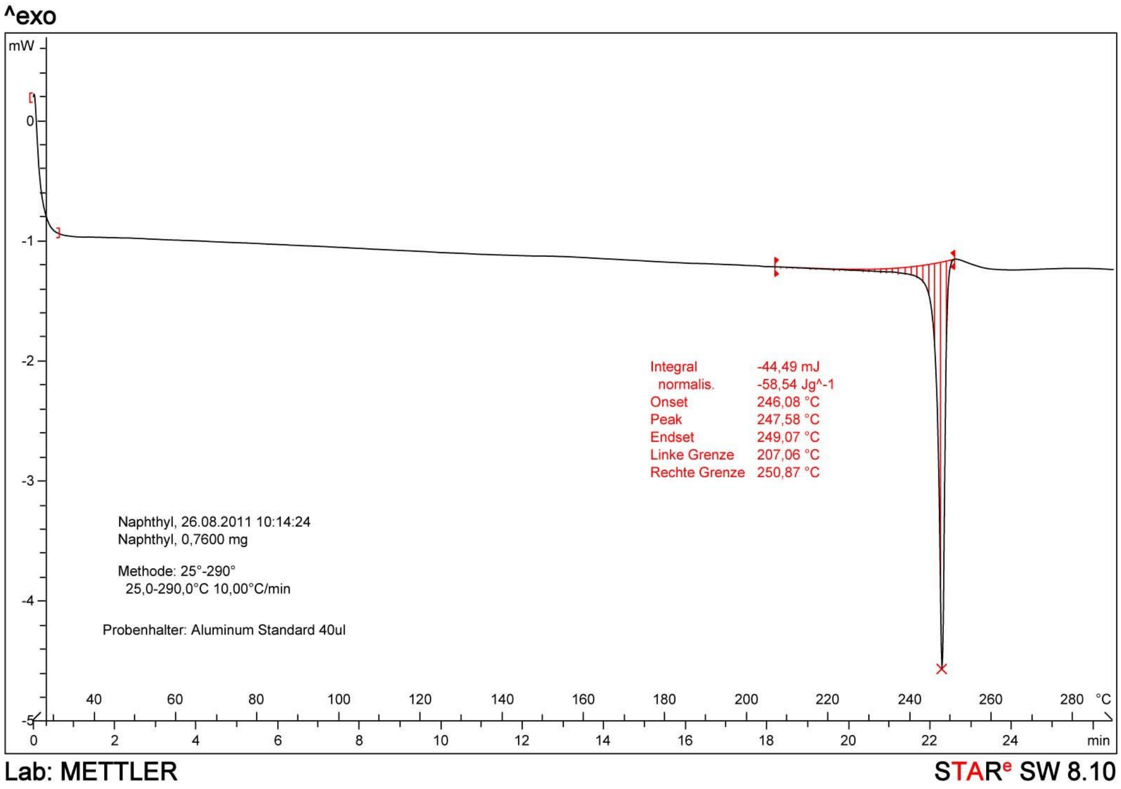

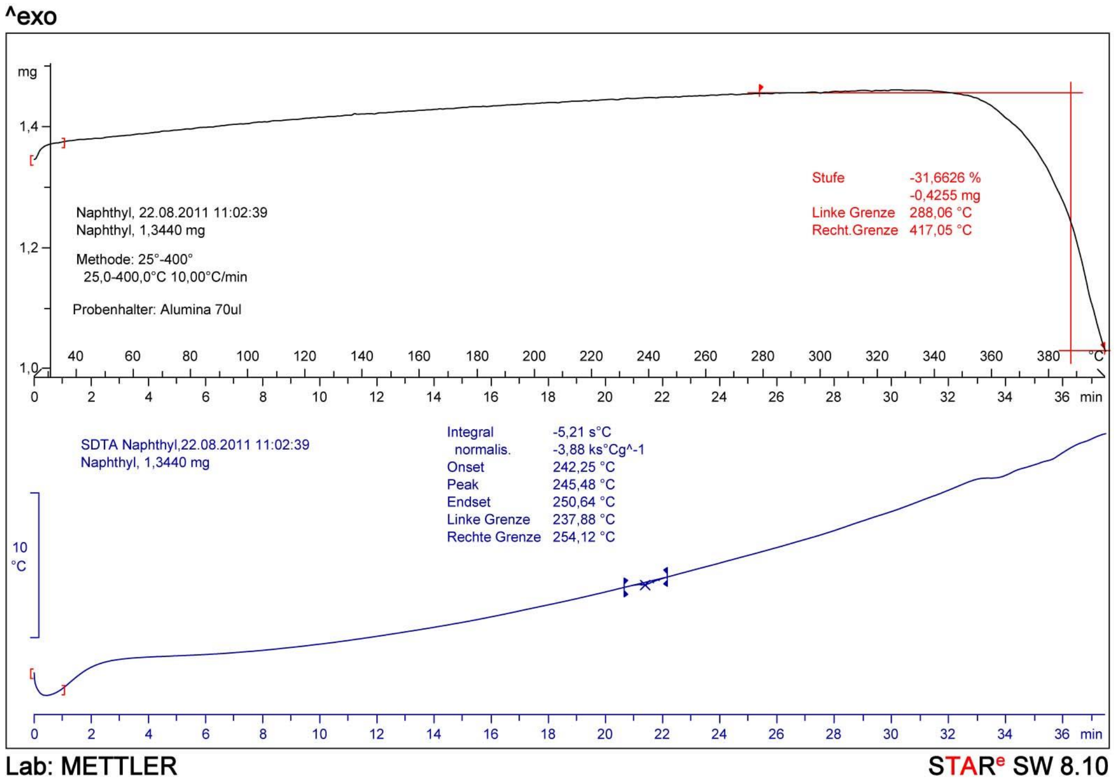

Figure S15: DSC (top) and TGA (bottom) analyses of pentacene **3b**.

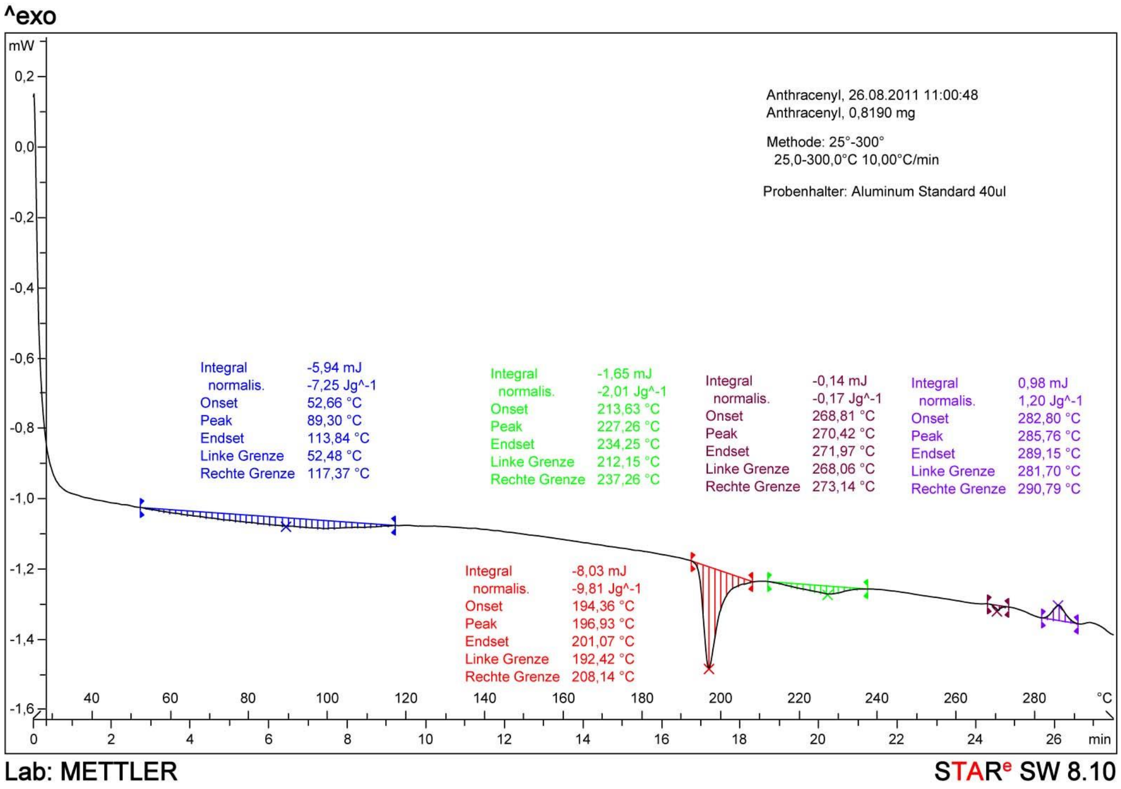

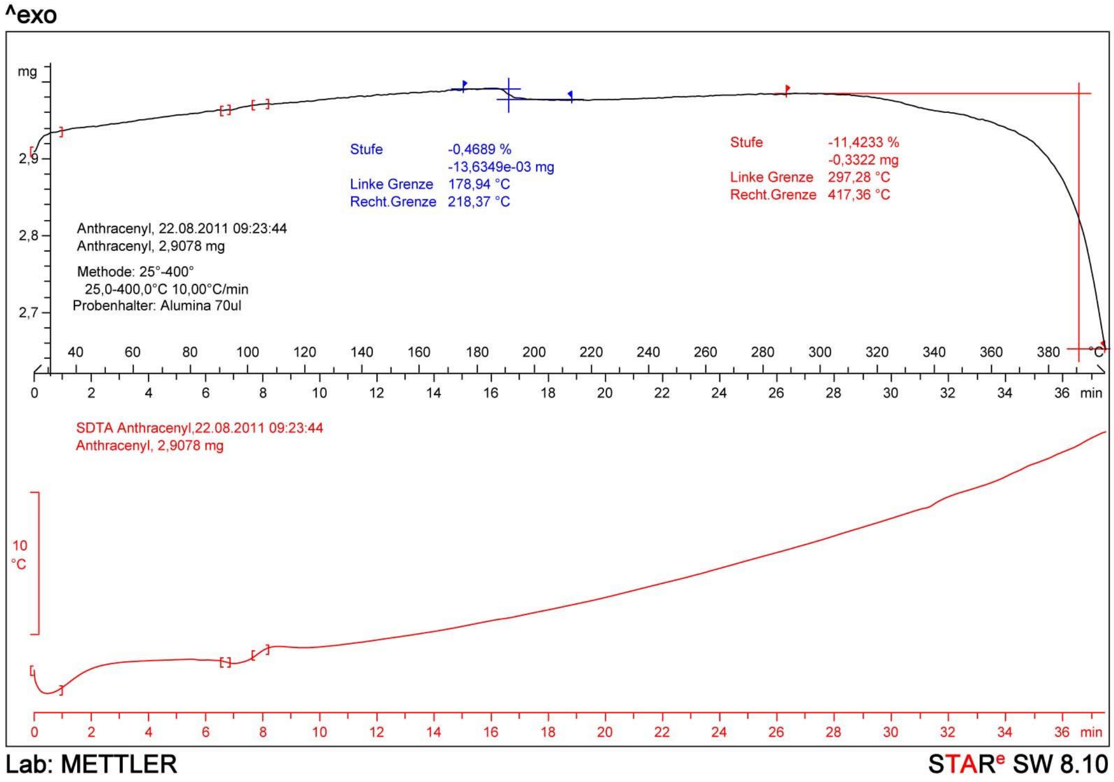

Figure S16: DSC (top) and TGA (bottom) analyses of pentacene **3c**.

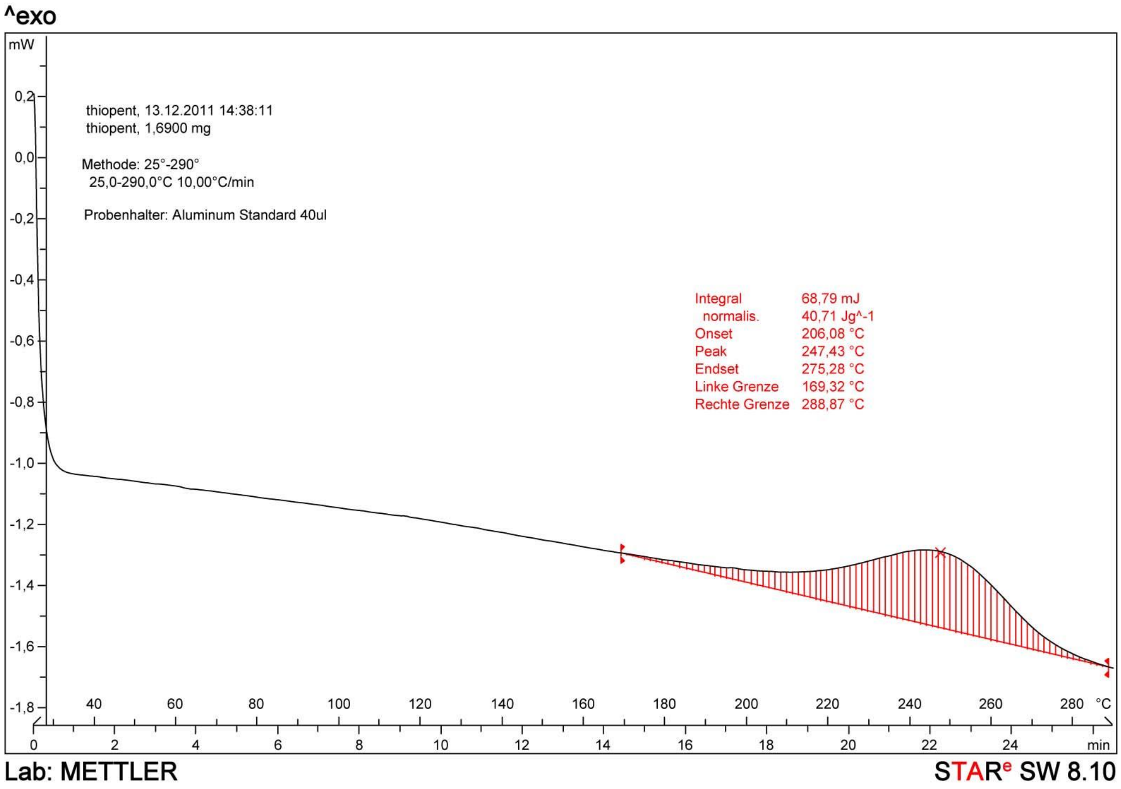

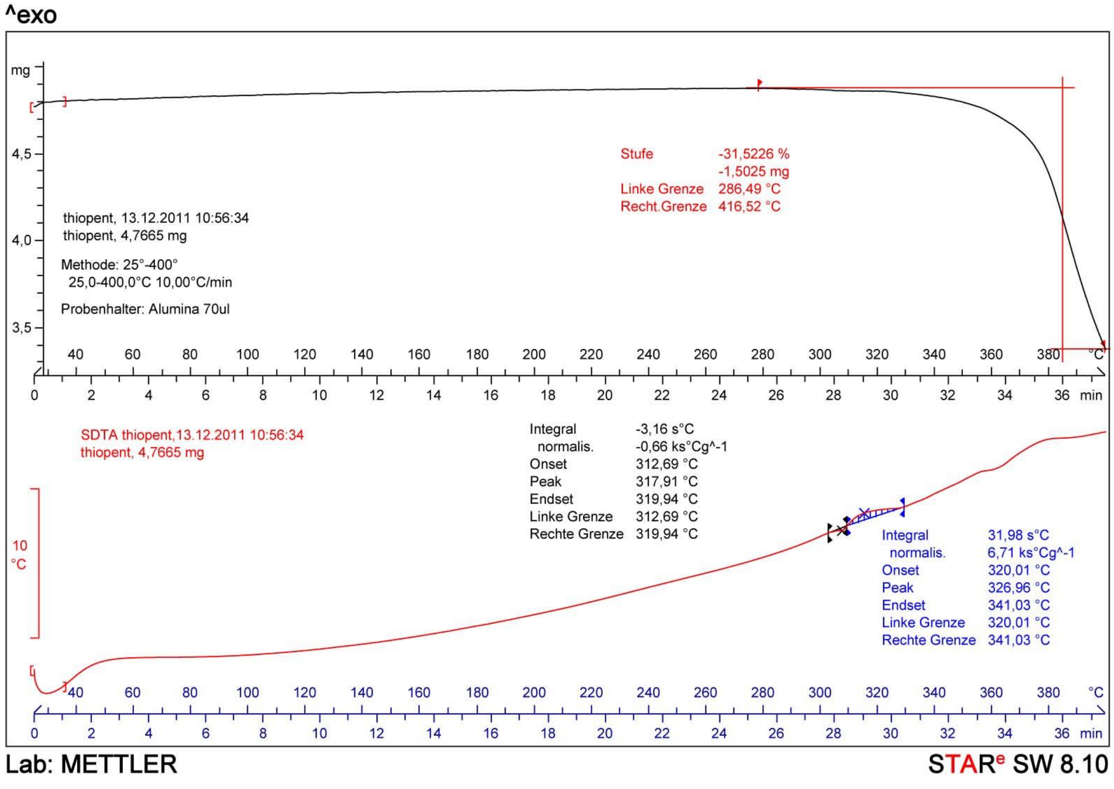

Figure S17: DSC (top) and TGA (bottom) analyses of pentacene **3d**.

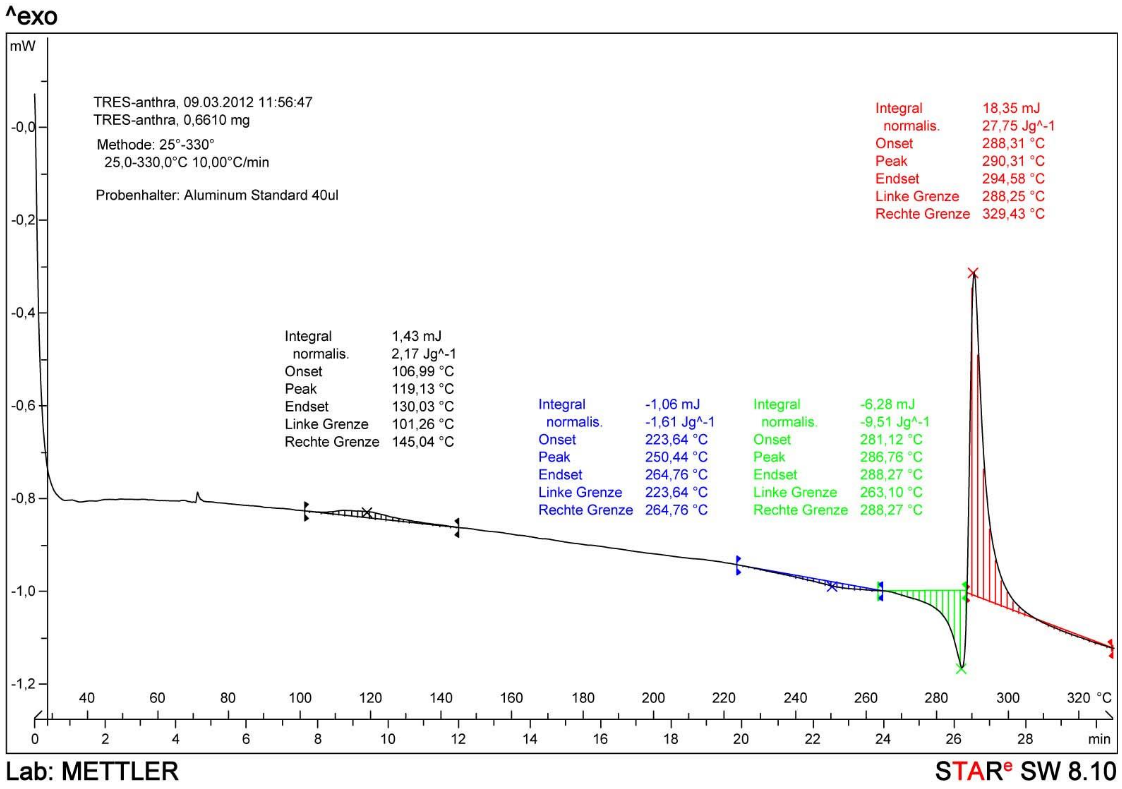

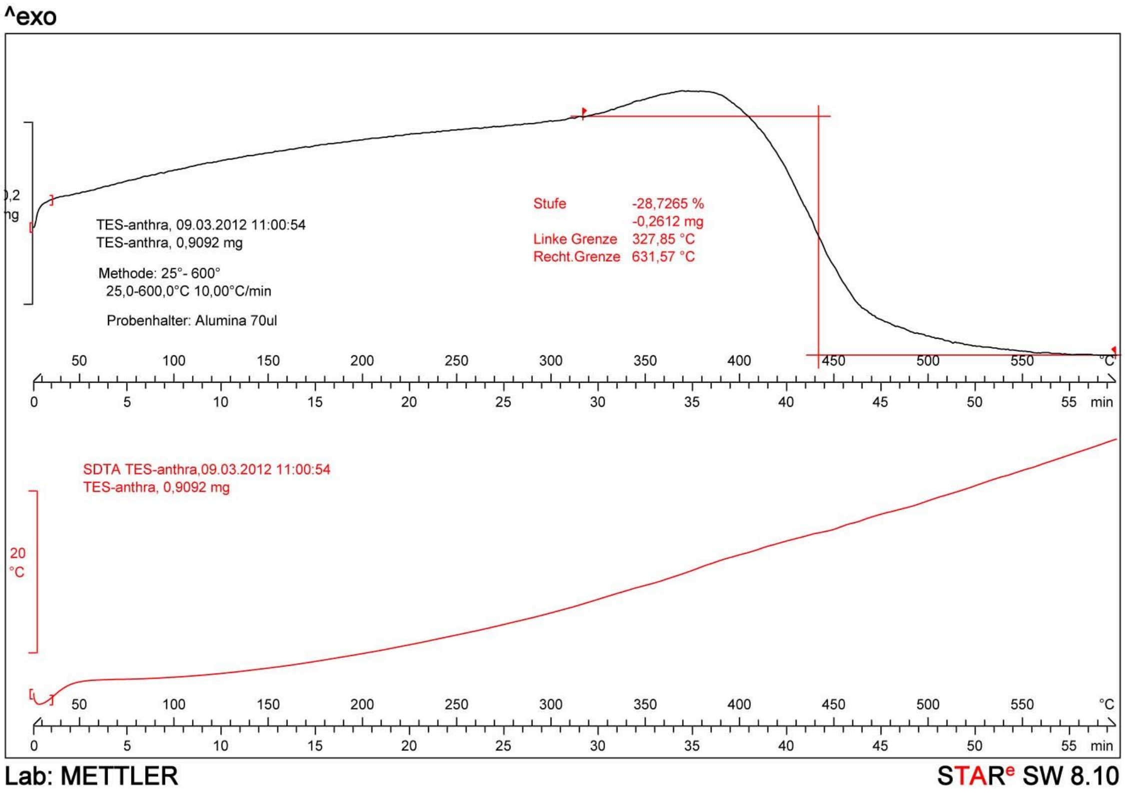

Figure S18: DSC (top) and TGA (bottom) analyses of pentacene **3h**.

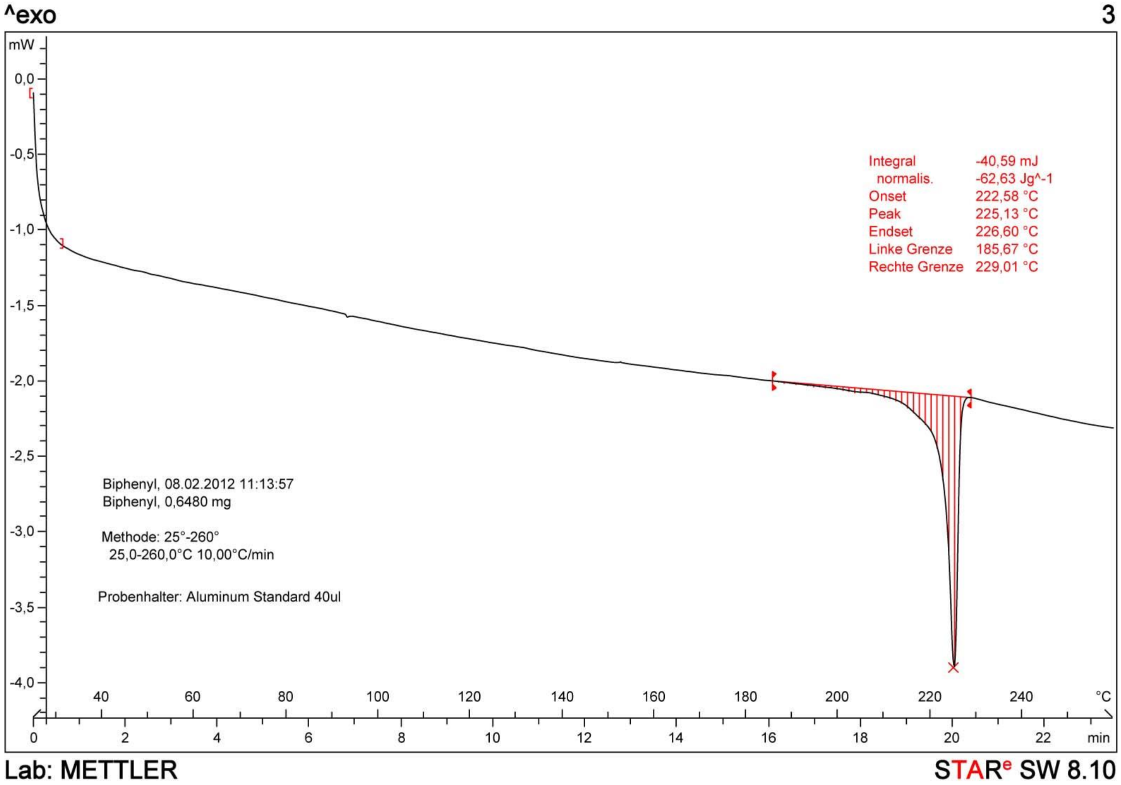

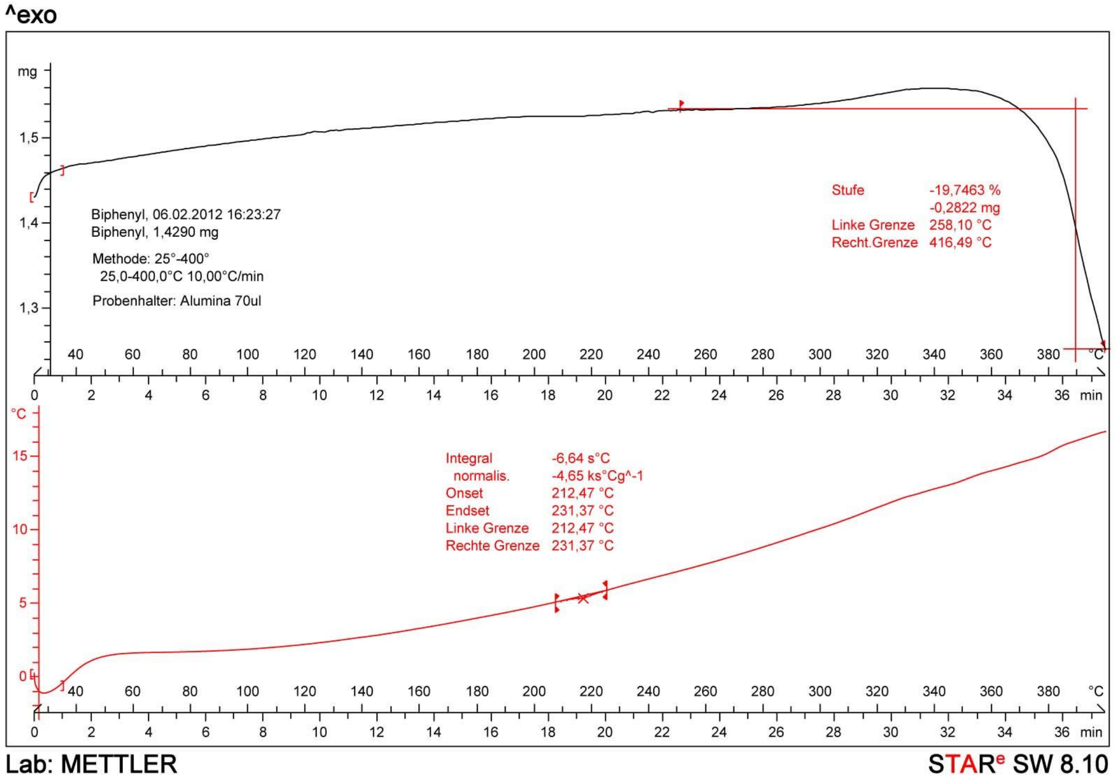

Figure S19: DSC (top) and TGA (bottom) analyses of pentacene **3i**.

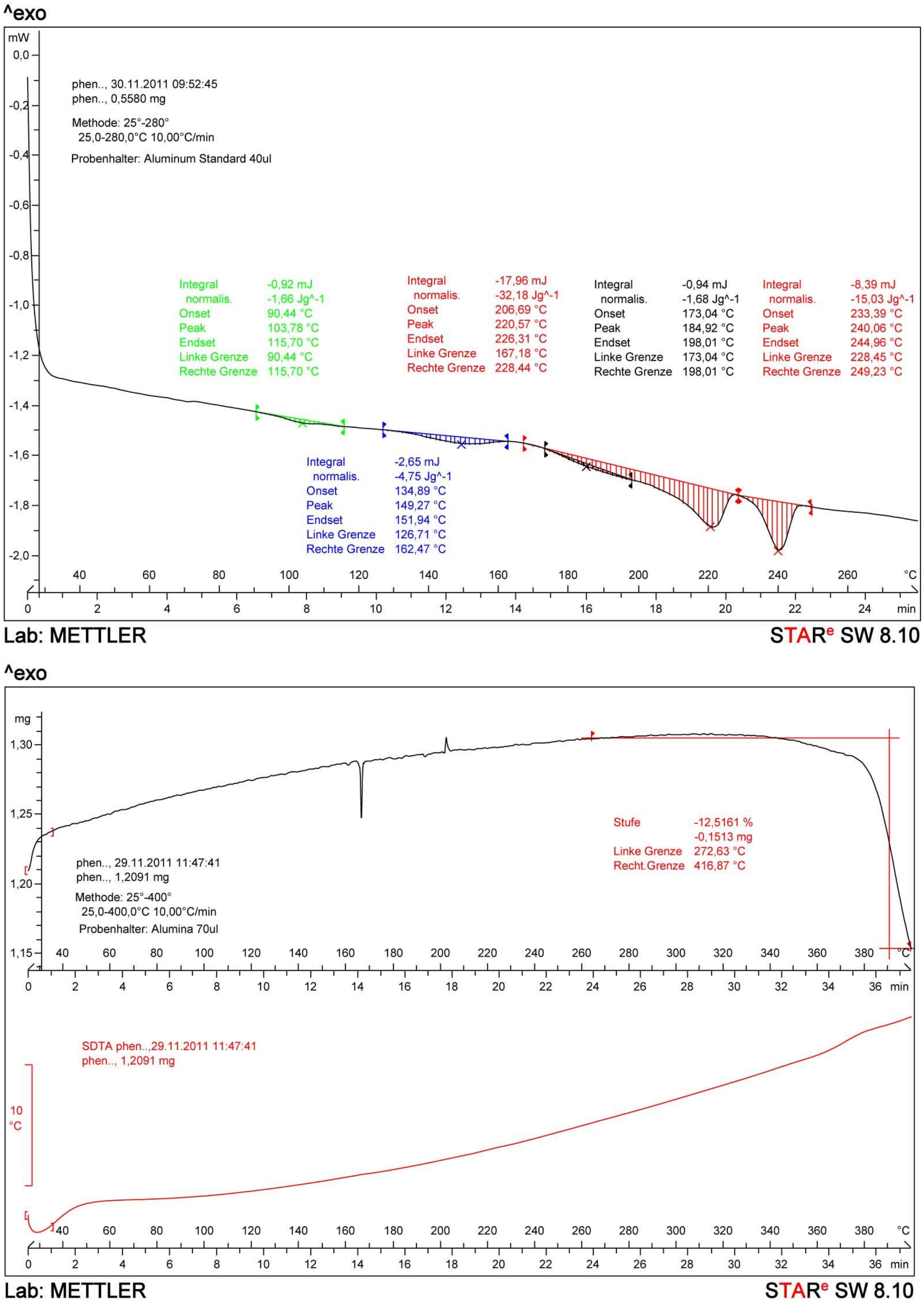

Figure S20: DSC (top) and TGA (bottom) analyses of pentacene **3j**.

REFERENCES

[1] Lehnherr, D.; Murray, A. H.; McDonald, R.; Tykwinski, R. R; *Angew. Chem. Int.* Ed. **2010**, *49*, 6190–6194.

[2] Etschel, S. H.; Waterloo, A. R.; Markgraf, J.; Amin, A. Y.; Hampel, F.; Jäger, C. M.; Clark, T.; Halik, M.; Tykwinski, R. R. *Chem Comm.* **2013**, *49*, 6725–6727.

^{1}H and ^{13}C NMR spectra of pentacenes 3a–k

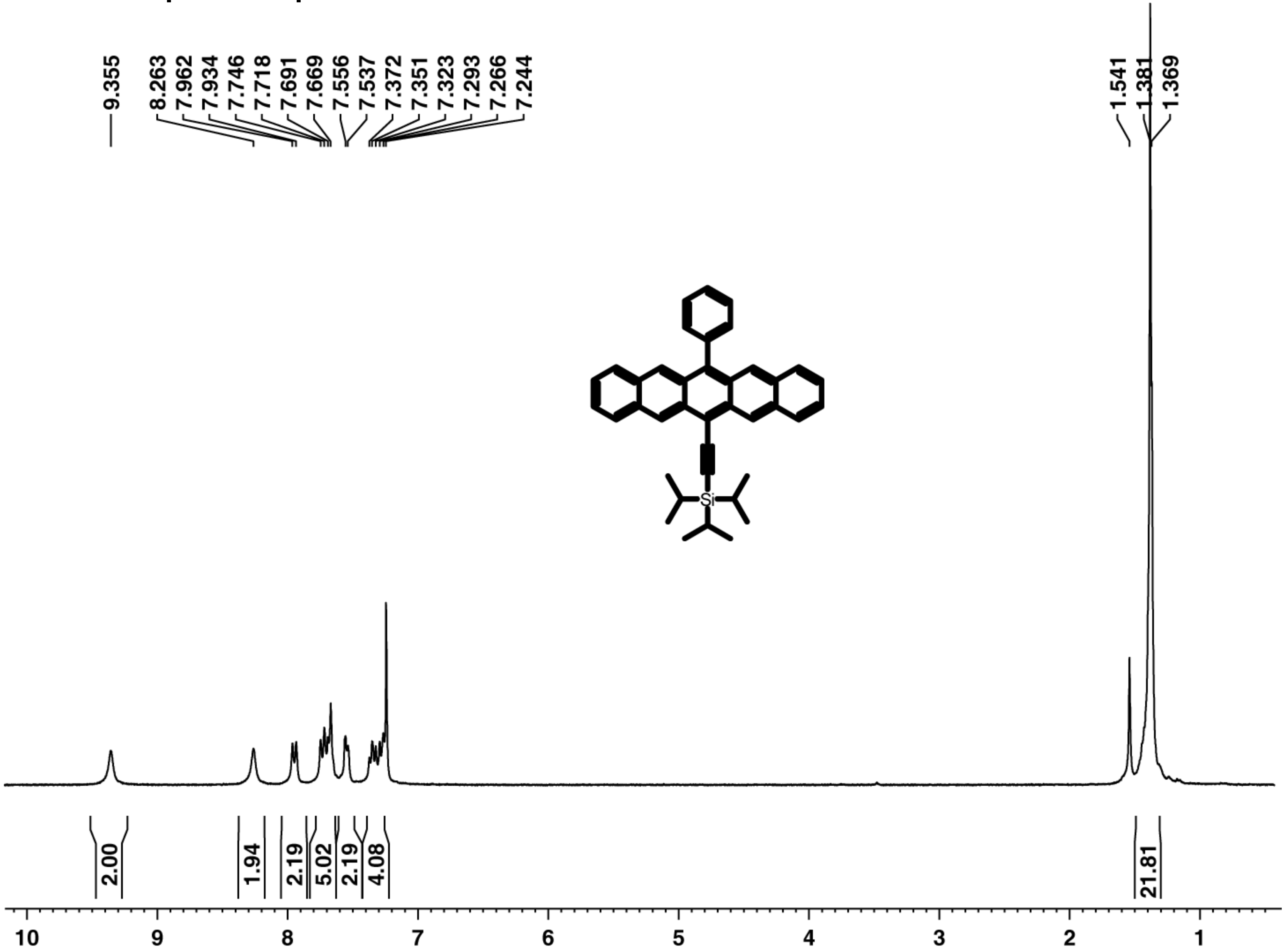

Figure S21: ^{1}H NMR (300 MHz, $CDCl_3$, rt) of pentacene **3a**.

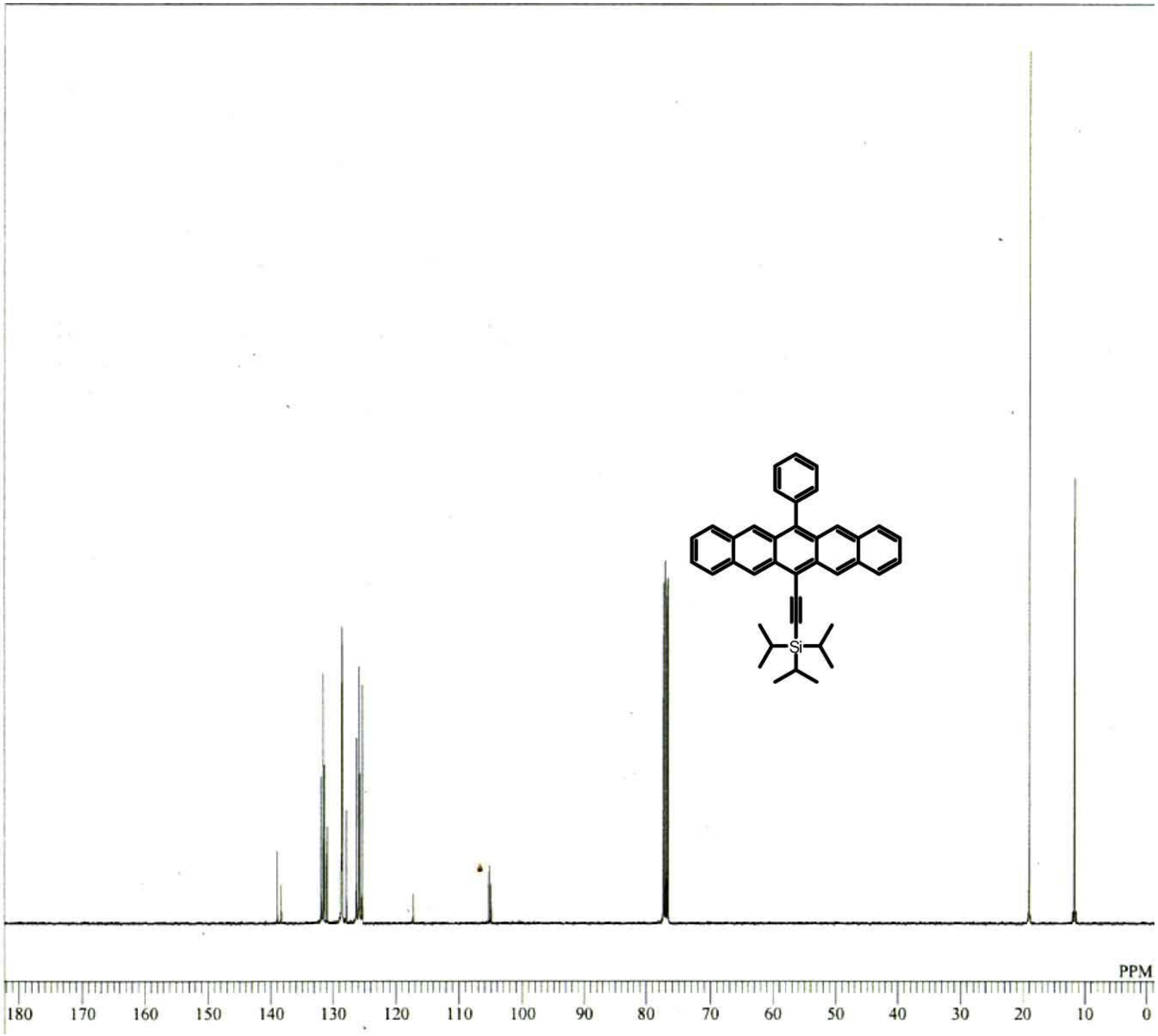

Figure S22: ^{13}C NMR (100 MHz, $CDCl_3$, rt) of pentacene **3a**.

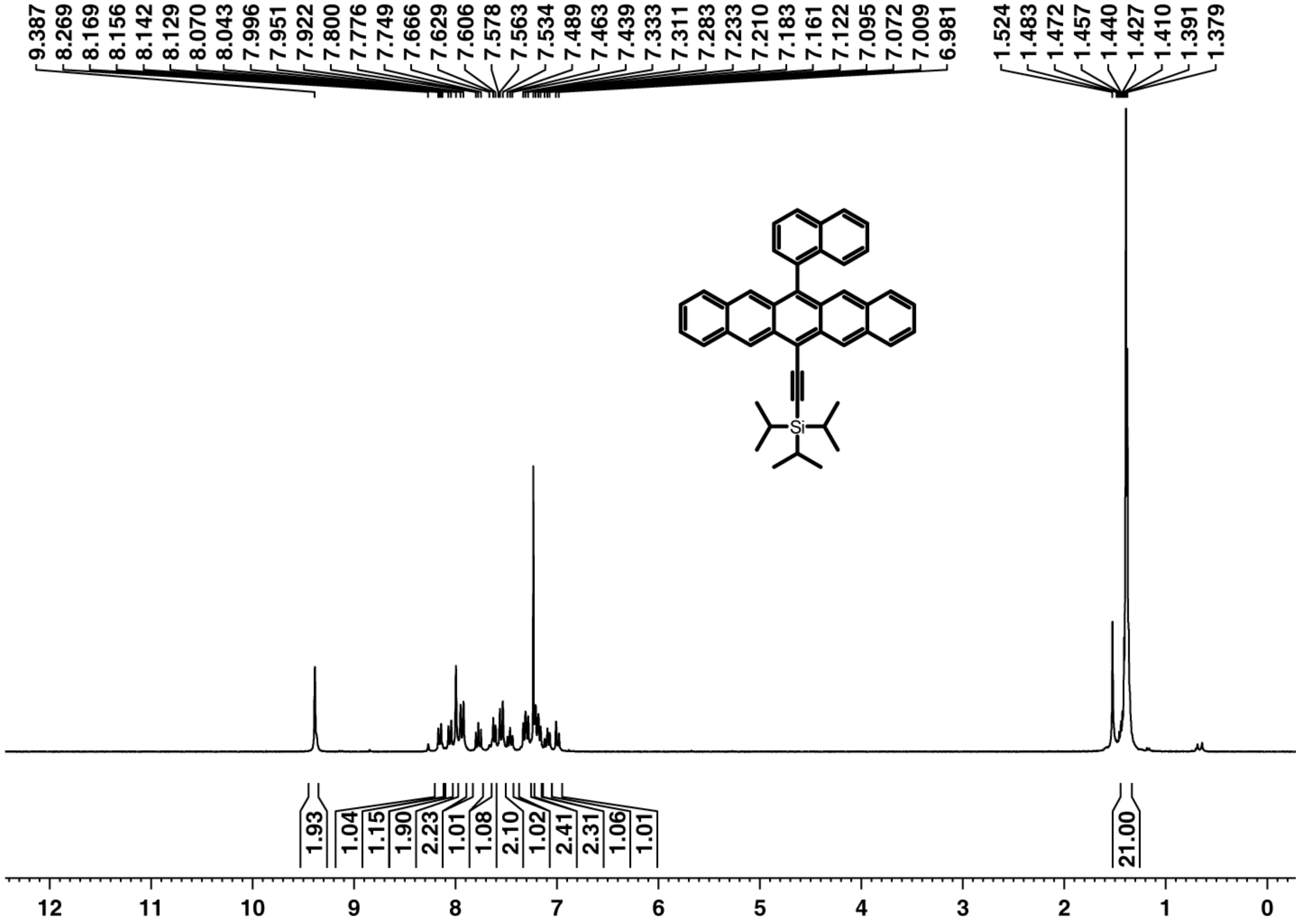

Figure S23: ^{1}H NMR (300 MHz, $CDCl_3$, rt) of pentacene **3b**.

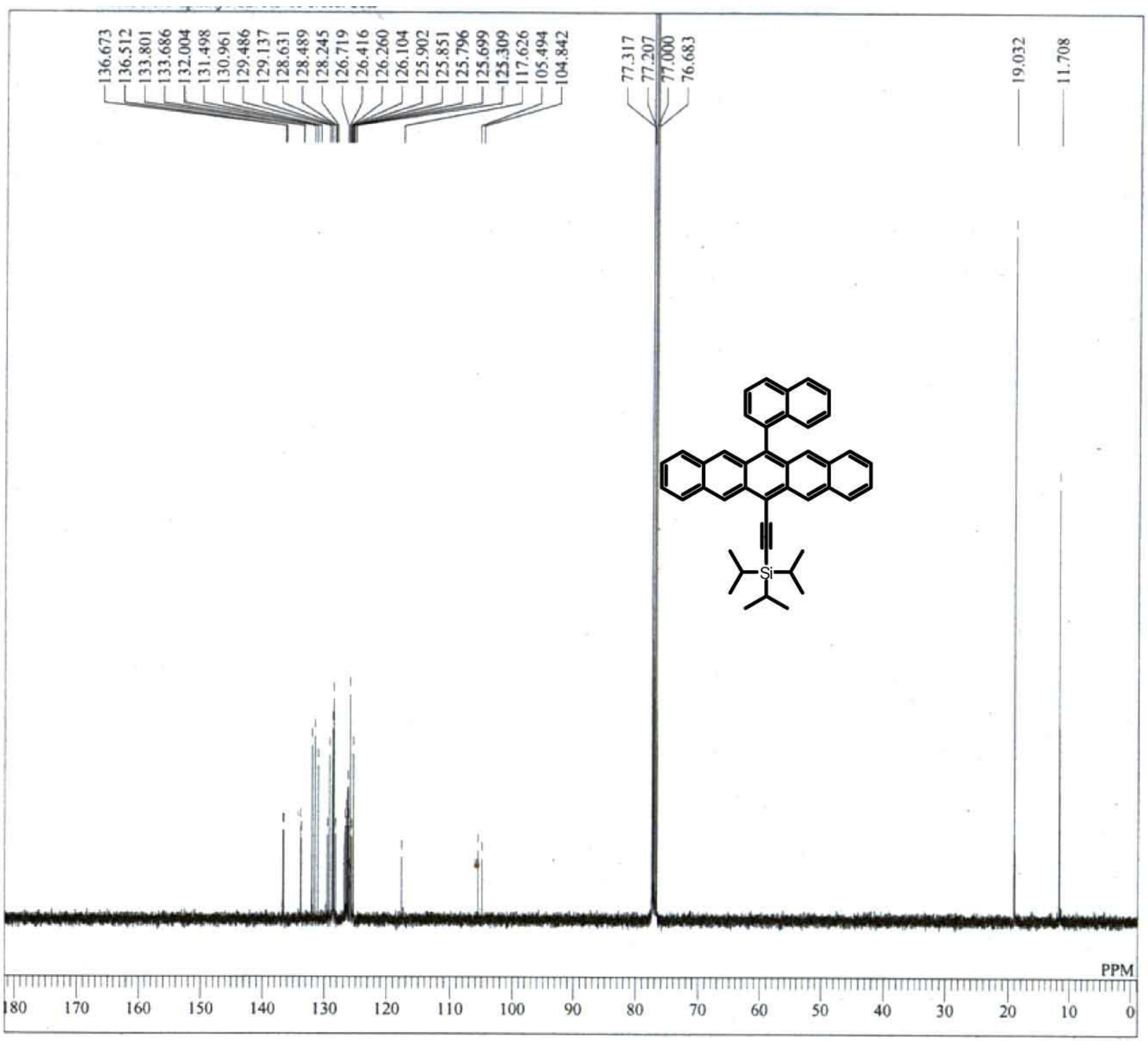

Figure S24: ^{13}C NMR (100 MHz, $CDCl_3$, rt) of pentacene **3b**.

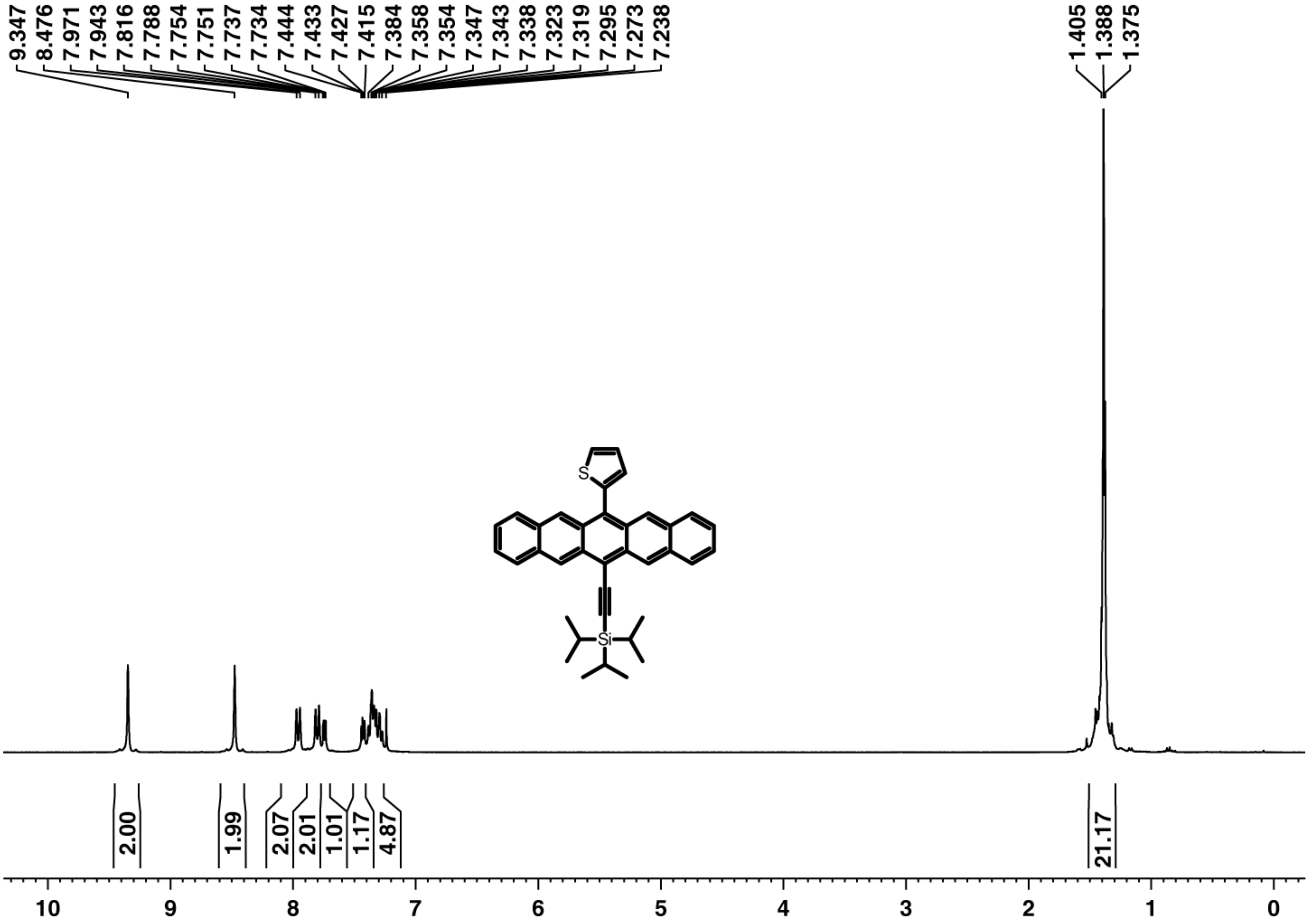

Figure S25: ^{1}H NMR (300 MHz, $CDCl_3$, rt) of pentacene **3d**.

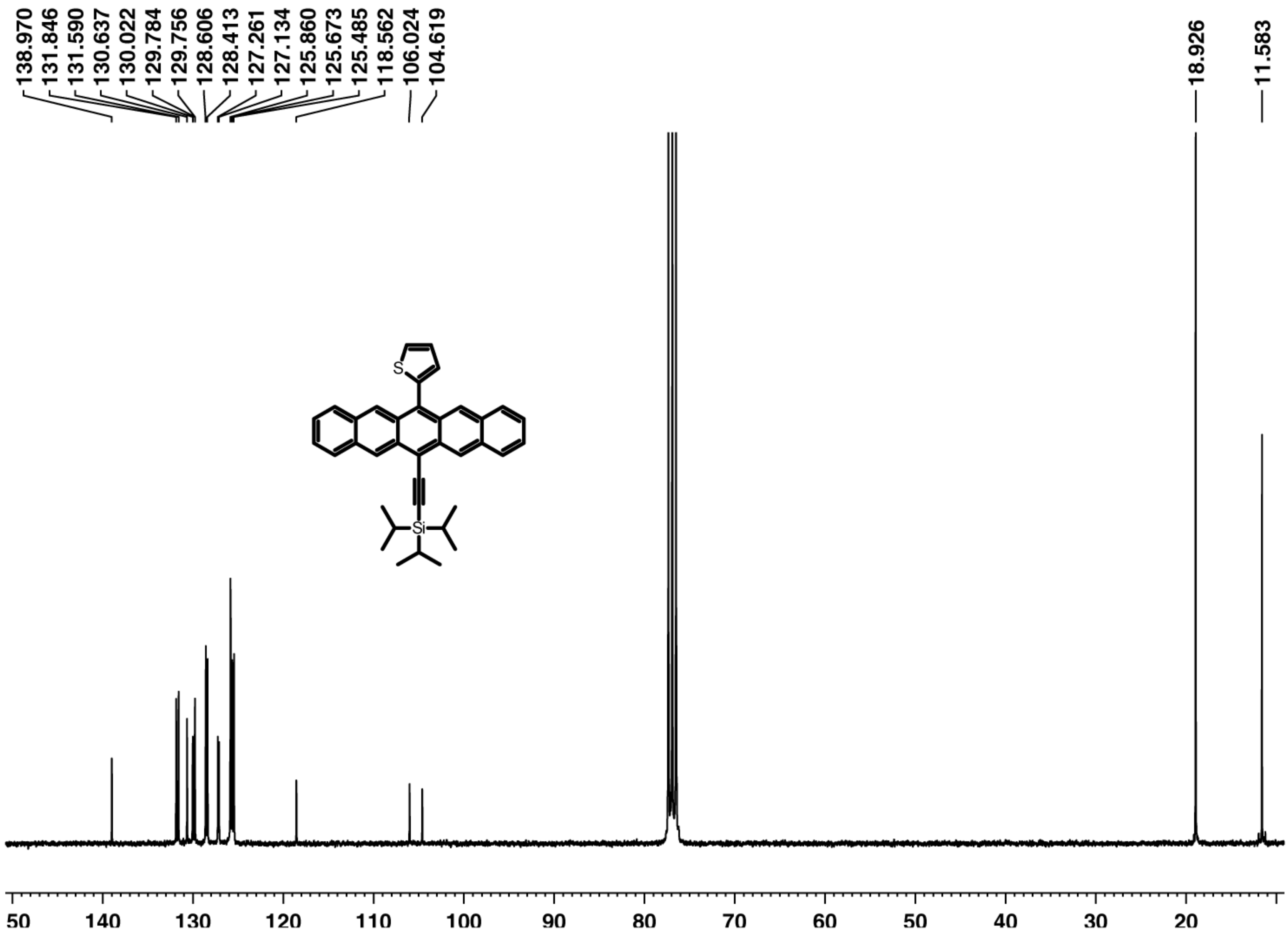

Figure S26: ^{13}C NMR (75 MHz, $CDCl_3$, rt) of pentacene **3d**.

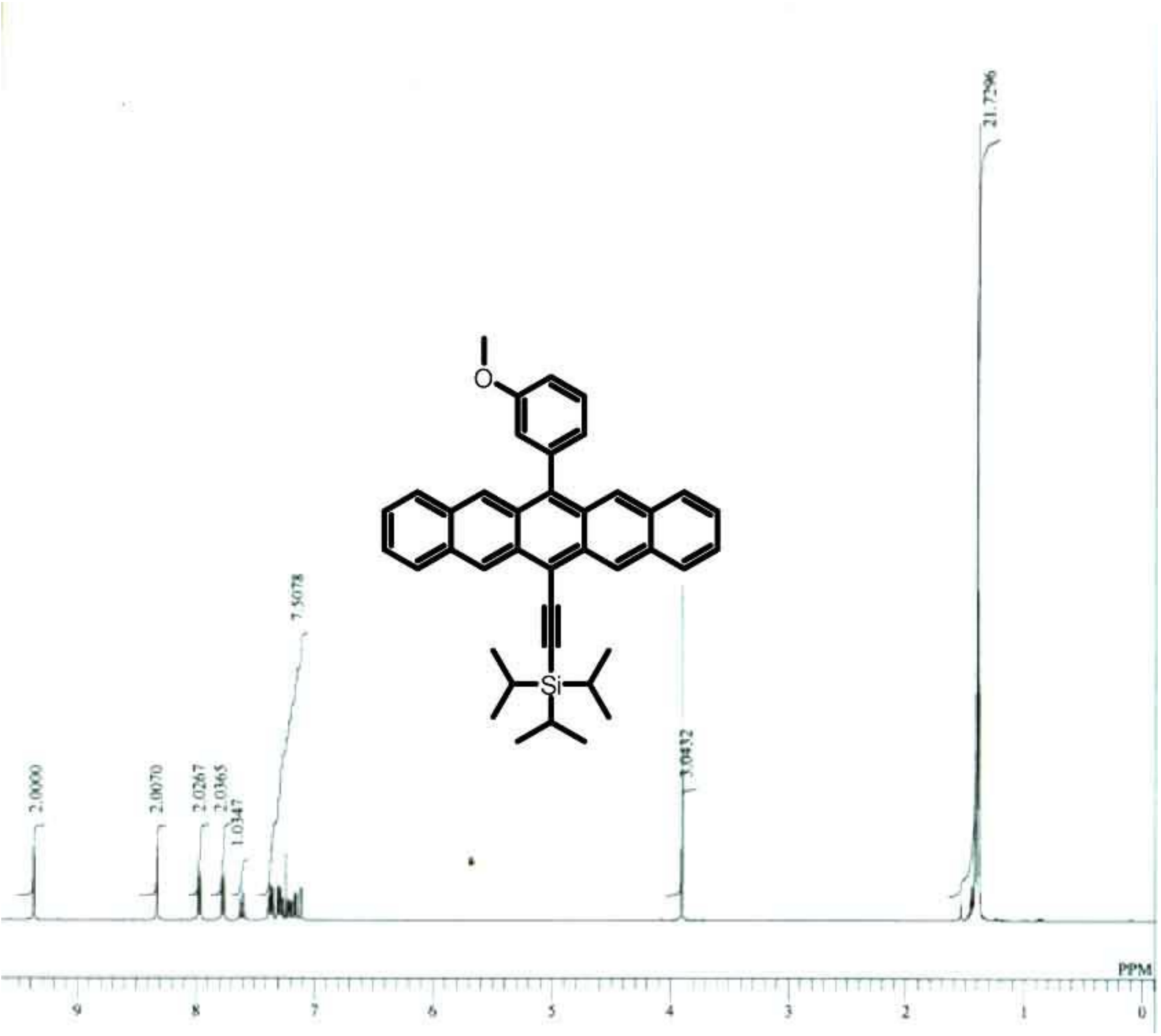

Figure S27: ^{1}H NMR (300 MHz, $CDCl_3$, rt) of pentacene **3e**.

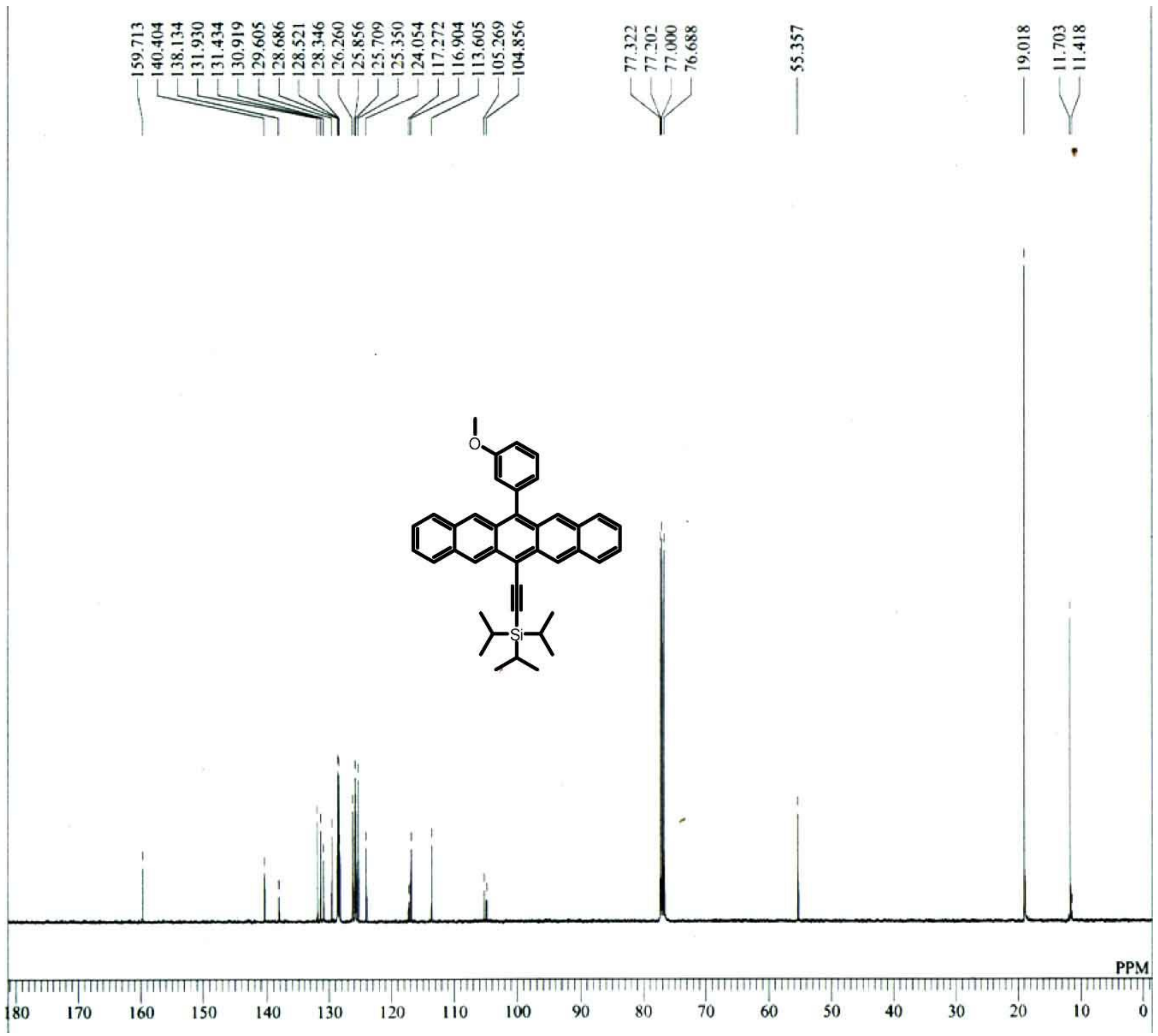

Figure S28: ^{13}C NMR (100 MHz, $CDCl_3$, rt) of pentacene **3e**.

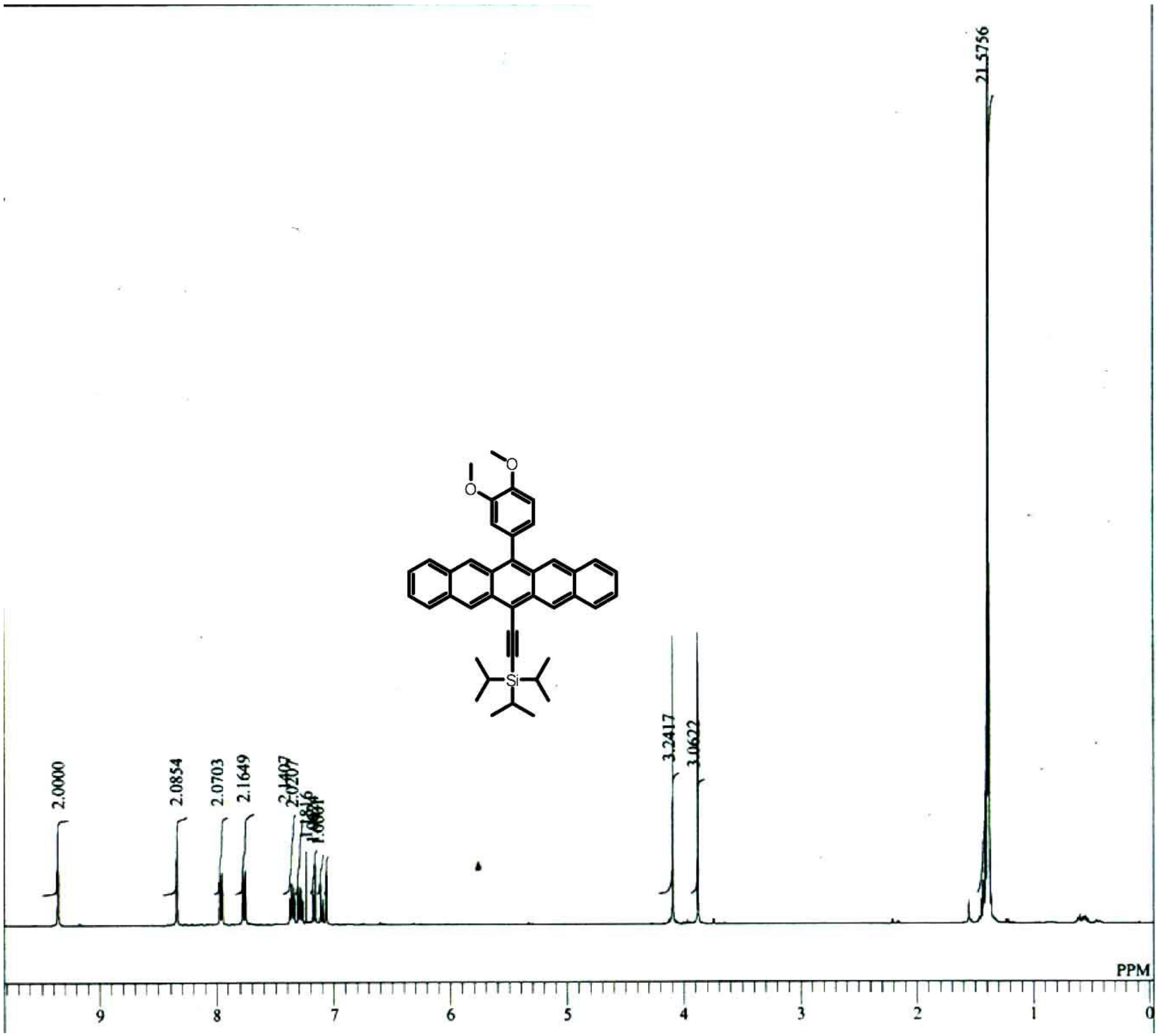

Figure S29: ^{1}H NMR (400 MHz, $CDCl_3$, rt) of pentacene **3f**.

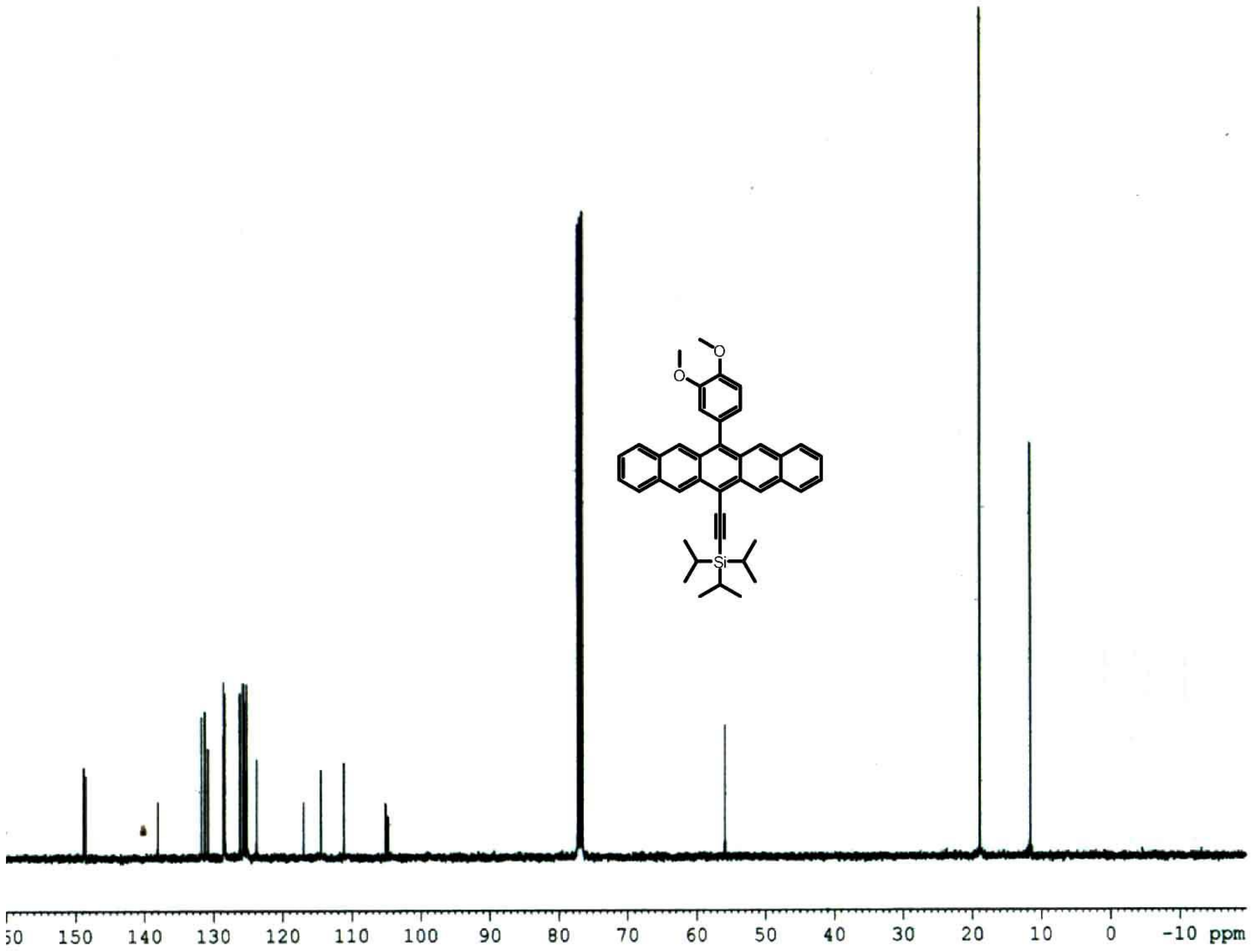

Figure S30: ^{13}C NMR (100 MHz, $CDCl_3$, rt) of pentacene **3f**.

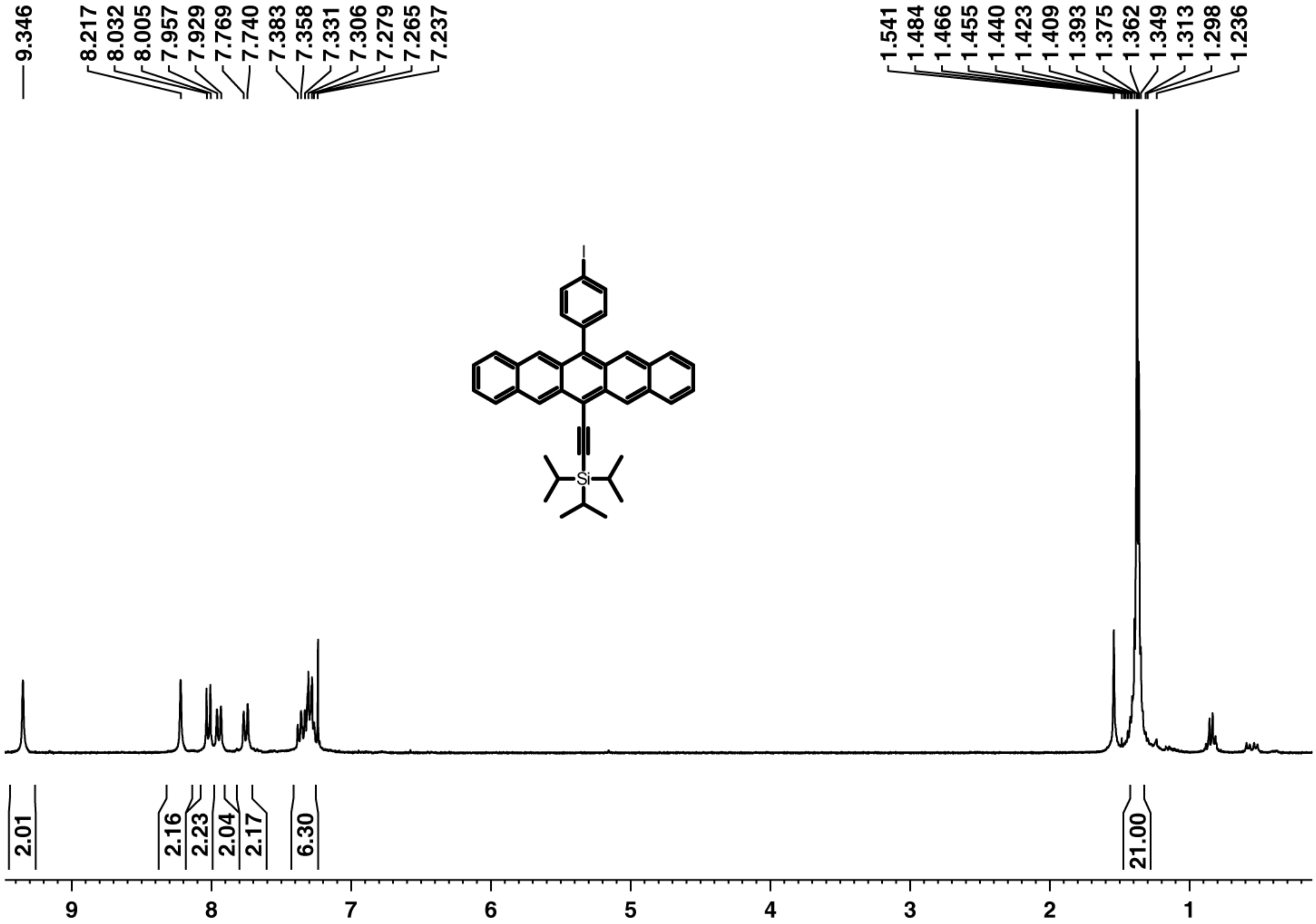

Figure S31: ^{1}H NMR (300 MHz, $CDCl_3$, rt) of pentacene **3g**.

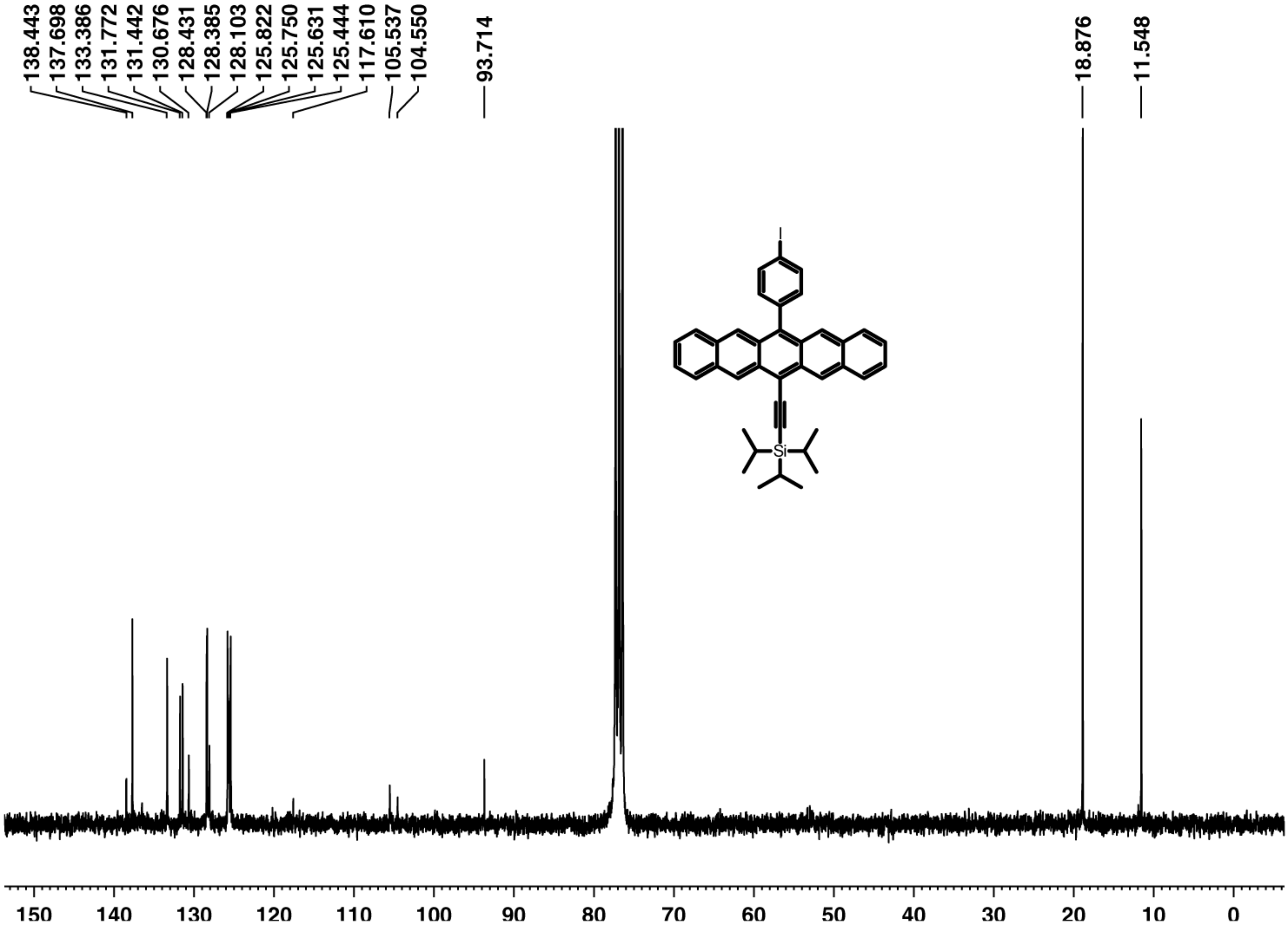

Figure S32: ^{13}C NMR (100 MHz, $CDCl_3$, rt) of pentacene **3g**.

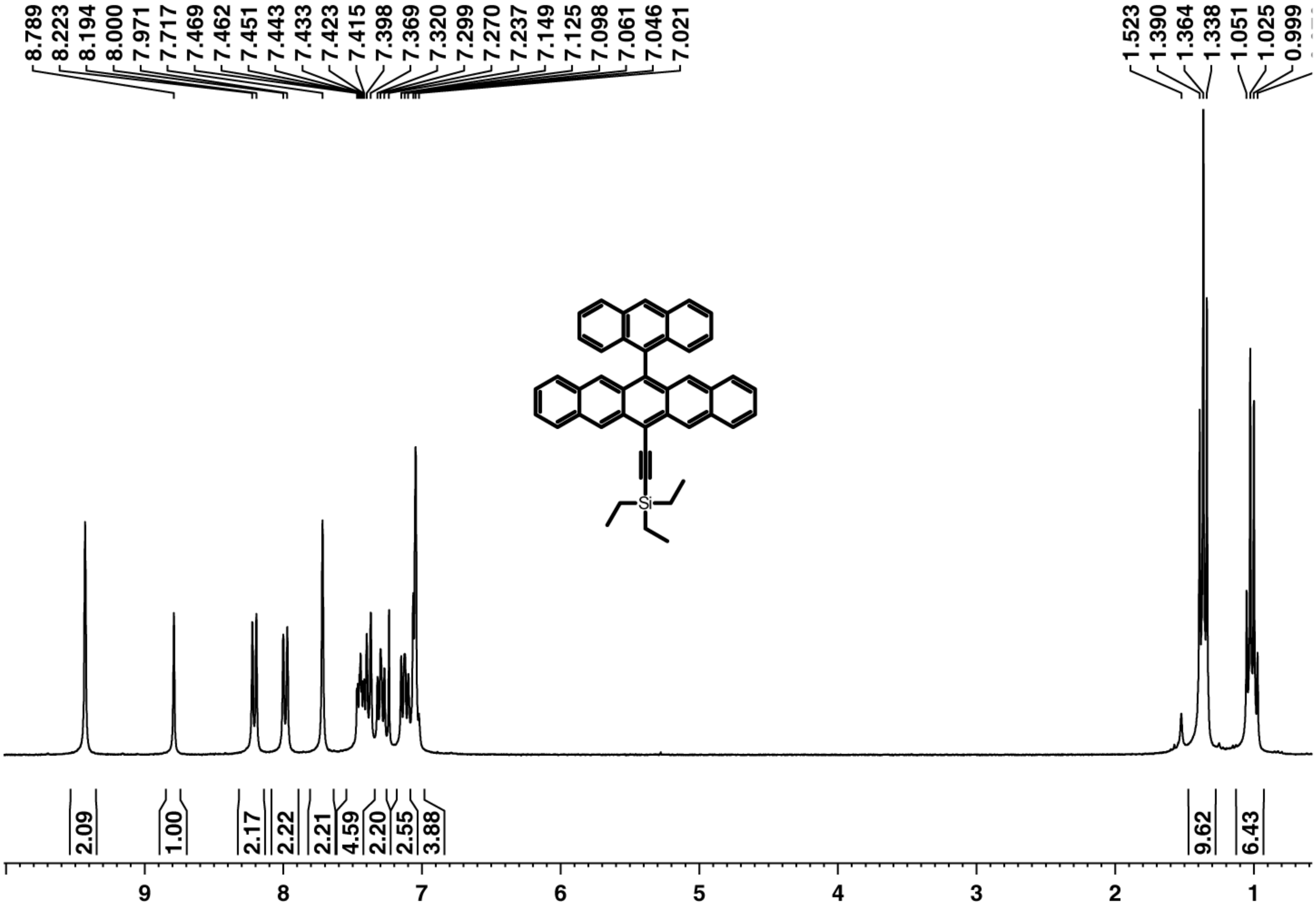

Figure S33: ^{1}H NMR (300 MHz, $CDCl_3$, rt) of pentacene **3h**.

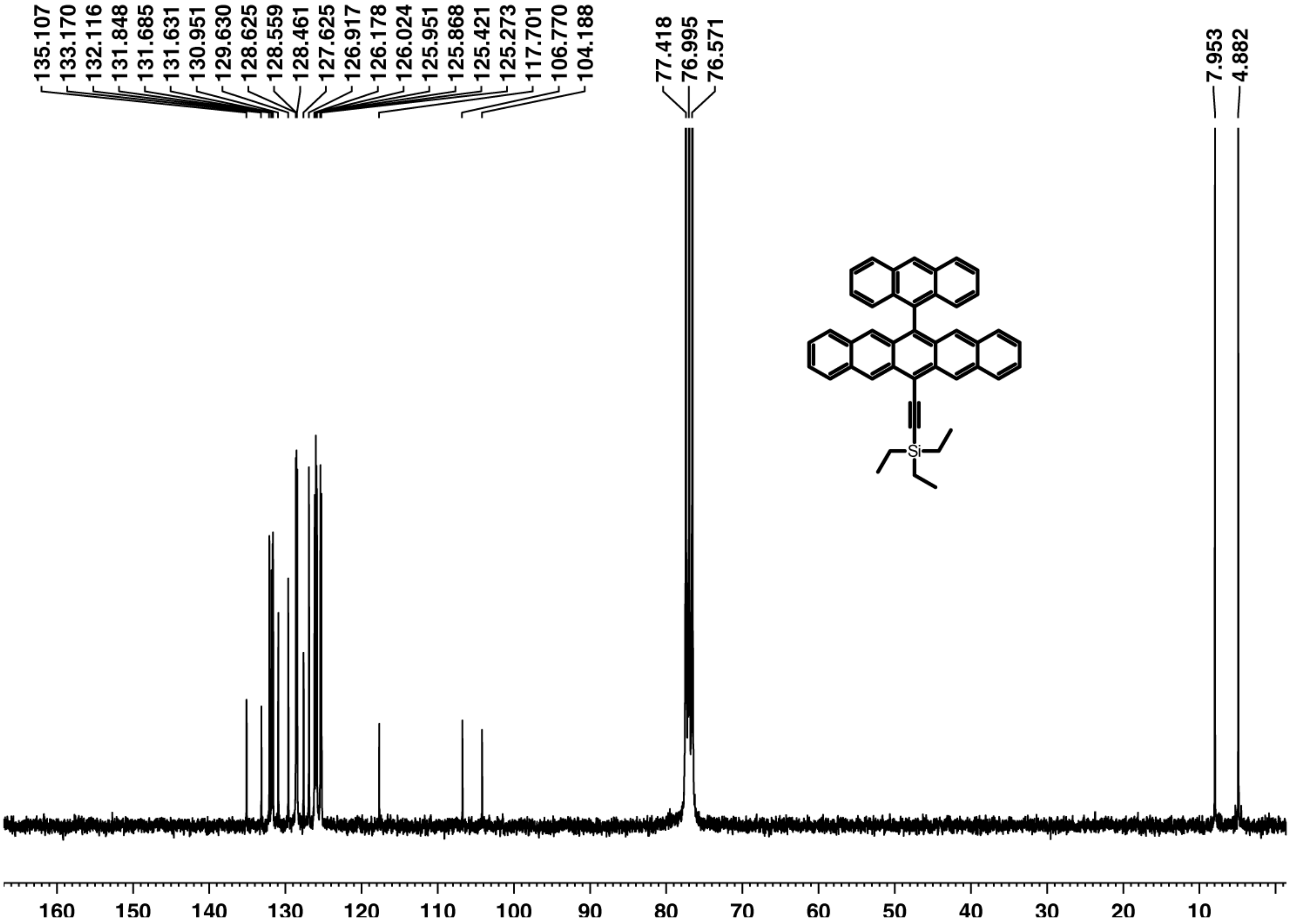

Figure S34: ^{13}C NMR (75 MHz, $CDCl_3$, rt) of pentacene **3h**.

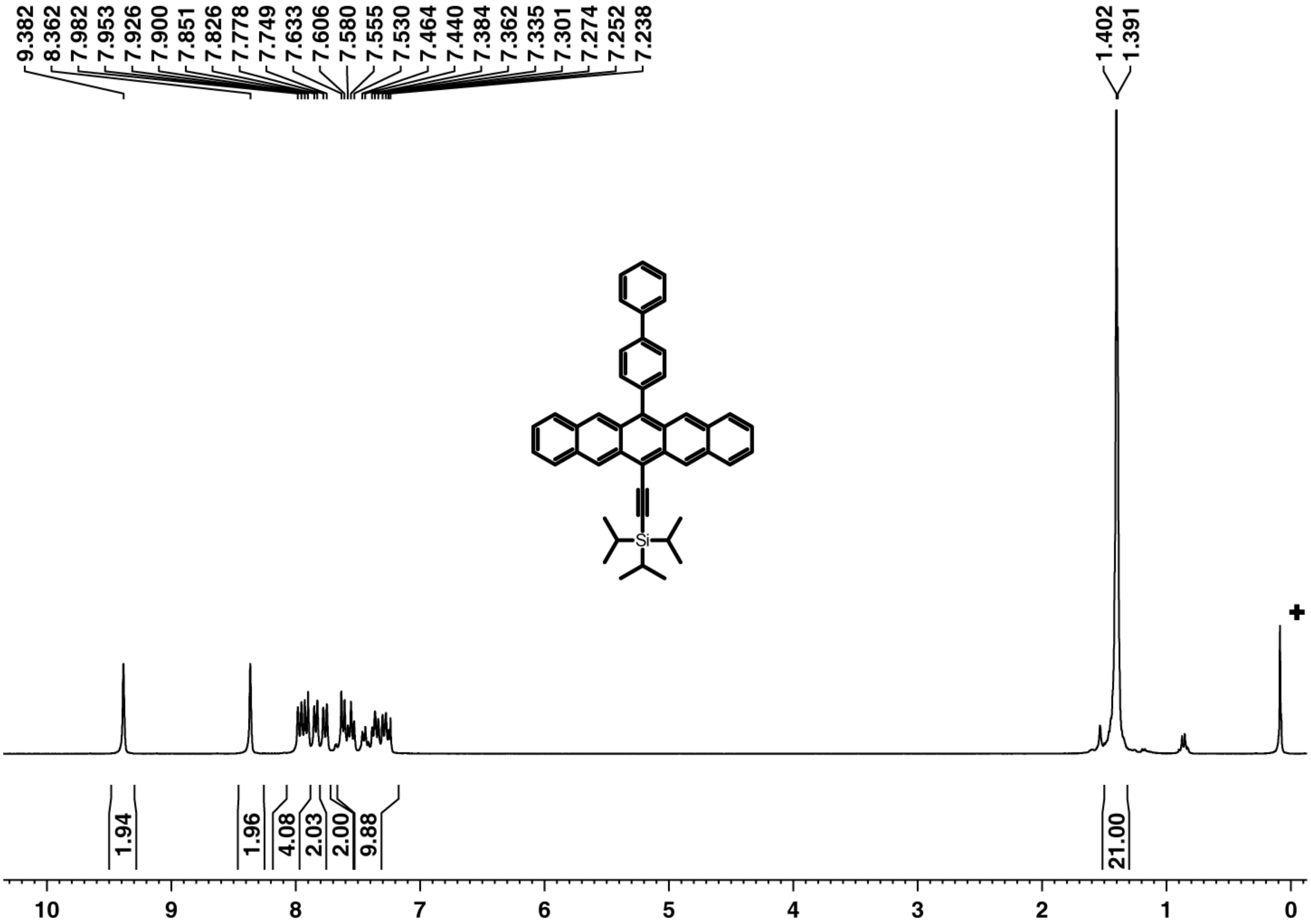

Figure S35: ^{1}H NMR (300 MHz, $CDCl_3$, rt) of pentacene **3i** (**+** = silicon grease).

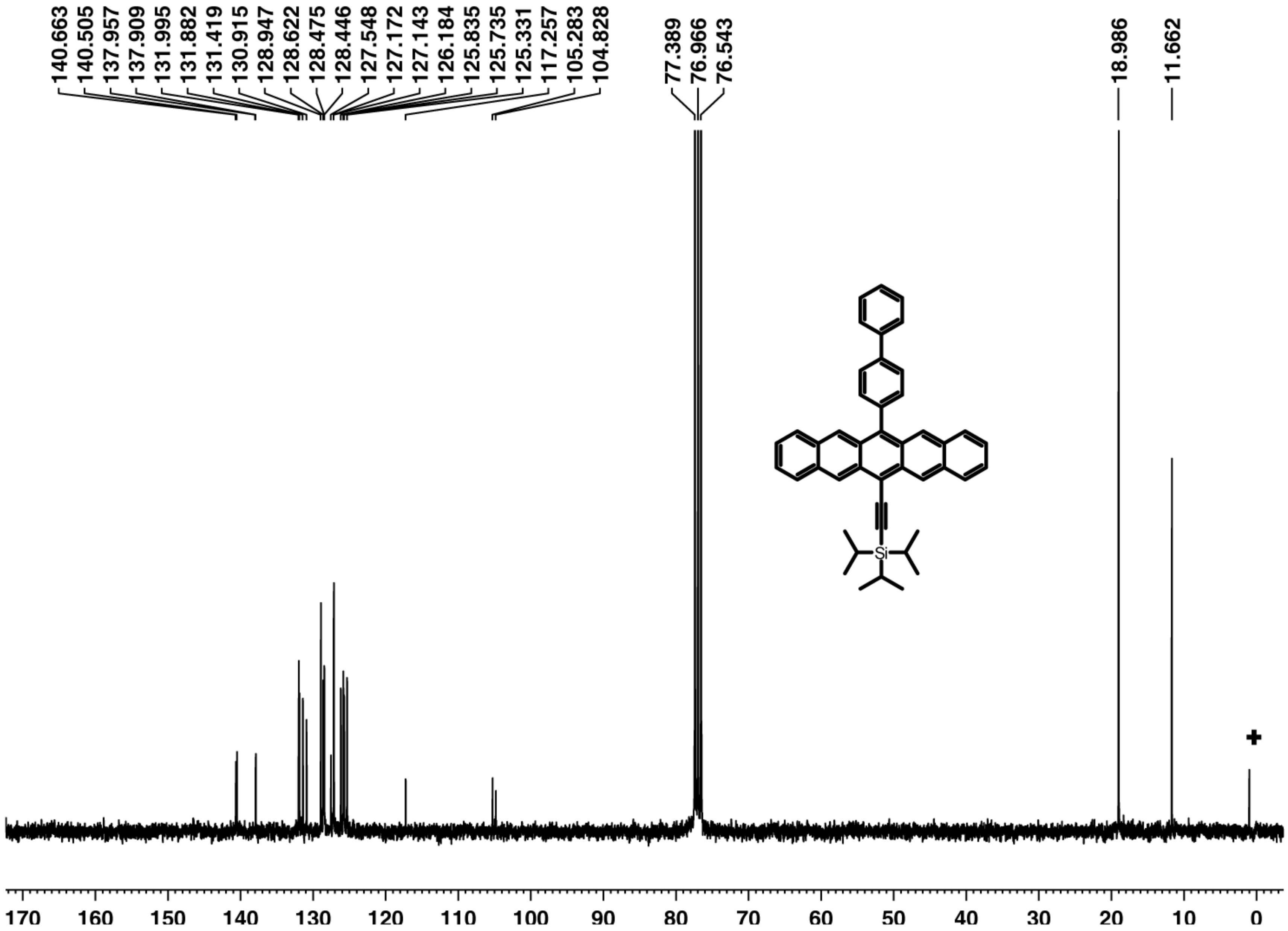

Figure S36: ^{13}C NMR (75 MHz, $CDCl_3$, rt) of pentacene **3i** (**+** = silicon grease).

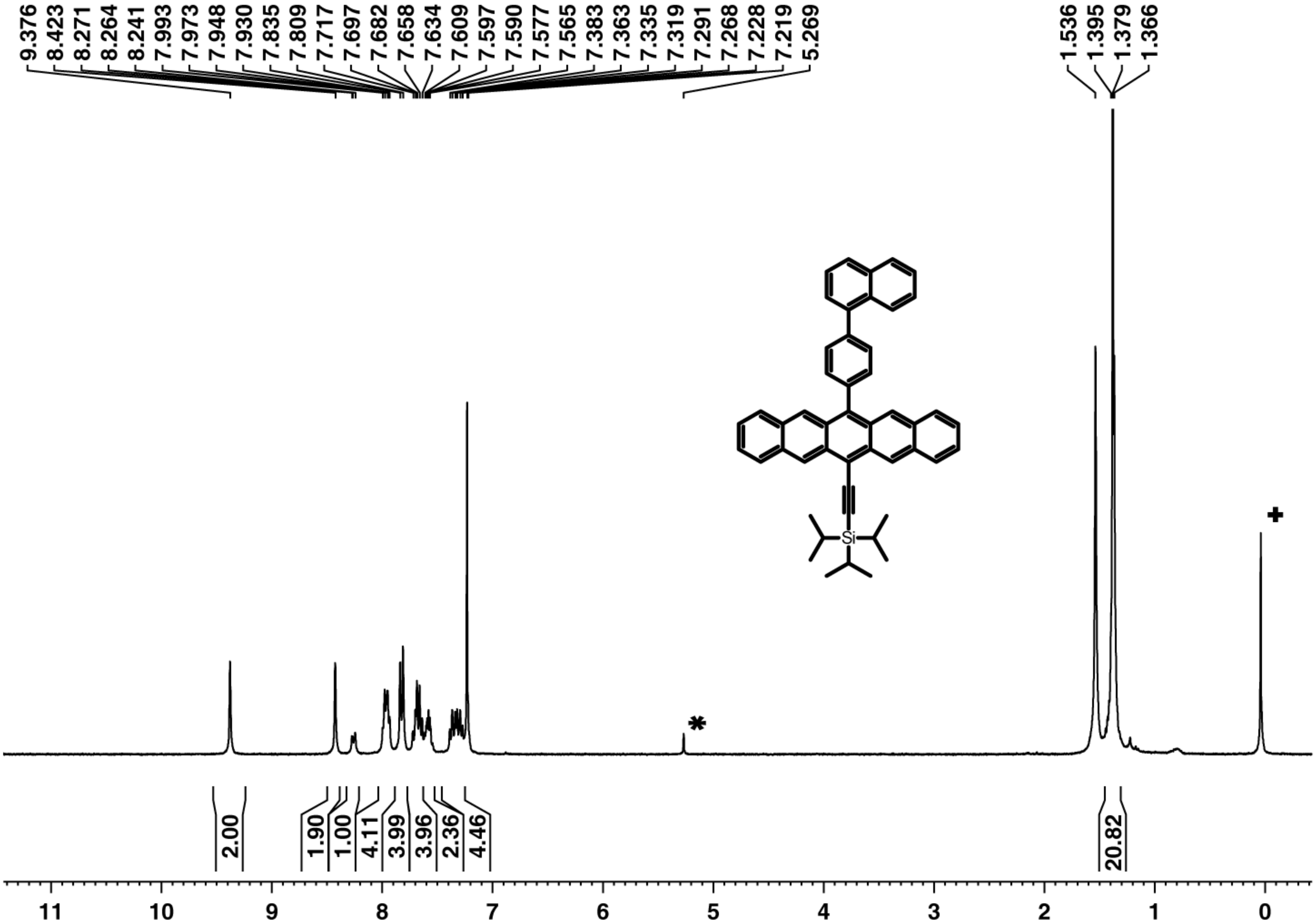

Figure S37: ^{1}H NMR (300 MHz, $CDCl_3$, rt) of pentacene **3j** (***** = CH_2Cl_2;**+** = silicon grease)

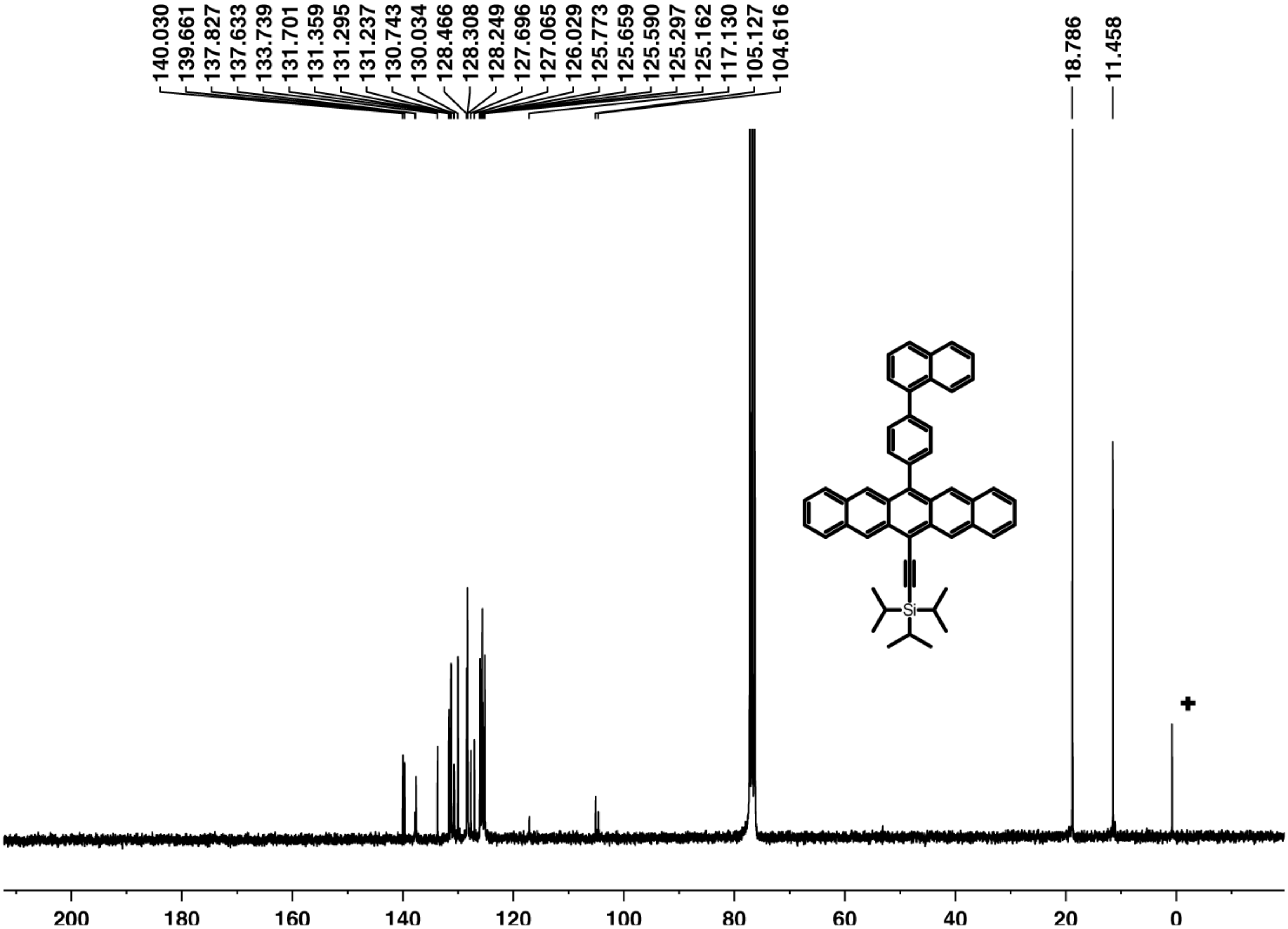

Figure S38: ^{13}C NMR (75 MHz, $CDCl_3$, rt) of pentacene **3j** (**+** = silicon grease).

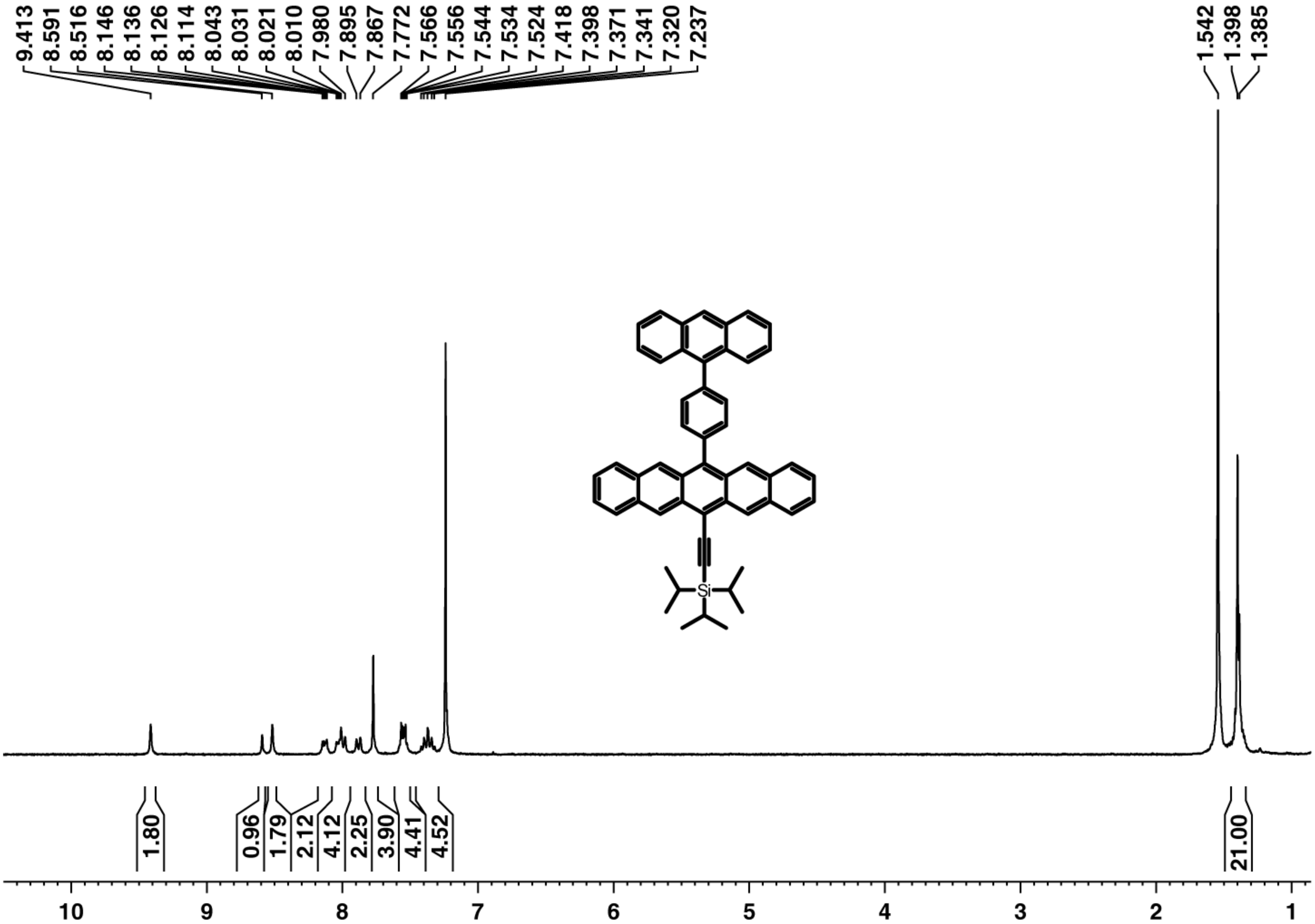

Figure S39: ^{1}H NMR (300 MHz, $CDCl_3$, rt) of pentacene **3k**.

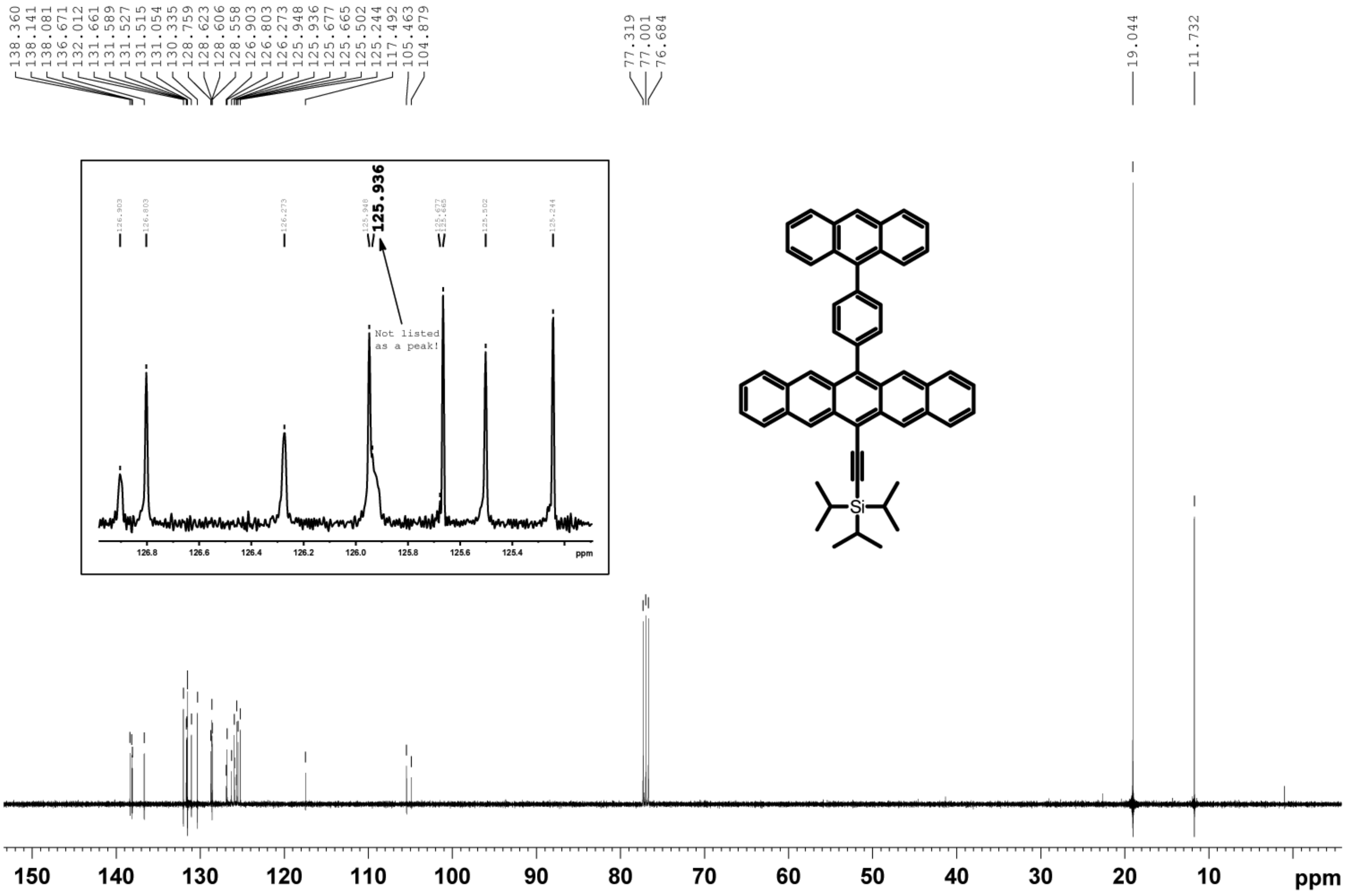

Figure S40: ^{13}C NMR (100 MHz, $CDCl_3$, rt) of pentacene **3k.**
